# Antibiotic origami: selective formation of spirotetronates in abyssomicin biosynthesis†

**DOI:** 10.1039/d4sc03253e

**Published:** 2024-08-12

**Authors:** Sbusisiwe Z. Mbatha, Catherine R. Back, Andrew J. Devine, Hannah M. Mulliner, Samuel T. Johns, Harry Lewin, Kaiman A. Cheung, Katja Zorn, James E. M. Stach, Martin A. Hayes, Marc W. van der Kamp, Paul R. Race, Christine L. Willis

**Affiliations:** a School of Chemistry, University of Bristol Bristol BS8 1TS UK chris.willis@bristol.ac.uk; b School of Biochemistry, University of Bristol Bristol BS8 1TD UK; c Compound Synthesis and Management, Discovery Sciences, Biopharmaceuticals R&D, AstraZeneca Pepparedsleden 1 431 83 Mölndal Sweden; d School of Natural and Environmental Sciences, Newcastle University Newcastle Upon Tyne NE1 7RU UK paul.race1@newcastle.ac.uk

## Abstract

The abyssomicins are a structurally intriguing family of bioactive natural products that include compounds with potent antibacterial, antitumour and antiviral activities. The biosynthesis of the characteristic abyssomicin spirotetronate core occurs *via* an enzyme-catalysed intramolecular Diels–Alder reaction, which proceeds *via* one of two distinct stereochemical pathways to generate products differing in configuration at the C15 spirocentre. Using the purified spirotetronate cyclases AbyU (from abyssomicin C/atrop-abyssomicin C biosynthesis) and AbmU (from abyssomicin 2/neoabyssomicin biosynthesis), in combination with synthetic substrate analogues, here we show that stereoselectivity in the spirotetronate-forming [4 + 2]-cycloaddition is controlled by a combination of factors attributable to both the enzyme and substrate. Furthermore, an achiral substrate was enzymatically cyclised to a single enantiomer of a spirocyclic product. X-ray crystal structures, molecular dynamics simulations, and assessment of substrate binding affinity and reactivity in both AbyU and AbmU establish the molecular determinants of stereochemical control in this important class of biocatalysts.

## Abstract 2

The molecular origins of stereoselectivity in enzyme catalysed Diels–Alder reactions in abyssomicin biosynthesis are determined and spirotetronates prepared with the creation of 3 new stereocentres.

## Graphical abstract

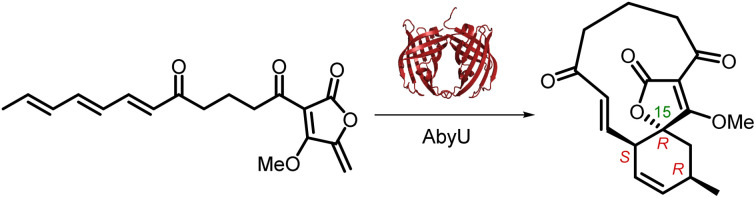

## Introduction

Asymmetric catalysis is of fundamental importance in modern synthetic chemistry.^1^ Enzymes play key roles as chiral catalysts in sustainable synthesis as they are biodegradable and readily replenished through fermentation processes, affording new, efficient, and “green” routes to a wide variety of valuable bioactive compounds.^2^ Despite their advantages, much still remains to be learned about the factors which influence the stereochemical outcome of enzyme-catalysed reactions, considerations which are critical in establishing the feasibility of deploying biocatalysts in synthesis.^3^

**Scheme 1:**
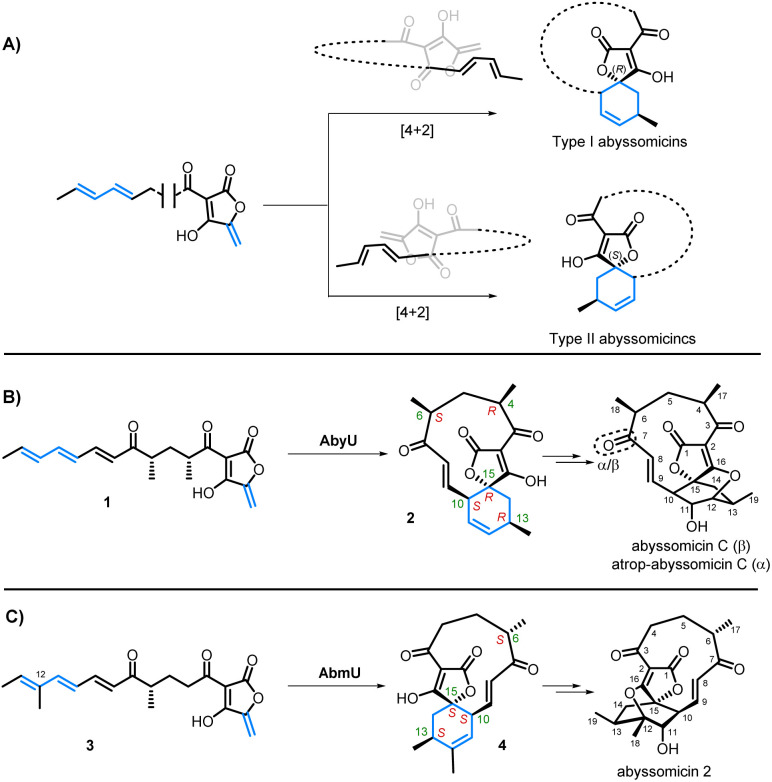
(A) General model of spirocycle formation in Type I and Type II spirotetronates. (B) Late stage biosynthesis of abyssomicin C/atrop-abyssomicin C (Type I). (C) Late stage biosynthesis of abyssomicin 2 (Type II).

The Diels–Alder reaction is a key transformation in organic synthesis in which a diene and dienophile undergo a [4 + 2]-cycloaddition to generate 2 new carbon–carbon bonds and up to 4 new stereocentres in the cyclohexene product. A survey of natural product structures suggest that enzymes catalysing Diels–Alder reactions may be implicated in the biosynthesis of more than 400 natural products.^4^ Indeed, Diels–Alderases have been shown to be responsible for the construction of the spirotetronate framework of the abyssomicins, a family of polyketide natural products which exhibit a range of important biological activities, including antimicrobial, antitumour, and antiviral properties.^5^ The abyssomicins all share similar biosynthetic origins *via* the assembly of a linear polyketide precursor containing an *exo*-methylene tetronate dienophile and a terminal diene tail. A significant divergence in this family then occurs based on the stereochemical outcome of the subsequent intramolecular [4 + 2]-cycloaddition of these linear precursors, leading to two “enantiomeric” sub-families of abyssomicins; type I and type II (Scheme 1A).^6,7^ This is well illustrated by the examples from abyssomicin C (type I) and abyssomicin 2 (type II) biosynthesis. Abyssomicin C/atrop-abyssomicin C are produced by *Micromonospora maris*, and following the [4 + 2]-cycloaddition catalysed by AbyU, spirotetronate 2 has the 15*R* stereochemistry (type I, Scheme 1B).^7^ Subsequent epoxidation of the 11,12-alkene by the cytochrome P450 AbyV and cyclisation gives abyssomicin C and its atropisomer.^8^ Contrastingly, in abyssomicin 2 biosynthesis, the biosynthetic intermediate 3 is thought to undergo a [4 + 2]-cycloaddition catalysed by AbmU to give Diels–Alder product 4 with the 15*S* stereochemistry (type II, Scheme 1C). Epoxidation/cyclisation catalysed by AbmV then gives abyssomicin 2.^9^ In developing these cyclases as biocatalysts for use in synthesis, it is important to understand what controls the contrasting stereochemical outcomes of these two Diels–Alder reactions. There are key structural differences between the two biosynthetic intermediates 1 and 3. The AbmU substrate 3 has an additional methyl group in the diene portion of the molecule (at C-12) compared with the natural substrate 1 for AbyU. Furthermore, the C_11_-macrocycle of abyssomicin C possesses two methyl substituents (at C-4 and C-6), whereas abyssomicin 2 has a single methyl group (at C-6), both having the 6*S* configuration. The interaction of these substrates with the cyclases is a further important factor to be considered. Herein we report the use of a combination of X-ray crystallography, organic synthesis, bioassays, and molecular simulations to gain insights into how AbmU and AbyU achieve differing stereochemical outcomes, as well as the effect of substrate structure on the products formed in non-enzymatic thermal Diels–Alder reactions.

## Results and discussion

To begin, AbmU and AbyU were recombinantly over-expressed in *E. coli* BL21(DE3) cells and purified to homogeneity (Fig. S1†). Both polypeptides were found to be dimeric, well-folded, monodispersed species in solution (Fig. S1†).

In our previous studies of AbyU and structurally related cyclases, including Cyc15, we had found them to be co-factor independent, standalone enzymes.^10^ This is consistent with the Liu's groups studies on the related spirotetramate cyclase PyrI4.^11^ In contrast to these findings, Li *et al.* have recently reported that AbmU binds heme b in a non-specific manner.^6*b*^ Intrigued by this unexpected result, we investigated the capacity for AbmU to bind heme during protein expression and purification, including through supplementation of the growth media with 4-aminolevulinic acid and/or hemin (Fig. S1b†). No evidence for heme binding by AbmU was observed in these studies, which contrasts with the previous reported findings by Li *et al.*^6*b*^

**Fig. 1:**
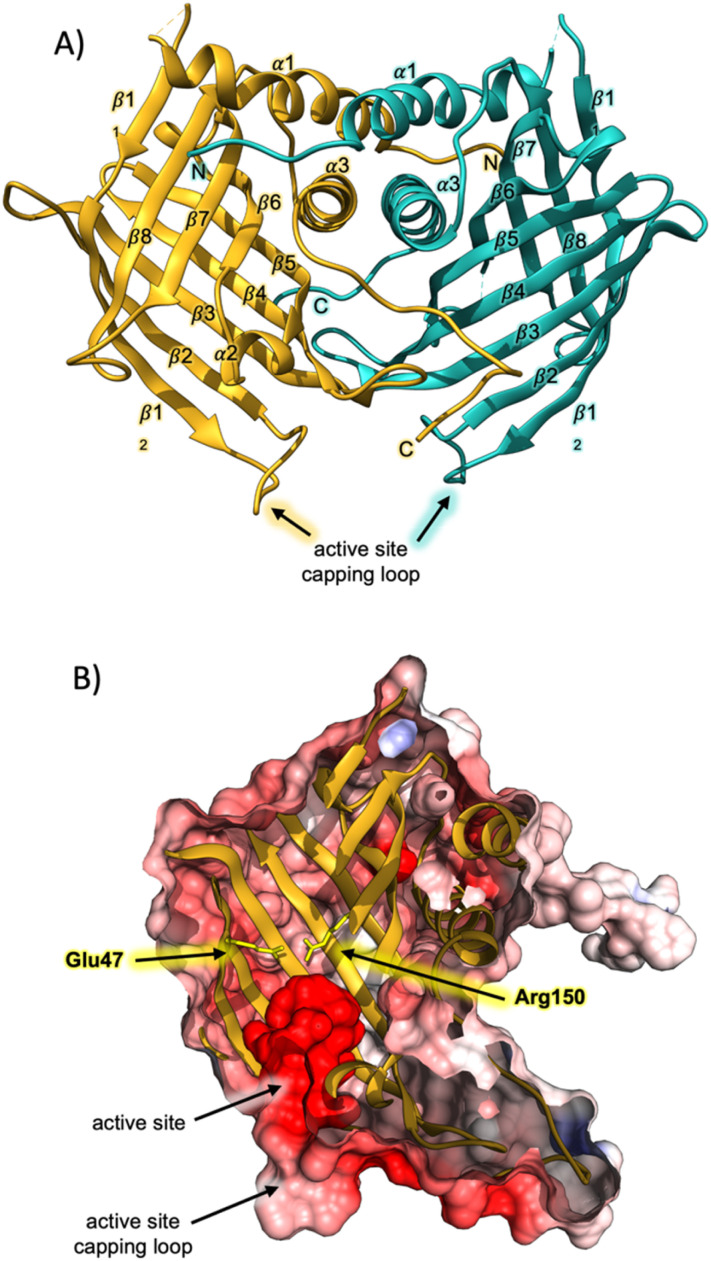
Crystal structure of AbmU. (A) Overall fold of the AbmU dimer. Monomers are coloured gold and teal, respectively. (B) Cut-through view of the AbmU crystal structure surface, coloured by electrostatic potential, revealing the enzyme active site. The two residues Glu47 and Arg150 that form the salt bridge are shown in stick format and coloured yellow.

Having successfully produced recombinant AbmU, we next proceeded to elucidate its crystal structure, which was determined to a resolution of 2.05 Å using the single wavelength anomalous dispersion method as applied to 1 M NaBr-soaked crystals (ESI methods and Table S1†). AbmU is a homodimer, with each monomer comprised of an eight-stranded antiparallel β-barrel, decorated with three short α-helices and associated loop regions (Fig. 1A). Dimerisation is predominantly facilitated by hydrophobic interactions at the dimer interface, and is further stabilised by the N and C terminal strands of each monomer, which partially wrap around their respective dimer partners. The central barrel is sealed at one end by a salt-bridge, analogous to that observed in the structure of AbyU,^12^ and capped at the other by a loop, formed by the β1_2_–β2 linker. In AbyU, this loop has been shown to be flexible by molecular dynamics simulations, regulating access to the enzyme active site. The capping loop is more open in the AbmU structure as compared to that of AbyU, though there is close structural alignment between the β-barrel elements of both proteins (Fig. S2†).^12^ Although AbmU and AbyU share 39% sequence identity and exhibit analogous β-barrel folds, important structural differences are observed that differentiate the two polypeptides. Firstly, each AbmU monomer possesses an additional three α-helices as compared to AbyU, and secondly, the two monomers that constitute the AbmU dimer adopt orientations that are distinct from those seen in the AbyU structure.§§The atomic coordinates and structure factors (code 6YMN) have been deposited in the Protein Data Bank.

The hydrophobic cavity of the AbmU barrel lumen constitutes the active site of the enzyme (Fig. 1B), and is comprised of the residues Val49, Leu51, Asp67, Tyr69, Met102, Tyr104, Val121, Ile126, Gln132, Phe134 and Phe152 (Fig. S2†). Clearly resolvable electron density is observed for all the amino acid side chains within this region, enabling the structure of the AbmU active site to be unambiguously defined (Fig. 1 and S2†). The side-chains of the identified residues collectively establish an active site architecture with appropriate shape complementarity to match that of the transition state required to form intermediate 4. This is fully consistent with a proposed mode of catalysis where the enzyme functions to promote conformational constriction of the linear substrate 3, to spatially co-locate the substrate diene and dienophile moieties, enabling cyclisation to proceed. The shape of the AbmU active site pocket diverges from that of AbyU, consistent with the different stereochemical outcomes in each enzyme's respective [4 + 2]-cycloaddition reaction. The predominately hydrophobic nature of the AbmU active site provides a suitable platform for substrate binding, without overtly compromising product dissociation.

**Scheme 2:**
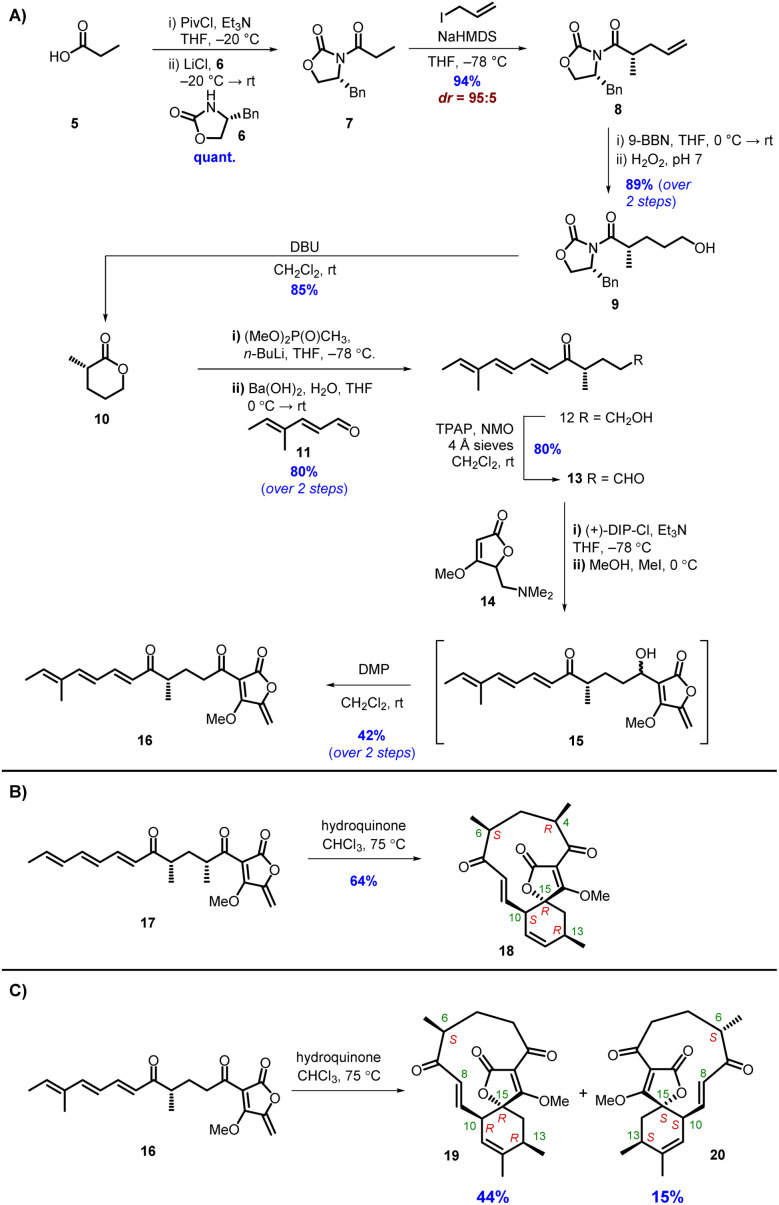
(A) Synthesis of AbmU substrate 16. (B) Thermal cycloaddition of AbyU substrate 17. (C) Thermal cycloaddition of AbmU substrate 16.

To probe the stereochemical outcomes of the enzymatic and non-enzymatic reactions, synthetic samples of the linear precursors from both the type I and type II pathways were prepared. Tetronic acids readily tautomerise, leading to problems when characterising products by NMR spectroscopy, hence the analogous methyl tetronates were selected as the targets. The methyl tetronate analogue 16 of the natural AbmU substrate 3 was synthesised according to the approach outlined in Scheme 2A. Chiral lactone 10 was prepared in 5 steps starting from Evans' (*R*)-oxazolidinone 6. Alkylation with allyl iodide established the required (*S*)-stereocentre with excellent yield and diastereoselectivity. Hydroboration-oxidation gave alcohol 9 which was then treated with DBU to promote cyclisation to give lactone 10 in 72% overall yield from 6 (Scheme 2A). Ring-opening of lactone 10 by lithiated dimethyl methylphosphonate followed by a Horner–Wadsworth–Emmons reaction with aldehyde 11 (ref. 13) gave alcohol 12 in 80% yield from lactone 10. Various conditions were investigated for the oxidation of alcohol 12 to aldehyde 13 including TEMPO/BAIB, Dess Martin periodinane (DMP) and Stahl oxidation, but the most reliable method proved to be Ley-Griffiths conditions.^14^ Aldehyde 13 was coupled with tetronate 14 in the presence of (+)-DipCl and Et_3_N, then addition of CH_3_I quaternised the amine and subsequent elimination gave alcohol 15 as a mixture of diastereomers which were used directly in the final step. Oxidation of 15 with DMP gave the required product 16 in 42% yield from aldehyde 13. Neat 16 was prone to degradation in air at room temperature, necessitating storage as a dilute solution in THF. The methyl tetronate analogue 17 of the natural AbyU substrate 1 was prepared as previously described.^8^

In accordance with previous reports,^12,15^ heating 17 in CHCl_3_ in the presence of hydroquinone gave a single cycloadduct 18 (Scheme 2B), indicating strong substrate-derived stereocontrol favouring the type I product (15*R*). Under the same reaction conditions trienone 16 gave 2 new products by LC-MS in a 3 : 1 ratio, both with the same molecular mass ([M + Na]^+^ = 345) as the starting material (Scheme 2C). The products were purified by normal-phase HPLC and their structures elucidated by extensive NMR studies. It was evident that diastereomeric spirotetronates had been formed *via* intramolecular [4 + 2] cycloadditions. The ^1^H and ^13^C NMR spectra of the major diastereomer showed significant similarities to those of 18 formed from heating 17 with 2 methyl groups in the linear chain *i.e.* a type I folding pattern (Scheme 2C). The structure of 19, with the 6*S*,13*R*,15*R* stereochemistry, was confirmed by NOE studies, with irradiation of the signals assigned to 6-H, 10-H, 13-H and the methoxy group being particularly informative (Fig. S3, and Table S2†).

The ^1^H-NMR spectrum of the minor product 20 was distinctly different from the major product in the olefinic region. In the minor product 8-H resonated at *δ*_H_ 6.22 (d, *J* 16.5 Hz) and 9-H at *δ*_H_ 5.98 (dd, *J* 16.5, 10.5 Hz). Contrastingly, in the major product 19, the signal assigned to 8-H appeared at *δ*_H_ 6.44 (d, *J* 16.5 Hz) and 9-H at *δ*_H_ 6.38 (dd, *J* 16.5, 10.5 Hz). Following NOE studies, the structure of the minor product 20 was confirmed to be a type II abyssomicin with the 6*S*,13*S*,15*S* stereochemistry (Fig. S3 and Table S3†). From these results it was clear that the different positioning of the methyl substituents in the two compounds were having a significant effect on the stereochemical outcome of the [4 + 2] cycloaddition. The presence of the C-4 and C-6 methyl groups in 17 evidently impart a significant preference for the type I folding, which could be rationalised on the basis of these substituents being positioned in a pseudoequatorial fashion that minimises unfavourable steric interactions in the required transition state (Fig. S4†). This effect is somewhat diminished for 16 where the C-4 methyl substituent is no longer present, leading to the ratio of products observed.

**Fig. 2:**
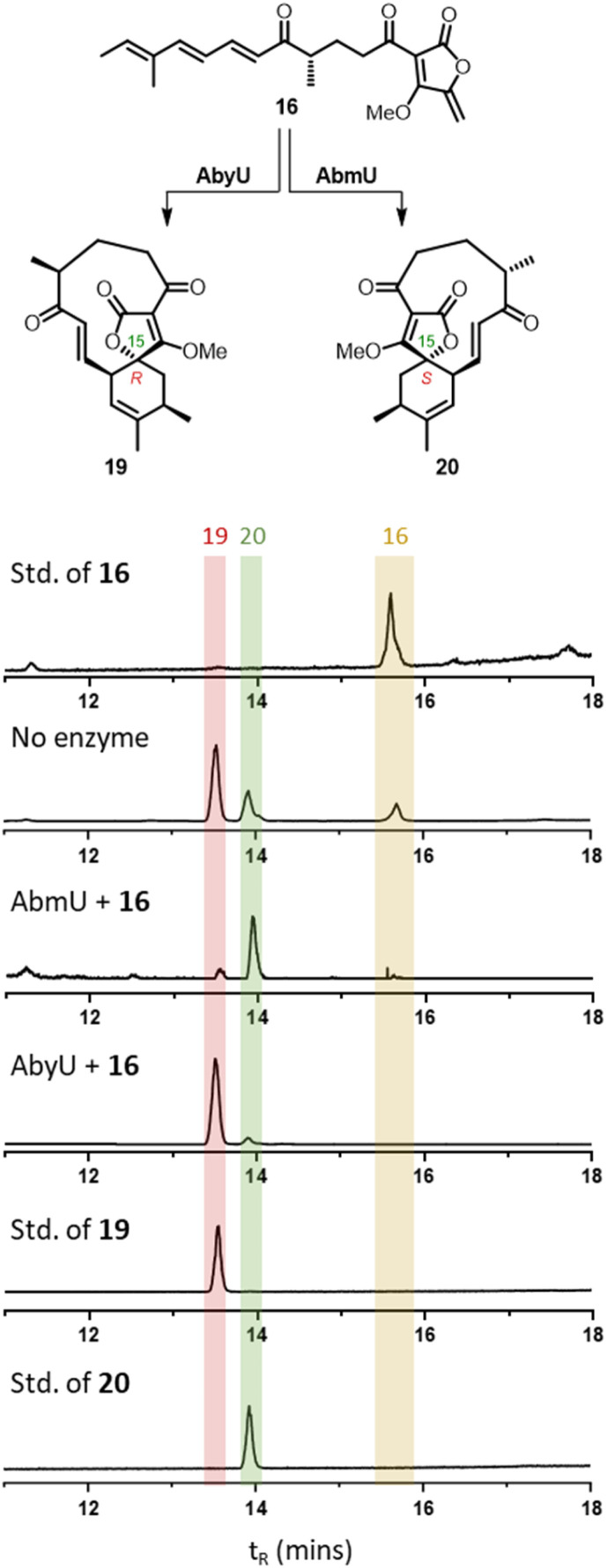
LC-MS ELSD chromatograms showing Diels–Alder products arising from incubation of 16 with AbmU and AbyU.

Next 16 and 17 were used as substrates to explore the selectivity of the cyclases AbmU and AbyU. Beginning with the AbmU precursor 16, *in vitro* assays were carried out with each enzyme, incubating 16 (5 mM) with each respective enzyme in buffer (20 mM Tris–HCl, 150 mM NaCl, pH 8.0) for 10 minutes at ambient temperature. The reactions were monitored by LC-MS and compared with synthetic standards of 19 and 20 for reference as summarised in Fig. 2. Incubation of 16 with AbmU gave a single peak with a retention time and mass corresponding to type II product 20, whilst the AbyU reaction showed a peak with a retention time which corresponded to the type I product 19. Both assays were scaled up, the products isolated and the respective structures confirmed by NMR and comparison with synthetic standards (Fig. S5 and S6†). These results were consistent with the proposed natural function of the two enzymes in setting the type I and type II stereochemistry respectively. We proceeded to explore the AbyU precursor 17 as the substrate in *in vitro* assays with AbmU and AbyU and in both cases the same product 18 (Type I, *t*_R_ = 13.9) was observed by LC-MS (Fig. S8†).

**Fig. 3:**
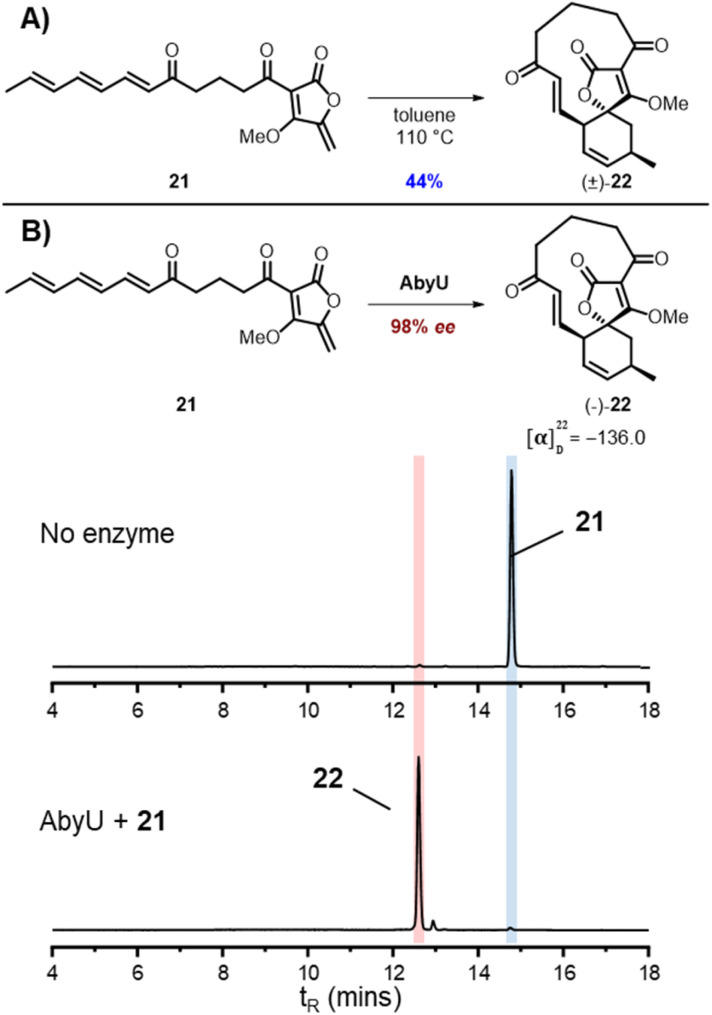
Cycloadditions of achiral substrate analogue 21 (A) Thermal cycloaddition of 21. (B) Enzymatic cycloaddition of 21 with AbyU with LC-MS ELSD chromatograms showing conversion to 22.

From these studies it was clear that AbmU could overcome the preference for the natural substrate analogue 16 to cyclise with 15*R* stereochemistry observed under thermal conditions, indicating it can selectively bind the pro-15*S* conformation of its natural substrate to achieve stereocontrol. This selectivity was mostly diminished with the non-natural AbyU substrate analogue 17, suggesting the enzyme has evolved closely with its natural substrate to achieve this specialisation. This is consistent with the loss of selectivity reported for the homologous enzyme Plo4 when incubated with a related but non-natural substrate.^16^ In contrast, from these results any stereocontrol exerted by AbyU remained largely ambiguous, as the outcomes with this enzyme favoured the major product from thermal cycloadditions with both 16 and 17. While previous *in silico* studies had suggested that AbyU does indeed selectively bind a specific reactive conformation of its natural substrate,^12^ we wished to obtain *in vitro* evidence that AbyU could act as a stereoselective catalyst. Hence, we synthesised the achiral substrate analogue 21 lacking the methyl substituents present in both 16 and 17, with a view to examining whether AbyU could achieve enantioselective [4 + 2]-cycloaddition (Fig. 3 and S9†). Upon heating in toluene, 21 was converted to racemic cycloadduct 22 as a single diastereomer with the expected *endo* stereochemistry confirmed by NOE analysis. 21 was also readily accepted as a substrate by AbyU, giving cycloadduct (−)-22 as confirmed by ^1^H and ^13^C NMR (Fig. 3 and S10†). Analysis of the AbyU assay extract by chiral HPLC (Fig. S11†) revealed the AbyU-catalysed transformation delivered (−)-22 in >98% ee. Comparison of experimental and calculated ECD spectra suggested the product formed was the 15*R* enantiomer (details in ESI†). These results indicate that AbyU enzyme–substrate interactions can dictate the stereochemical course of the [4 + 2] cycloaddition as well as significantly enhancing the reaction rate.

**Fig. 4:**
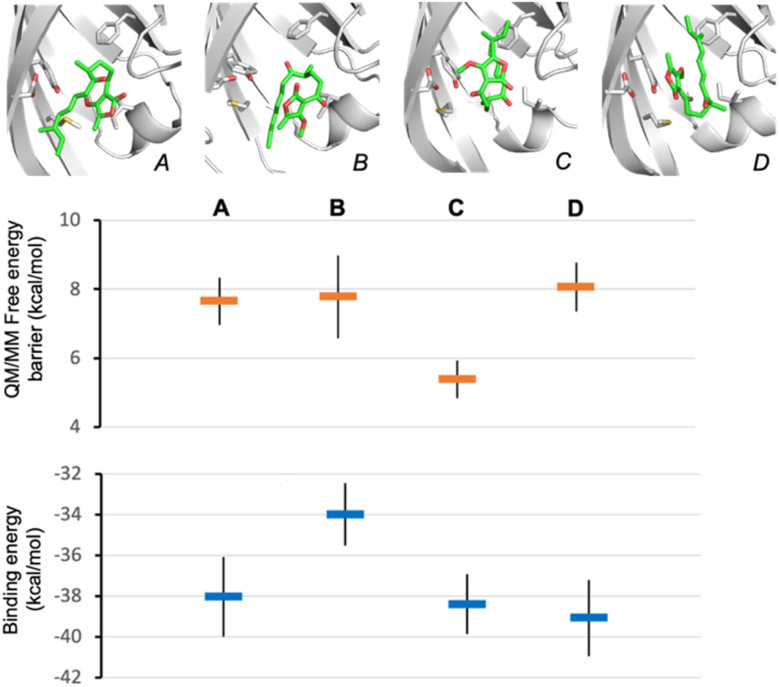
Establishing the likely reactive binding pose of 16 in AbmU for formation of 20. Representative substrate binding modes of 16 predicted from QM/MM reaction simulations starting from product 20 are shown (A to D), together with their predicted Diels–Alder activation free energies (obtained from 10 independent QM/MM umbrella sampling MD simulations using DFTB2/ff14SB) and average binding energies (calculated using MM-GBSA across 10 independent MD simulations) for each.

To establish the likely reactive binding pose of substrate 16 in AbmU, we first remodelled the capping loop of AbmU in a closed conformation (see ESI†). We then generated starting structures for reaction simulations through molecular docking of the major Diels–Alder product produced by AbmU (20, see Fig. 2), identifying four distinct possible poses. Reaction simulations were then performed starting from these poses (using QM/MM umbrella sampling simulations at the DFTB2/ff14SB level, using previously established protocols for AbyU^17^), generating substrate binding poses A–D (Fig. 4). The calculated free energy barriers for reaction indicate that only pose C is consistent with efficient turnover. Notably, pose C (and to a lesser extent, pose D) is similar to the established reactive binding pose of 1 (and its *O*-methylated variant 17) in AbyU. Ten independent short MD simulations were then carried out for each of the four alternative substrate binding poses and approximate binding energies obtained using MM/GBSA calculations, indicating that poses A, C and D bind with similar affinity to the AbmU active site (Fig. 4), consistent with the three competitively binding substrate poses expected in AbyU.^17^ Average distances of the carbon–carbon bonds from these MD simulations confirm that the substrate is held in a reactive position for pose C (average C–C distance 3.53 ± 0.04 Å), in contrast to the other poses (4.68, 4.44 and 3.80 Å, for poses A, B, D, respectively, Fig. S14†).

Subsequently, we conducted MD simulations of the expected reactive substrate binding modes for 16 in AbmU and in AbyU that would lead to either 19 (15*R*) or 20 (15*S*). Average binding affinities and bond-forming C–C distances were again obtained from ten MD simulations for each pose (Fig. S15†). For AbmU, both poses share similar average C–C distances and hence their relative reactivity may be equivalent. However, the pro-15*S* conformation has a much higher binding affinity, indicating a greater likelihood that this pose is adopted and formation of the 15*S*-product is favoured. In contrast, in AbyU, the data indicate that the pro-15*S* and pro-15*R* binding modes have similar affinity (within error), but the pro-15*R* conformation has a significantly shorter average C–C distance. This is consistent with higher reactivity (smaller free energy barrier for reaction) and consistent with the established preference for formation of the 15*R* product 19 from 16 by AbyU (Fig. 2). Interestingly, these results indicate that the preference for 15*S* product formation by AbmU may be due to a preference for 16 binding in a pro-15*S* conformation, whereas preference for *R* product formation by AbyU may be due to more efficient turnover of the pro-*R* conformation. Equivalent MD simulation and analysis of pro-15*R* and pro-15*S* poses of 17 in AbyU and AbmU indicate that for this substrate, the preference for *R*-product formation (18) is likely due to more efficient turnover of the pro-*R* conformation in both enzymes (shorter average C–C distances are observed, Fig. S16†).

## Conclusions

This study establishes the molecular origins of stereoselectivity in the key enzyme catalysed Diels–Alder reaction of abyssomicin biosynthesis. Differing configurations at the C15 spirocentre in type I (abyssomicin C) and type II (abyssomicin 2) compounds yield divergent scaffolds with disparate bioactivities. The findings presented herein thus provide important fundamental insights into biocatalytic [4 + 2] cycloadditions in natural product pathways, and establish a structural framework for the design and engineering of Diels–Alderases for use as tools in asymmetric synthesis. Comparative *in vitro* enzyme assays employing recombinant AbyU and AbmU, in combination with synthetic substrate analogues from both type I and type II pathways and an achiral substrate, demonstrate distinct stereochemical outcomes in the enzyme catalysed reactions. Elucidation of the AbmU crystal structure, reaction and comparative dynamics simulations with AbyU demonstrate that stereocontrol in these biocatalysts is influenced by a combination of factors attributable to both the enzyme and substrate. This work expands our growing understanding of spirotetronate biosynthetic pathways and in particular the critical role of naturally evolved Diels–Alderases in defining the structures and functions of this important class of compounds.

## Data availability

The data supporting this article have been reported as part of the ESI.†

## Author contributions

M. W. v. d. K., M. A. H, P. R. R. and C. L. W. designed the experiments. C. R. B. cloned, over-expressed, purified and crystallised AbmU, and solved the crystal structure. C. R. B. over-expressed and purified AbyU. S. Z. M., A. J. D. and H. M. M. conducted substrate and product syntheses. S. Z. M., A. D. and K. Z. performed enzyme assays and collected and analysed structural data on the products. M. W. v. d. K., S. T. J. and H. L. performed and analysed the molecular modelling and reaction simulation studies. K. A. C. performed structure optimisation and TD-DFT calculations. J. E. M. S. provided reagents and supervision. S. Z. M., C. R. B., A. J. D., M. W. v. d. K., P. R. R. and C. L. W. prepared the manuscript, with input from all the authors. M. A. H., M. W. v. d. K., P. R. R and C. L. W. supervised the experimental work. M. A. H., C. L. W., M. W. v. d. K and P. R. R. acquired funding.

## Conflicts of interest

The authors declare there are no conflicts of interest.

## Supplementary Material

SC-OLF-D4SC03253E-s001
