# Supplementary material for: Antibiotic origami: selective formation of spirotetronates in abyssomicin biosynthesis
Source: Chem Sci. 2024 Aug 12;15(34):14009–15. doi: 10.1039/d4sc03253e (PMC11318650; doi:10.1039/d4sc03253e)
Supplement: SC-015-D4SC03253E-s001 [file SC-015-D4SC03253E-s001.pdf]

Supplementary Information (SI) for Chemical Science.
This journal is © The Royal Society of Chemistry 2024

Antibiotic Origami: Selective Formation of Spirotetronates in Abyssomicin Biosynthesis

Sbusisiwe Z. Mbatha,[#a] Catherine R. Back,[#b] Andrew J. Devine,[a] Hannah M. Mulliner,[a] Samuel T. Johns,[b] Harry Lewin,[b] Kaiman A. Cheung,[a] Katja Zorn,[c] James E. M. Stach,[d] Martin A. Hayes,[c] Marc W. van der Kamp,[b] Paul R. Race,[b,d] and Christine L. Willis[a]**

a. School of Chemistry, University of Bristol, Bristol, BS8 1TS (UK)
b. School of Biochemistry, University of Bristol, Bristol, BS8 1TD (UK)
c. Compound Synthesis and Management, Discovery Sciences, Biopharmaceuticals R&D, AstraZeneca, Pepparedsleden 1, SE-431 83, Mölndal (Sweden)
d. School of Natural and Environmental Sciences, Newcastle University, Newcastle Upon Tyne, NE1 7RU (UK)

# Contributed equally to this work.
* Corresponding authors: paul.race1@newcastle.ac.uk; chris.willis@bristol.ac.uk

Contents

1. Experimental Procedures ... 2

1.1 Gene Cloning ... 2

1.2 Protein Expression and Purification ... 3

1.3 Protein Crystallisation and Heavy Atom Soaking ... 3

1.4 Diffraction Data Collection and Structure Determination ... 3

1.5 Electrostatic Potential Calculations ... 3

1.6 Chemical Synthesis ... 3

1.7 *In vitro* Enzyme Assays ... 13

1.8 LC-MS Analysis ... 14

1.9 HPLC Purification ... 14

1.10 Chiral HPLC Analysis ... 14

1.11 Remodelling Capping Loop ... 14

1.12 ECD ... 14

5. Supplementary Figures ... 15

3. Supplementary Tables ... 25

4. NMR Spectra ... 28

5. Supplementary References ... 43

1. Experimental Procedures

1.1 Gene Cloning

The DNA fragment corresponding to AbmU was PCR-amplified from commercially produced, codon optimised, synthetic DNA (Eurofins MWG) using primers AAGTTCTGTTTCAGGGCCCGATGAACGAACGGTTTACCTTACCGG (forward) and ATGGTCTAGAAAGCTTTATTACGCGGTACGTCCCGCA (reverse). The PCR product was ligated into vector pOPINF[1] using the In-Fusion™ system (Clontech). The resulting plasmid encoded N-terminally hexa-histidinetagged AbmU. The construct was verified by DNA sequencing.

1.2 Protein Expression and Purification

E. coli BL21 (DE3) cells harbouring AbmU expression plasmids were grown in LB medium supplemented with carbenicillin at 37 °C to OD_{600} = 0.4–0.6. Protein expression was induced by adding IPTG (1 mM) and incubating a culture for 16 h at 20 °C. Bacteria were harvested by centrifugation, suspended in buffer [50 mM Tris-HCl, 150 mM NaCl, 20 mM imidazole, pH 8], and lysed with a cell disruptor (Z Plus Series cell disruptor, Constant Systems Ltd.) at 25,000 p.s.i. Cell supernatant was applied to a 5-ml His-Trap HP chelating column (pre-loaded with nickel, GE Healthcare) and eluted with an imidazole gradient (20–500 mM) over 15 column volumes. Fractions containing AbmU protein were pooled, concentrated, and further purified by passage through a Hi-Load 16/60 Superdex 75 column (GE Healthcare) pre-equilibrated in buffer [20 mM Tris-HCl, 150 mM NaCl, pH 8]. Fractions containing AbmU protein were pooled and concentrated by ultrafiltration to 20 mg/ml.

1.3 Protein Crystallisation and Heavy Atom Soaking

Conditions supporting the growth of crystals of AbmU were initially identified using the sitting drop method of vapor diffusion at 20 °C and commercially available screens. Diffraction quality crystals of AbmU were grown in 0.2 M sodium acetate, 0.1 M sodium cacodylate, 32% PEG 8000, pH 6. Crystals selected for diffraction data collection were soaked in the well condition plus 1 M NaBr for 15 s,[2] then mounted in appropriately sized cryoloops (Hampton Research) and immediately flash-cooled in liquid nitrogen without additional cryoprotection prior to analysis.

1.4 Diffraction Data Collection and Structure Determination

Diffraction data were collected at Diamond Light Source, UK, on beamline I03. Data were auto-processed, merged and scaled with Xia2/DIALS[3,4] as implemented in the CCP4i2 suite.[5] The structure of AbmU was initially determined in space group $P12_11$, with two dimers of the molecule in the asymmetric unit, to 2.05 Å resolution, using the SAD method as applied to NaBr soaked crystals of AbmU. Identification of heavy atom sites, the resulting initial phase calculation and model building were carried out using CRANK2.[6,7] 7 bromide sites were located. Iterative rounds of manual model building and refinement using COOT[8] and Refmac5[9] were used to refine the structure. Data collection, phasing, and refinement statistics for AbmU are provided in Table S1. Protein structure graphics were prepared using UCSF Chimera (version 4), developed by the Resource for Biocomputing, Visualization, and Informatics at the University of California, San Francisco, with support from NIH P41-GM103311.[10]

1.5 Electrostatic Potential Calculations

For figures showing an electrostatic potential projected on the molecular surface of protein structures, the PoissonBoltzmann electrostatic potential on the solvent-accessible surface is shown, with potential values ranging from −10 kT/e (red) to 10 kT/e (blue). This was calculated using the APBS[11] and a PQR file generated using PDB2PQR[12] with PARSE force-field charges after protonation state assignment by PropKa.[13,14]

1.6 Chemical Synthesis

1.6.1 General Experimental Procedures

All reagents were obtained from commercial sources and were used as purchased. Where anhydrous conditions were necessary, reactions were carried out in flame-dried glassware under a positive pressure of nitrogen using standard Schlenk syringe-septa techniques. Anhydrous solvents were obtained from an Anhydrous Engineering Ltd. modified Grubbs system of double alumina and alumina-copper catalysed drying columns.[15] Triethylamine and diisopropylamine were distilled over CaH_2 under an atmosphere of nitrogen prior to use. Petroleum ether is of the 40 – 60 °C boiling point range. All stated temperatures below ambient are the temperatures of the cooling baths.

Routine monitoring of reactions was conducted by analytical TLC using aluminium sheets pre-coated with silica (Merck-Keiselgel 60 F_{254}) with a suitable solvent system and visualised using 254 nm UV light and/or developed with potassium permanganate solution and heat. Flash column chromatography was performed according to the procedure used by Still *et al.*[16] Normal phase HPLC chromatographic separations and purifications were performed on an Agilent 1260 Infinity system. Infra-red spectra were recorded on a Perkin Elmer Spectrum 100 FT-IR spectrometer with an ATR diamond cell and frequencies are reported in wavenumbers (cm^{-1}). Only strong and selected absorbances are reported. Mass spectrometry was performed by the University of Bristol mass spectrometry service by electrospray ionisation (ESI) or atmospheric pressure chemical ionisation (APCI) using a Bruker MicrOTOF II or Thermo Scientific Orbitrap Elite spectrometer. Optical rotations ($[\alpha]^T_D$) were recorded on a Bellingham and Stanley Ltd. ADP220 polarimeter and are quoted in (° ml)(g dm)$^{-1}$.

^{1}H and ^{13}C NMR spectra were recorded using JEOL ECS 400, JEOL ECZ 400, JEOL-VAR ECZ 400, Bruker Advance III HD 500 Cryo and Varian VNMR S500 spectrometers. NMR samples were analysed as solutions with the solvent specified at ambient temperature and referenced to residual solvent peaks. Chemical shifts (δ) are reported in parts per million (ppm) and coupling constants (*J*) are reported to the nearest 0.5 Hertz (Hz). Assignments were made with the aid of 2D NMR experiments.

1.6.2 Synthetic Compounds Ethyl (2*E*,4*E*)-4-methylhexa-2,4-dienoate 24

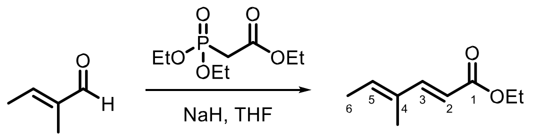

23 24

Under an atmosphere of nitrogen, NaH (60%, 694 mg, 28.9 mmol) was suspended in anhydrous THF (207 mL) and the suspension cooled to 0 °C. Triethyl phosphonoacetate (9.83 mL, 49.6 mmol) was added dropwise and the reaction was stirred at 0 °C for 30 mins. Tiglic aldehyde **23** (1.74 mL, 20.7 mmol) was added dropwise and the reaction was allowed to warm to room temperature and stirred overnight. The reaction was quenched with sat. aq. NH_4Cl (200 mL) and the layers were separated. The aqueous layer was extracted with EtOAc (2 × 150 mL). The combined organic extracts were dried ($MgSO_4$), filtered and concentrated *in vacuo*. The crude residue was purified by flash column chromatography, eluting with 5% EtOAc in petroleum ether to give ester **24** (2.93 g, 92%) as a colourless oil. δ_H (400 MHz, $CDCl_3$) 7.31 (1H, d, *J* 16.0, 3-H), 5.98 (1H, q, *J* 7.0, 5-H), 5.77 (1H, d, *J* 16.0, 2-H), 4.20 (2H, q, *J* 7.0, OC*H*$_2$CH$_3$), 1.81 (3H, d, *J* 7.0, 6-H$_3$), 1.76 (3H, s, 4-CH$_3$), 1.29 (3H, t, *J* 7.0, OCH$_2$C*H*$_3$). δ_C (101 MHz, $CDCl_3$) 167.8 (C-1), 149.6 (C-3), 136.5 (C-5), 133.9 (C-4), 115.4 (C-2), 60.3 (OCH$_2$), 14.7 (4-CH$_3$), 14.5 (OCH$_2$CH$_3$), 11.9 (C-6).

All data are in accordance with literature.[17]

(2*E*,4*E*)-4-Methylhexa-2,4-dien-1-ol 25

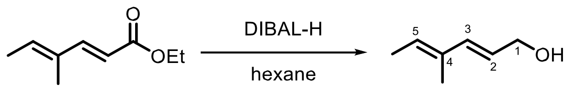

Under an atmosphere of nitrogen, ester **24** (4.45 g, 28.9 mmol) was dissolved in anhydrous hexane (144 mL) and the solution cooled to −78 °C. DIBAL-H (1 M, 63.5 mL, 63.5 mmol) was added dropwise and the reaction was stirred at −78 °C for 1 h. The reaction was quenched with sat. aq. Rochelle's salt (50 mL) and stirred vigorously overnight. The layers were separated, and the aqueous layer was extracted with Et_2O (2 × 100 mL). The combined organic extracts were dried ($MgSO_4$), filtered and concentrated *in vacuo*. The crude residue was purified by flash column chromatography, eluting with 20% EtOAc in petroleum ether to give alcohol **25** (3.31 g, 99%) as a colourless oil. $\boldsymbol{\delta_H}$ (400 MHz, $CDCl_3$) 6.25 (1H, d, *J* 16.0, 3-H), 5.71 (1H, dt, *J* 16.0, 6.0, 2-H), 5.57 (1H, q, *J* 7.0, 5-H), 4.19 (2H, d, *J* 6.0, 1-H_2), 1.74 (3H, s, 4-CH_3), 1.73 (3H, d, *J* 7.0, 6-H_3). $\boldsymbol{\delta_C}$ (101 MHz, $CDCl_3$) 136.9 (C-3), 134.0 (C-4), 127.7 (C5), 124.9 (C-2), 64.1 (C-1), 14.0 (C-6), 12.2 (4-CH_3).

All data are in accordance with literature.[18]

(2*E*,4*E*)-4-Methylhexa-2,4-dienal 11

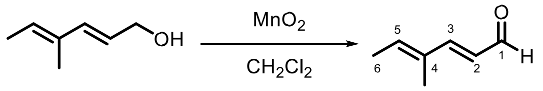

Under an atmosphere of nitrogen, alcohol **25** (720 mg, 6.42 mmol) was dissolved in CH_2Cl_2 (32 mL). Activated manganese oxide (3.88 g, 44.6 mmol) was added and the reaction was stirred until complete consumption of the starting material was observed by TLC (45 mins). The solution was filtered through a plug of Celite® and washed with CH_2Cl_2 (60 mL). The solvent was removed *in vacuo* to give aldehyde **11** (721 mg, quant.) as a pale-yellow oil. The crude residue was used directly in the next step without further purification. $\boldsymbol{\delta_H}$ (400 MHz, $CDCl_3$) 9.55 (1H, d, *J* 7.5, 1-H), 7.11 (1H, d, *J* 15.5, 3-H), 6.16-6.03 (2H, m, 2-H, 5-H), 1.86 (3H, d, *J* 7.5, 6-H_3), 1.81 (3H, s, 4-CH_3). $\boldsymbol{\delta_C}$ (101 MHz, $CDCl_3$) 194.4 (C-1), 157.9 (C-3), 139.1 (C-5), 134.6 (C-4), 126.7 (C-2), 15.0 (C-6), 12.1 (4-CH_3).

All data are in accordance with literature.[19]

(*R*)-5-Benzyl-*N*-(propionyl)oxazolidin-2-one 7

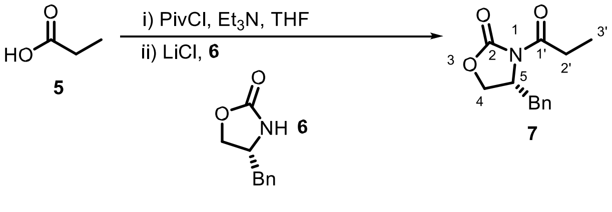

Under an atmosphere of nitrogen, of propionic acid **5** (2.50 g, 33.7 mmol) and Et_3N (10.4 mL, 74.2 mmol) were dissolved in anhydrous THF (135 mL) and the solution cooled to −20 °C. Pivaloyl chloride (4.98 mL, 40.5 mmol) was added dropwise and the mixture was stirred for 0.5 h at −20 °C after which a cloudy white precipitate formed. (*R*)-oxazolidinone **6** (6.57 g, 37.1 mmol) and anhydrous LiCl (2.86 g, 67.5 mmol) were added sequentially at −20 °C, and the mixture was then allowed to warm to room temperature and stirred for 24 h. The reaction was quenched with sat. aq. $NaHCO_3$ (135 mL) and the organic solvent was removed *in vacuo*. The aqueous layer was extracted with Et_2O (3 × 150 mL), and the combined organic layers were washed with sat. aq. $NaHCO_3$ (80 mL) and brine (2 × 150 mL), dried ($MgSO_4$) and concentrated *in vacuo*. The crude residue was purified by flash column chromatography eluting with 30-60% Et_2O in petroleum ether to give (*S*)-oxazolidinone **7** (7.32 g, quant.) as a paleyellow oil. $[\alpha]^{24}_{D}$ −52.0 (*c* 1.0, $CHCl_3$) Lit.[17] $[\alpha]^{20}_{D}$ −65.1 (*c* 1.0, $CHCl_3$). δ_H (400 MHz, $CDCl_3$) 7.36 – 7.19 (5H, m, ArCH), 4.67 (1H, ddt, *J* 9.5, 7.5, 3.5, 5-H), 4.23 – 4.15 (2H, m, 4-H_2), 3.31 (1H, dd, *J* 13.5, 3.5, 5-C*H*HPh), 3.04 – 2.88 (2H, m, 2′-H_2), 2.77 (dd, *J* 13.5, 9.5, 5-CH*H*Ph), 1.21 (3H, t, *J* 7.5, 3′-H_3). δ_C (101 MHz, $CDCl_3$) 174.1 (C-1′), 153.5 (C-2), 135.4 (ArC), 129.4 (ArCH), 129.0 (ArCH), 127.4 (ArCH), 66.2 (C-4), 55.2 (C-5), 38.0 (5-CH_2Ph), 29.2 (C-2′), 8.3 (C-3′).

All data are in accordance with literature.[20]

(*R*)-5-Benzyl-*N*-(*S*)-2′-methylpent-4′-enoyloxazolidin-2-one 8

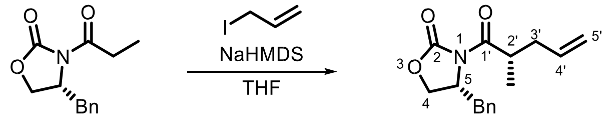

Under an atmosphere of nitrogen, oxazolidinone **7** (7.67 g, 32.9 mmol) was dissolved in anhydrous THF (90 mL) and the solution cooled to −78 °C. NaHMDS (1 M in THF, 39.5 mL, 39.5 mmol) was added dropwise and the reaction was stirred at −78 °C for 1 h. Allyl iodide (4.51 mL, 49.3 mmol) was then added dropwise at −78 °C and the reaction mixture was stirred for 2 h at the same temperature. The reaction was quenched with sat. aq. NH_4Cl (90 mL) and the organic solvent was removed *in vacuo*. The aqueous layer was extracted with Et_2O (3 × 100 mL). The combined organic extracts were washed with brine (40 mL), dried ($MgSO_4$) and concentrated *in vacuo*. The crude residue was purified by flash column chromatography eluting with 10% Et_2O in petroleum ether to give oxazolidinone **8** (8.49 g, 94%, *dr* = 95:5) as a pale-yellow oil. $[\alpha]^{21}_{D}$ −34.0 (*c* 1.0, $CHCl_3$) Lit.[18] $[\alpha]^{19}_{D}$ −39.0 (*c* 1.0 $CHCl_3$). δ_H (400 MHz, $CDCl_3$) 7.36 – 7.20 (5H, m, ArCH), 5.83 (1H, ddt, *J* 17.0, 10.0, 7.0, 4′-H), 5.11 (1H, dq, *J* 17.0, 1.5, 5′-*H*H), 5.08 – 5.02 (1H, m, 5′-H*H*), 4.68 (1H, ddt, *J* 10.0, 7.0, 3.5, 5-H), 4.21 – 4.14 (2H, m, 4-H_2), 3.87 (1H, sext., *J* 7.0, 2′-H), 3.29 (1H, dd, *J* 13.5, 3.5, 5-C*H*HPh), 2.70 (1H, dd, *J* 13.5, 10.0, 5-CH*H*Ph), 2.53 (1H, dtt, *J* 14.0, 7.0, 1.5, 3′-*H*H), 2.24 (1H, dtt, *J* 14.0, 7.0, 1.0 Hz, 3′-H*H*), 1.19 (3H, d, *J* 7.0, 2′-CH_3). δ_C (101 MHz, $CDCl_3$) 176.7 (C-1′), 153.3 (C-2), 135.5 (ArC), 135.4 (C-4′), 129.6 (ArCH), 129.1 (ArCH), 127.5 (ArCH), 117.4 (C-5′), 66.1 (C-4), 55.5 (C-5), 38.2 (5-CH_2Ph), 38.1 (C-3), 37.3 (C-2′), 16.6 (2′-CH_3).

All data are in accordance with literature.[21]

(*R*)-5-Benzyl-*N*-(*S*)-5′-hydroxy-2′-methylpentanoyloxazolidin-2-one 9

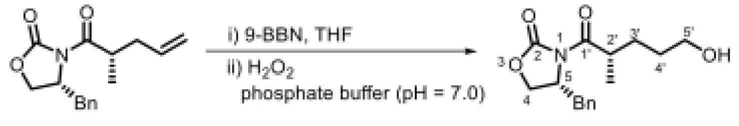

8 9

Under an atmosphere of nitrogen, oxazolidinone **8** (2.54 g, 9.28 mmol) was dissolved in anhydrous THF (24 mL) and the solution was cooled to 0 °C. 9-BBN (0.5 M in THF, 28 mL, 13.9 mmol) was added dropwise, and the mixture was allowed to warm to room temperature. After 1.5 h the reaction was cooled to 0 °C and quenched with aq. phosphate buffer (26 mL, pH 7.0) and aq. H_2O_2 (26 mL, 30% w/w). The resulting mixture was stirred at room temperature for 2 h before it was diluted with H_2O (50 mL). The layers were separated, and the aqueous layer was extracted with EtOAc (3 × 150 mL). The combined organic extracts were washed with sat. aq. $Na_2S_2O_3$ (50 mL) and brine (50 mL), dried ($MgSO_4$) and concentrated *in vacuo*. The crude residue was purified by flash column chromatography eluting with 40% EtOAc in petroleum ether to give oxazolidinone **9** (2.41 g, 89%). $[\alpha]^{21}_D$ −28.0 (*c* 1.0 $CHCl_3$). **δ_H** (500 MHz, $CDCl_3$) 7.36 – 7.21 (5H, m ArCH), 4.72 – 4.66 (1H, m, 5-H), 4.23 – 4.16 (2H, m, 4′-H_2), 3.74 (1H, sext., *J* 7.0, 2′-H), 3.68 (2H, t, *J* 6.0, 5′-H_2), 3.31 (1H, dd, *J* 13.5, 3.5, 5-C*H*HPh), 2.73 (1H, dd, *J* 13.5, 10.0, 5-CH*H*Ph), 1.91 (1H, m, 3′-*H*H), 1.75 – 1.46 (4H, 3′-H*H*, 4′-H_2, OH), 1.19 (3H, d, *J* 7.0, 2′-CH_3). **δ_C** (126 MHz, $CDCl_3$) 177.2 (C-1′), 153.4 (C-2), 135.4 (ArC), 129.6 (ArCH), 129.1 (ArCH), 127.5 (ArCH), 66.3 (C-4), 62.2 (C-5’), 55.6 (C-5), 38.2 (5-CH_2Ph), 37.1 (C-2′), 30.0 (C-3′), 29.8 (C-4′), 16.5 (2′-CH_3). **IR** (ν_{max}/cm^{-1}) 3501 (br), 2932, 1778, 1697, 1388, 1212. **HRMS** (ESI) calculated for $[C_{16}H_{21}NO_4+Na]^+$ 314.1363, found 314.1355.

(*S*)-3-Methyltetrahydro-2*H*-pyran-2-one 10

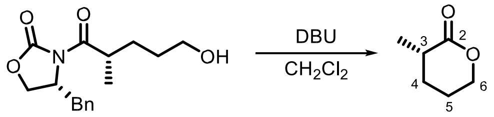

9 10

Alcohol **9** (400 mg, 1.35 mmol) was dissolved in CH_2Cl_2 (13.5 mL). DBU (40 µL, 0.27 mmol) was added and the mixture was stirred at room temperature for 0.5 h. The solution was diluted with H_2O (5 mL) and the resulting layers were separated. The aqueous layer was extracted with Et_2O (3 × 10 mL) and the combined organic extracts were washed with brine (5 mL), dried ($MgSO_4$), filtered and concentrated *in vacuo*. The crude residue was purified by flash column chromatography eluting with 60% Et_2O in petroleum ether to give lactone **10** (131 mg, 85%) as a colourless oil. $[\alpha]^{21}_D$ +53.8 (*c* 1.3 $CHCl_3$). Lit.[19] $[\alpha]^{25}_D$ +67.3 (*c* 6.6 MeOH). **δ_H** (400 MHz, $CDCl_3$) 4.36 – 4.26 (2H, m, 6-H_2), 2.57 (1H, dp, *J* 11.0, 7.0, 3-H), 2.09 (1H, m, 4-*H*H), 1.97 – 1.83 (2H, m, 5-H_2), 1.54 (1H, ddt, *J* 13.5, 11.0, 7.5, 4-H*H*), 1.25 (3H, d, *J* 7.0, 3-CH_3. **δ_C** (101 MHz, $CDCl_3$) 175.4 (C-2), 68.7 (C-6), 34.8 (C-3), 27.3 (C-4), 22.2 (C5), 16.8 (3-CH_3).

All data are in accordance with literature.[19]

(4*S*,6*E*,8*E*,10*E*)-1-Hydroxy-4,10-dimethyldodeca-6,8,10-trien-5-one 12

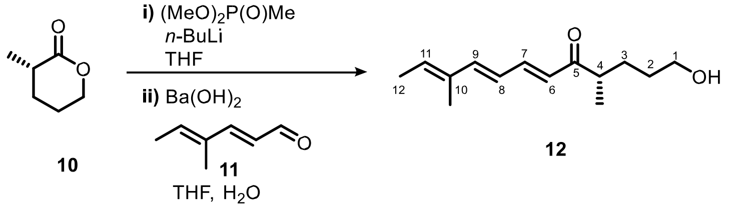

Under an atmosphere of nitrogen, dimethyl methylphosphonate (1.41 mL, 13.0 mmol) was dissolved in anhydrous THF (186 mL) and the solution cooled to −78 °C. *n*-BuLi (2.5 M in hexanes, 4.96 mL, 12.4 mmol) was added dropwise and the mixture was stirred for 1 h at −78 °C. Lactone **10** (674 mg, 5.91 mmol) in THF (12.6 mL) was added dropwise and the mixture was stirred for an additional 1 h at −78 °C. The reaction was quenched by addition of sat. aq. NH4Cl (50 mL), the layers were separated and the organic layer extracted with EtOAc (5 × 150 mL). The combined organic extracts were dried ($MgSO_4$), filtered and concentrated *in vacuo* to give crude phosphonate which was used directly in the next step. The crude residue was dissolved in THF (45 mL) and the solution was cooled to 0 °C. $Ba(OH)_2$ (1.52 g, 8.86 mmol) and aldehyde **11** (781 mg, 7.09 mmol) in THF (11 mL) were added sequentially. H2O (2.2 mL) was then added dropwise, and the mixture was allowed to warm to room temperature and stirred overnight. The reaction was quenched by the addition of sat. aq. NH_4Cl (20 mL) and the layers were separated. The aqueous layer was extracted with EtOAc (3 × 50 mL) and the combined organic extracts were dried (Na_2SO_4), filtered, and concentrated *in vacuo*. The crude residue was purified by flash column chromatography (silica pre-washed with 0.1% Et_3N) eluting with 50% EtOAc in petroleum ether to give alcohol **12** (1.05 g, 80%) as a bright yellow oil. $[\alpha]^{21}_D$ +5.7 (*c* 1.0, MeOH). **δ_H** (500 MHz, CD_3OD) 7.35 (1H, dd, *J* 15.5, 11.0, 7-H), 6.74 (1H, d, *J* 15.5, 9-H), 6.37 (1H, dd, *J* 15.5, 11.0, 8-H), 6.32 (1H, d, *J* 15.5, 6-H), 5.88 (1H, q, *J* 6.5, 11-H), 3.54 – 3.51 (2H, m, 1-H_2), 2.88 (1H, m, 4-H), 1.81 (3H, s, 10-CH_3), 1.81 (3H, d, *J* 6.5, 12-H_3), 1.77-1.64 (2H, m, 3-H_2), 1.54-1.42 (2H, m, 2-H_2), 1.10 (3H, d, *J* 7.0, 4-CH_3). **δ_C** (126 MHz, CD_3OD) 206.8 (C-5), 148.6 (C-9), 145.9 (C-7), 136.3 (C-10), 134.2 (C-11), 128.1 (C-6), 125.3 (C-8), 62.8 (C-1), 44.8 (C-4), 31.3 (C-2), 30.9 (C-3), 17.3 (4-CH_3), 14.4 (C-12), 11.9 (10-CH_3). **IR** (ν_{max}/cm^{-1}) 3405, 2962, 2935,1669, 1647, 1595, 1579. **HRMS** (ESI) calculated for $[C_{14}H_{22}O_2+Na]^+$ 245.1512 found 245.1505.

(6*E*,8*E*,10*E*)-4,10-Dimethyl-5-oxododeca-6,8,10-trienal 13

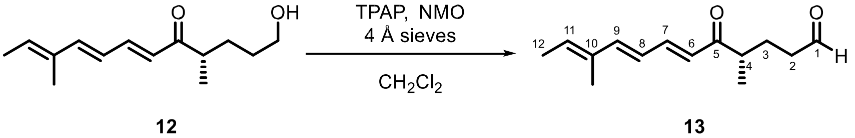

Under an atmosphere of nitrogen, alcohol **12** (339 mg, 1.52 mmol) was dissolved in CH_2Cl_2 (15 mL). Powdered 4 Å molecular sieves (760 mg) and solid NMO (268 mg, 2.29 mmol) were added, and the solution was stirred at room temperature for 15 mins. TPAP (55 mg, 0.152 mmol) was added to the solution and the reaction was stirred for 1 h. The mixture was concentrated *in vacuo* to approx. 2 mL and directly loaded onto a silica column and purified by flash column chromatography, eluting with 30% EtOAc in petroleum ether to give aldehyde **13** (266 mg, 80%) as a bright yellow oil. $[\alpha]^{21}_D$ +12.0 (*c* 0.5, $CHCl_3$). **δ_H** (500 MHz, $CDCl_3$) 9.75 (1H, t, *J* 1.5, CHO), 7.29 (1H, dd, *J* 15.0, 11.0, 7-H), 6.65 (1H, d, *J* 15.0, 9-H), 6.27- 6.19 (2H, m, 6-H, 8-H), 5.83 (1H, q, *J* 7.0, 11-H), 2.81 (2H, sext., *J* 7.0, 4-H), 2.49-2.40 (2H, m, 2-H_2), 2.01 (1H, m, 3-*H*H), 1.80 (3H, d, *J* 7.0, 12-H_3), 1.79 (3H, s, 10-CH_3) 1.74-1.69 (1H, m, 3-H*H*), 1.13 (3H, d, *J* 7.0, 4-H_3). **δ_C** (126 MHz, $CDCl_3$) 203.1 (C-5), 202.1 (C-1), 147.5 (C-9), 144.3 (C-7), 135.0 (C-10), 133.6 (C-11), 126.9 (C-6), 124.0 (C-8), 43.3 (C-4), 41.7 (C-2), 25.3 (C-3), 17.2 (4-CH_3), 14.6 (C-12), 12.0

(10-CH3). **IR** (v_{max}/cm^{-1}) 2928, 1723, 1678, 1654, 1580. **HRMS** (ESI) calculated for $[C_{14}H_{20}O_2+H]^+$ 221.1536 found 221.1540.

(6*E*,8*E*,10*E*)-4,10-Dimethyl-1-hydroxy-1-(5'-methoxy-4-methylene-2'-oxo-2',4'-dihydrofuran-1'yl)-dodeca6,8,10-en-5-one 15

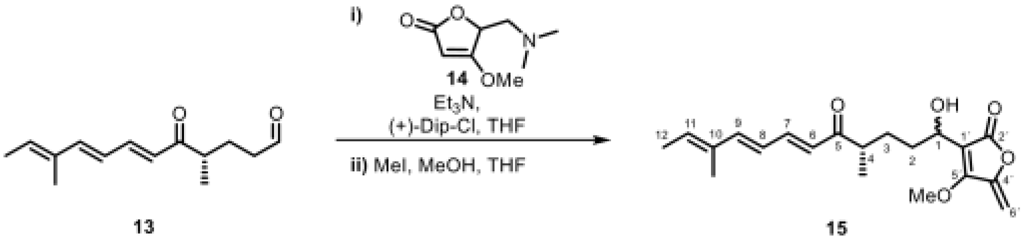

Under an atmosphere of nitrogen, tetronate **14**[20] (156 mg, 0.915 mmol) was dissolved in anhydrous THF (11 mL) and the solution cooled to –78 °C. Anhydrous triethylamine (0.24 mL, 1.74 mmol) followed by (+)-DIP-Cl (0.78 M in hexanes, 1.12 mL, 0.871 mmol) were added dropwise and the reaction mixture was stirred for 30 mins. A solution of aldehyde **13** (96 mg, 0.436 mmol) in THF (2 mL) was added dropwise and the reaction was stirred for 1 h at –78 °C. The temperature was raised to 0 °C, then MeOH (0.5 mL) and MeI (0.27 mL, 4.36 mmol) were added sequentially and stirred for 3 h. The reaction was quenched with sat. aq. $NaHCO_3$ (5 mL) and the mixture stirred at room temperature for 1 h. The mixture was separated, and the aqueous layer extracted with EtOAc (3 × 10 mL). The combined organic extracts were dried over ($MgSO_4$), filtered and concentrated *in vacuo*. The crude residue was purified by flash column chromatography, eluting with 15–20% EtOAc in petroleum ether to give alcohol **15** (87 mg, 47%) as a 1:1 mixture of diastereomers. **IR** (v_{max}/cm^{-1}) 3437, 2928, 1766, 1625, 1455. **HRMS** (ESI) calculated for $[C_{20}H_{26}O_5+Na]$ 369.1672 found 369.1661.

(6*E*,8*E*,10*E*)-4,10-Dimethyl-1-(5'-methoxy-4-methylene-2'-oxo-2',4'-dihydrofuran-1'yl)-dodeca-6,8,10-ene1,5-dione 16

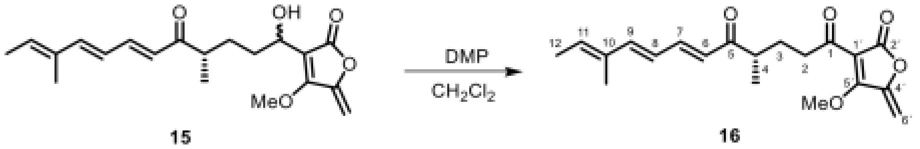

Dess-Martin periodinane (76 mg, 0.219 mmol) was added to a solution of alcohol **15** (100 mg, 0.241 mmol) in CH_2Cl_2 (9 mL) and the reaction mixture as stirred at room temperature for 1 h. The reaction was quenched with a 1:1 mixture of sat. aq. $Na_2S_2O_3$ (5 mL) and sat. aq. $NaHCO_3$ (5 mL) and the resulting mixture was stirred vigorously for 30 mins. The layers were separated, and the organic layer was extracted with CH_2Cl_2 (2 × 10 mL). The combined organic extracts were dried over ($MgSO_4$), filtered and concentrated *in vacuo*. The crude residue was purified by flash column chromatography, eluting with 20% EtOAc in petroleum ether and concentrated under a stream of nitrogen gas to give diketone **16** (69 mg, 90%) as a yellow oil. $[\alpha]^{21}_D$ −23.0 (*c* 0.1, $CHCl_3$). **δ_H** (500 MHz, $CDCl_3$) 7.28 (1H, dd, *J* 15.5, 11.0, 7-H), 6.64 (1H, d, *J* 15.0, 9-H), 6.30-6.22 (2H, m, 6-H, 8-H), 5.82 (1H, q, *J* 7.0, 11-H), 5.27 (1H, d, *J* 3.0, 6-*H*H), 5.21 (1H, d, *J* 3.0, 6′-H*H*), 4.15 (3H, s, OCH_3), 3.02-2.94 (2H, m, 2-H_2), 2.82 (1H, m, 4-H), 2.03 (1H, m, 3-*H*H), 1.80 (3H, d, *J* 7.0, 12-H_3), 1.79 (3H, s, 10-CH_3), 1.73 (1H, s, 3-H*H*), 1.15 (3H, d, *J* 7.0, 4-CH_3). **δ_C** (126 MHz, $CDCl_3$) 203.3 (C-5), 196.5 (C-1), 168.1 (C-5), 166.6 (C-2), 149.1 (C-4), 147.3 (C-9), 144.1 (C-

7), 135.0 (C-10), 133.4 (C-11), 126.9 (C-8), 124.1 (C-6), 105.3 (C-1), 96.1 (C-6), 63.4 (OCH_3), 43.6 (C-4), 40.6 (C2), 27.0 (C-3), 17.2 (4-CH_3), 14.6 (C-12), 12.0 (10-CH_3). **IR** (v_{max}/cm^{-1}) 2928, 1769, 1653, 1601. **HRMS** (ESI) calculated for $[C_{20}H_{24}O_5+H]^+$ 345.1697 found 345.1683.

Thermal Diels-Alder Reaction

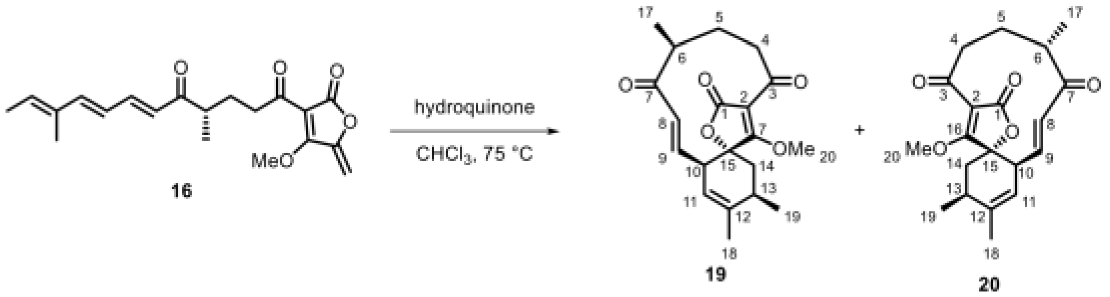

Tetronate **16** (8.0 mg, 23.2 µmol) and hydroquinone (0.25 mg, 2.32 µmol) were dissolved in $CHCl_3$ (1.5 mL) and the solution was heated at 75 ºC in a sealed tube until HPLC analysis showed complete consumption of the linear starting material (16 h). The reaction mixture was cooled to room temperature and concentrated *in vacuo*. A portion of the crude material (6 mg) was purified by HPLC to give Diels-Alder adducts **19** (3.5 mg, 44%) and **20** (1.2 mg, 15%). The structures of the products were elucidated by extensive NMR analysis (^{1}H, ^{13}C, COSY, HSQC, HMBC, NOESY, See Table S2 and Table S3).

Major diastereomer **19**: **δ_H** (500 MHz, $CDCl_3$) 6.44 (1H, d, *J* 16.5, 8-H), 6.38 (1H, dd, *J* 16.5, 7.0, 9-H), 5.38 (1H, m, 11-H), 3.88 (3H, s, OCH_3), 3.47 (1H, m, 10-H), 2.97 (1H, ddd, *J* 13.0, 8.0, 4.0, 4-*H*H), 2.89 (1H, pd, *J* 6.5, 4.0. 6-H), 2.48 (1H, m, 13-H), 2.38 (1H, m, 4-H*H*), 2.33 (1H, dd, *J* 14.5, 7.0, 14-*H*H), 2.02 (1H, m, 5-*H*H), 1.84-1.80 (2H, m, 5-H*H*, 14-H*H*), 1.79 (3H, s, 12-CH_3), 1.16 (3H, d, *J* 7.0, 13-CH_3), 1.11 (3H, d, *J* 6.5. 6-CH_3). **δ_C** NMR (126 MHz, $CDCl_3$) 204.6 (C-7), 198.2 (C-3), 179.4 (C-16), 169.5 (C-1), 142.2 (C-12), 142.1 (C-9), 132.8 (C-8), 118.4 (C-11), 106.8 (C-2), 86.0 (C-15), 61.9 (OCH_3), 45.8 (C-10), 45.2 (C-6), 43.0 (C-4), 37.8 (C-14), 32.2 (C-13), 29.9 (C-5), 21.0 (12-CH_3), 18.8 (13-CH_3), 16.1 (6-CH_3). **IR** (v_{max}/cm^{-1}) 2967, 2924, 2854, 1752, 1691, 1629, 1451. **HRMS** (ESI) calculated for $[C_{20}H_{24}O_5+H]^+$ 345.1697, found 345.1708.

Minor diastereomer **20**: **δ_H** (500 MHz, $CDCl_3$) 6.22 (1H, d, *J* 16.5, 8-H), 5.98 (1H, dd, *J* 16.5, 10.5. 9-H), 5.27 (1H, q, *J* 2.0. 11-H), 4.13 (3H, s, OCH_3), 3.33 (1H, dq, *J* 10.5, 2.0, 10-H), 3.03 (1H, ddd, *J* 15.5, 8.0, 4.0, 4-*H*H), 2.82 (1H, sext., *J* 6.5, 6-H), 2.50 (1H, m, 13-H), 2.43 (1H, ddd, *J* 15.5, 10.0, 4.0, 4-H*H*), 2.37 (1H, dd, *J* 14.5, 7.0, 14*H*H), 2.13 (1H, dddd, *J* 15.5, 10.0, 6.5, 4.0, 5-*H*H), 1.86 (1H, dd, *J* 14.5, 5.0, 14-H*H*), 1.78 (3H, s, 12-CH_3), 1.64 (1 H, dddd, *J* 15.5, 8.0, 6.5, 4.0, 5-H*H*), 1.18 (3H, d, *J* 7.5, 13-CH_3), 1.10 (3H, d, *J* 6.5, 6-CH_3). **δ_C** (126 MHz, $CDCl_3$) δ 204.8 (C-7), 198.8 (C-3), 178.7 (C-16), 169.8 (C-1), 141.9 (C-12), 141.7 (C-9), 133.5 (C-8), 118.1 (C-11), 108.6 (C-2), 85.9 (C-15), 63.4 (OCH_3), 48.2 (C-10), 43.5 (C-6), 41.4 (C-4), 37.0 (C-14), 32.5 (C-13), 31.0 (C-5), 21.0 (12CH_3), 19.0 (13-CH_3), 15.6 (6-CH_3). **IR** (v_{max}/cm^{-1}) 2960, 2923, 2857, 1751, 1887, 1609, 1455. **HRMS** (ESI) calculated for $[C_{20}H_{24}O_5+H]^+$ 345.1697, found 345.1683.

AbyU-Type Thermal Diels-Alder Adduct 18

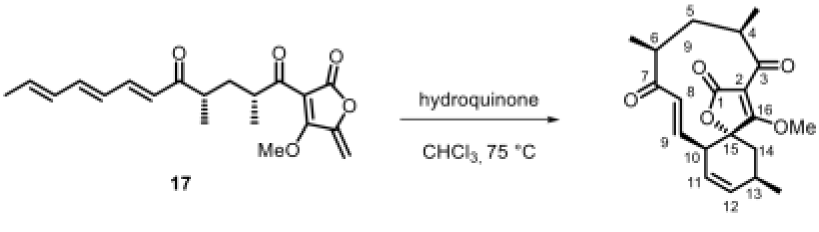

Tetronate **17** was prepared as described previously.[20] **17** (10 mg, 0.029 mmol) and hydroquinone (0.1 mg) were dissolved in $CHCl_3$ and the solution heated in a sealed tube at 75 °C for 48 h. The reaction mixture was cooled to room temperature and concentrated in vacuo. The crude residue was purified by flash column chromatography, eluting with 10-20% EtOAc in petroleum ether to give **18** as a colourless oil (6.4 mg, 64%). δ_H (500 MHz, $CDCl_3$) 6.47 (1H, dd, *J* 16.5, 6.0, 9-H), 6.25 (1H, d, *J* 16.5, 8-H), 5.86 (1H, dt, *J* 10.0, 3.0, 12-H), 5.68 (1H, dt, *J* 10.0, 3.0, 11-H), 3.91 (3H, s, OCH_3), 3.45 (1H, m, 10-H), 3.12 (1H, m, 4-H), 2.95 (1H, m, 6-H), 2.64 (1H, m, 13-H), 2.40 (1H, dd, *J* 14.5, 8.0, 14-*H*H), 1.87 (1H, ddd, *J* 15.5, 6.0, 4.0, 5-*H*H), 1.82 (1H, dd, *J* 14.5, 4.5, 14-H*H*), 1.21 (3H, d, *J* 7.0, 6-CH_3), 1.19 (3H, d, *J* 7.0, 4-CH_3), 1.17–1.13 (1H, overlapping m, 5-*H*H), 1.15 (3H, d, *J* 7.5, 13-CH_3). δ_C (126 MHz, $CDCl_3$) 204.3 (C-7), 200.6 (C-3), 178.2 (C-16), 169.9 (C-1), 141.5 (C-9), 136.7 (C-12), 131.6 (C-8), 121.8 (C-11), 107.0 (C-2), 86.1 (C-15), 61.7 (OCH_3), 46.65 (C-4), 46.6 (C-6), 44.6 (C-10), 39.0 (C-5), 36.6 (C-14), 29.3 (C-13) , 21.1 (13-CH_3), 17.0 (6-CH_3), 16.6 (4-CH_3).

All data are in accordance with the literature.[23]

(6*E*,8*E*,10*E*)-1-Hydroxydodeca-6,8,10-trien-5-one 28

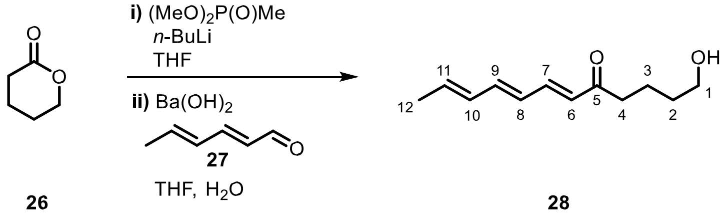

Under an atmosphere of nitrogen, dimethyl methylphosphonate (2.73 mL, 25.2 mmol) was dissolved in anhydrous THF (70 mL) and cooled to –78 °C. *n*-BuLi (1.52 M, 16.5 mL, 25.2 mmol) was added dropwise, and the mixture was stirred at –78 °C for 0.5 h. δ-Valerolactone **26** (1.20 g, 12.0 mmol) in anhydrous THF (30 mL) was added dropwise, and the reaction was stirred at –78 °C for 1 h. Sat. aq. NH_4Cl (60 mL) was added, and the aqueous layer was extracted with EtOAc (5 × 30 mL). The combined organic layers were dried (Na_2SO_4), filtered and the solvent removed *in vacuo*. The residue was dissolved in THF (100 mL) and cooled to 0 °C. Barium hydroxide (3.08 g, 18.0 mmol) and (2*E,4E*)-hexa-2,4-dienal **27** (1.98 mL, 18.0 mmol) were added. H_2O (30 mL) was then added dropwise, and then the reaction was stirred at room temperature for 16 h. Sat. aq. NH_4Cl (20 mL) was added, and then the aqueous layer was extracted with EtOAc (3 × 30 mL). The combined organic layers were dried (Na_2SO_4), filtered and the solvent removed *in vacuo*. The crude residue was purified by flash column chromatography, eluting with 60% EtOAc with 0.05% NEt_3 in petroleum ether (40-60 °C), giving alcohol **28** as a yellow solid (1.68 g, 72% over two steps). ^1H NMR showed a 10,11 *E*:*Z* ratio of 5:1. δ_H (400 MHz, CD_3OD) 7.29 (1H, dd, *J* 15.5, 11.0, 7-H), 6.69 (1H, dd, *J* 15.0, 10.5, 9-H), 6.41 – 6.14 (3H, m, 6-H, 8-H and 10-H), 6.02 (1H, dq, *J* 14.0, 7.0, 11-H), 3.56 (2H, t, *J* 6.5, 1-H_2), 2.64 (2H, t, *J* 7.3, 4-H_2), 1.83 (3H, d, *J* 7.0, 12-H_3), 1.66 (2H, m, 3-H_2), 1.55 (2H, m, 2-H_2). δ_C (101 MHz, CD_3OD) 203.4 (C-5), 145.3 (C-7), 143.9 (C-9), 136.6 (C-11), 132.7 (C-10), 129.4 (C-6 and C-8), 62.6 (C-1), 40.8

(C-4), 33.1 (C-2), 22.0 (C-3), 18.7 (C-12). **IR** (v_{max}/cm^{-1}) 3329, 2942, 2876, 1682, 1602, 1578. **MS** (ESI) calc. for [$C_{12}H_{18}O_2$+H]$^+$ 195.1, found 195.1.

(6*E*,8*E*,10*E*)-5-Oxododeca-6,8,10-trienal 29

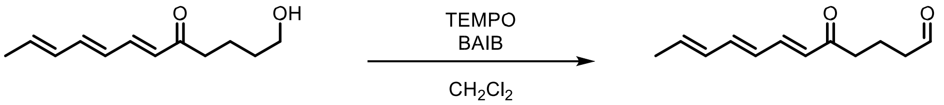

28 **29**

Under an atmosphere of nitrogen, alcohol **28** (1.68 g, 8.65 mmol) was dissolved in CH_2Cl_2 (60 mL) and cooled to 0 °C. (diacetoxyiodo)Benzene (6.69 g, 20.76 mmol) and TEMPO (541 mg, 3.46 mmol) were added, and the solution was stirred at room temperature for 3 h. The reaction was quenched by the addition of sat. aq. $Na_2S_2O_3$ (20 mL) and sat. aq. $NaHCO_3$ (4 mL) and the resultant biphasic mixture was stirred for a further 0.5 h. The layers were separated, and the aqueous layer was extracted with CH_2Cl_2 (3 × 50 mL). The combined organic layers were washed with brine (20 mL), dried ($MgSO_4$), filtered and the solvent removed *in vacuo.* The crude residue was purified by flash column chromatography, eluting with 30–60% EtOAc in petroleum ether (40-60 °C) to give **29** as a yellow oil (1.50 g, 90% yield). ^{1}H NMR showed a 10,11 *E*:*Z* ratio of 5:1. **δ_H** (400 MHz, $CDCl_3$) 9.78 (1H, s, 1-H), 7.19 (1H, dd, *J* 15.5, 11.0, 7-H), 6.58 (1H, dd, *J* 15.0, 10.5, 9-H), 6.24 – 6.09 (3H, m, 6-H, 8-H and 10-H), 5.98 (1H, dq, *J* 15.0, 7.0, 11-H), 2.62 (2H, t, *J* 7.0, 4-H_2), 2.52 (2H, t, *J* 7.0, 2-H_2), 1.96 (2H, m, 3-H_2), 1.84 (3H, d, *J* 7.0, 12H_3). **δ_C** (101 MHz, $CDCl_3$) 202.0 (C-1), 199.4 (C-5), 143.1 (C-7), 142.2 (C-9), 135.7 (C-11), 131.2 (C-10), 128.2 (C8), 128.2 (C-6), 43.0 (C-2), 39.0 (C-4), 18.5 (C-18), 16.5 (C-3). **IR** (v_{max}/cm^{-1}) 2939, 2887, 2732, 1714, 1672, 1601. **MS** (ESI) calc. for [$C_{12}H_{16}O_2$+H]$^+$ 193.1, found 193.1.

Achiral AbyU Substrate 21

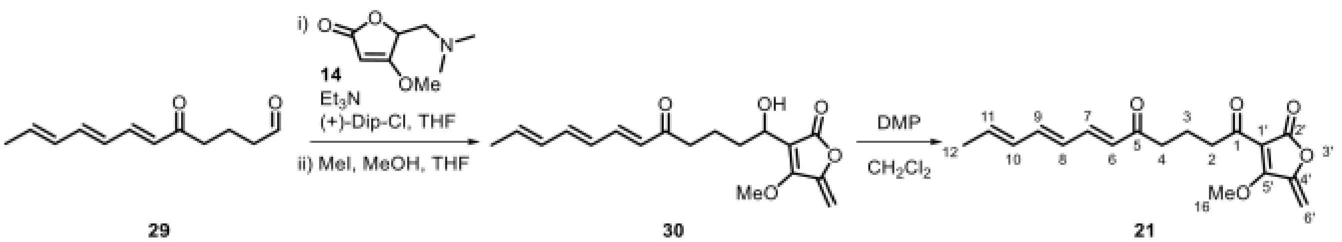

29 **30** **21**

Under an atmosphere of nitrogen, tetronate **14**[20] (430 mg, 2.50 mmol) was dissolved in anhydrous THF (15 mL). The solution was cooled to –78 °C and NEt_3 (663 µL, 4.76 mmol) and (+)-DIP-Cl (0.75 M solution in hexane, 3.17 mL, 2.38 mmol) were added dropwise. The solution was stirred for 0.5 h at –78 °C and then aldehyde **29** (231 mg, 1.19 mmol) in anhydrous THF (2 mL) was added dropwise and the solution stirred for a further 0.5 h at –78 °C. The reaction mixture was warmed to 0 °C and MeOH (1.37 mL) and iodomethane (0.74 mL, 11.90 mmol) were added and stirring continued at 0 °C for a further 3 h. The reaction was quenched by the addition of sat. aq. $NaHCO_3$ (10 mL) and then stirred at room temperature for a further 1 h. The layers were separated, and the aqueous layer was extracted with EtOAc (3 × 20 mL). The combined organic layers were dried ($MgSO_4$), filtered and the solvent removed *in vacuo*. The crude residue was purified by flash column chromatography, eluting with 30-50% EtOAc in petroleum ether (40-60 °C) to give a mixture of epimeric alcohols **30** (169 mg, 45%). The alcohol product (158 mg, 0.496 mmol) was dissolved in CH_2Cl_2 (5 mL) and the solution cooled to 0 °C. DMP (336 mg, 0.794 mmol) was added and the solution was allowed to warm to room temperature and stirred for 1 h. The reaction quenched by the addition of sat. aq. $Na_2S_2O_3$ (5 mL) and sat. aq. $NaHCO_3$ (1 mL). The aqueous layer was extracted with CH_2Cl_2 (3 × 20 mL), and the combined organic layers were dried ($MgSO_4$), filtered and the solvent removed *in vacuo*. The

crude residue was purified by flash column chromatography, eluting with 30% EtOAc in petroleum ether to give **21** as a yellow solid (107 mg, 68%). **δ_H** (400 MHz, $CDCl_3$) 7.17 (1H, dd, *J* 15.5, 11.0, 7-H), 6.57 (1H, dd, *J* 15.0, 10.5, 9-H), 6.27 – 6.04 (3H, m, 6-H, 8-H and 10-H), 5.97 (1H, dq, *J* 15.0, 7.0, 11-H), 5.27 (1H, d, *J* 3.0, 6′-*H*H), 5.21 (1H, d, *J* 3.0, 6′-H*H*), 4.16 (3H, s, OCH_3), 3.02 (2H, m, 2-H_2), 2.63 (2H, t, *J* 7.0, 4-H_2), 1.96 (2H, m, 3-H_2), 1.83 (3H, d, *J* 7.0, 12-H_3). **δ_C** (101 MHz, $CDCl_3$) 199.9 (C-5), 196.4 (C-1), 168.2 (C-5′), 166.7 (C-2′), 149.0 (C-4′), 143.2 (C-7), 142.3 (C-9), 135.8 (C-11), 131.5 (C-10), 128.5 (C-8), 128.2 (C-6), 105.3 (C-1′), 96.2 (C-6′), 63.4 (OCH_3), 42.1 (C-2), 39.5 (C-4), 18.7 (C-12), 18.5 (C-3). **IR** (ν_{max}/cm^{-1}) 2956, 1770, 1689, 1579. **HRMS** (ESI) calc. for $[C_{18}H_{20}O_5+H]^+$ 317.1377, found 317.1384.

Diels-Alder adduct 22

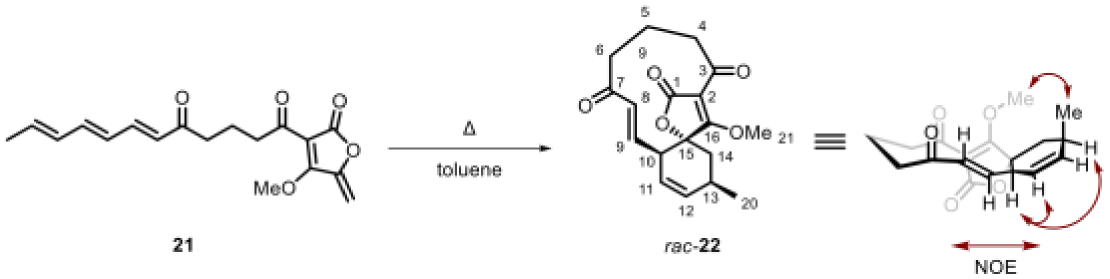

Tetronate **21** (51 mg, 0.161 mmol) was dissolved in toluene (4 mL) and the solution was heated in a sealed tube at 110 °C for 6.5 h. The reaction mixture was allowed to cool to room temperature and the solvent was removed *in vacuo*. The crude residue was purified by flash column chromatography, eluting with 40% EtOAc in petroleum ether (40-60 °C) to give **22** as a colourless oil (22 mg, 43%). **δ_H** (500 MHz, $CDCl_3$) 6.29 (1H, d, *J* 16.5, 8-H), 6.23 (1H, dd, *J* 16.5, 9.0, 9-H), 5.90 (1H, dt, *J* 10.0, 3.0, 12-H), 5.63 (1H, dt, *J* 9.5, 2.5, 11-H), 4.09 (3H, s, OCH_3), 3.33 (1H, dt, *J* 8.5, 2.5, 10-H), 3.12 (1H, ddd, *J* 14.0, 9.5, 4.0, 4-*H*H), 2.69 (1H, m, 6-*H*H), 2.55 (1H, m, 13-H), 2.49 (1H, m, 6-H*H*), 2.39 (1H, ddd, *J* 14.0, 8.0, 4.0, 4-H*H*), 2.30 (1H, dd, *J* = 14.5, 7.0, 14-*H*H), 2.20 (1H, dtdd, *J* 13.5, 8.2, 5.2, 4.0 Hz, 5-*H*H), 2.04 – 1.92 (1H, m, 5-H*H*), 1.78 (1H, dd, *J* 14.5, 6.0, 14-H*H*), 1.17 (3H, d, *J* 7.0, 13-CH_3). **δ_C** (126 MHz, $CDCl_3$) 202.3 (C-7), 198.4 (C-3), 179.2 (C-16), 169.8 (C-1), 143.2 (C-9), 137.2 (C-12), 134.0 (C-8), 123.1 (C-11), 108.5 (C-2), 86.0 (C-15), 63.2 (OCH_3), 46.8 (C-10), 41.6 (C-4), 39.2 (C-6), 36.6 (C-14), 28.9 (C-13), 23.2 (C5), 20.7 (13-CH_3). **HRMS** (ESI) calc. for $[C_{18}H_{20}O_5+Na]^+$ 339.1203, found 339.1197.

1.7 *In vitro* Enzyme Assays

1.7.1 AbmU Assays

In a total reaction volume of 200 µL (Tris buffer (20 mM Tris, 150mM NaCl), at pH 8.0, with 10% MeCN), **16** or **17** (5 mM) was incubated with AbmU (1.05 mM). Assays were initiated by the addition of cold enzyme solution. After 10 minutes the reaction was terminated by the addition of ice-cold MeCN (400 µL) and the reaction mixture was then clarified by centrifugation (14000 rpm, 4 min) to remove precipitated protein. The reaction mixture was then extracted with EtOAc (2 × 1 mL), and the organic extract dried under a stream of nitrogen gas. The resulting residue was re-dissolved in MeCN (100 µL) for LC-MS analysis. Control reactions were performed according to the same conditions but without AbmU.

1.7.2 AbyU Assays

In a total reaction volume of 200 µL (Tris buffer (20 mM Tris, 150mM NaCl), at pH 8.0, with 10% MeCN), **16** or **17** (5 mM) was incubated with AbyU (55 µM). Assays were initiated by the addition of cold enzyme solution. After 10 minutes the reaction was terminated by the addition of ice-cold MeCN (400 µL) and the reaction mixture was then

clarified by centrifugation (14000 rpm, 4 min) to remove precipitated protein. The reaction mixture was then extracted with EtOAc (2 × 1 mL), and the organic extract dried under a stream of nitrogen gas. The resulting residue was re-dissolved in MeCN (100 µL) for LC-MS analysis. Control reactions were performed according to the same conditions but without AbyU.

1.8 LC-MS Analysis

Analysis of *in vitro* enzyme assays and thermal Diels-Alder reactions was carried out on a Waters 2445SFO HPLC system with a Waters 2298 diode array detector for UV between 200 and 400 nm. The system was equipped with a Phenomenex LUNA column (5 µm, C18, 100 Å, 4.6 × 250 mm) and elution was carried out with a linear gradient of 5-95% MeCN in H_2O with 0.05 % formic acid; flow rate: 1 mL/min; detection by a Waters 2424 evaporative light scattering detector (ELSD) system. Mass spectrometry was performed using a Waters QM ESI spectrometer in positive and negative modes, with detection between 150 and 1200 m/z units.

1.9 HPLC Purification

Purification of spirotetronate diastereomers was carried out by HPLC on an Agilent 1260 Infinity instrument with UV detection at 254 nm. Diastereomers were separated on a HICHROM Kromasil semi-prep column (5 µm particle size, C18,100 Å pore size, 10 × 250 mm), hexane:EtOAc = 75:25, flow = 5.0 mL/min; t_R = 8.390 (minor) and 9.392 min (major).

1.10 Chiral HPLC Analysis

Enantiomeric ratios of the products of thermal and enzymatic cycloadditions of achiral tetronate **21** were determined by HPLC on an Agilent 1260 Infinity instrument with UV detection at 230, 280 and 254 nm. Enantiomers were separated on a chiral (*R*,*R*)-Regis Whelk-O$^{(R)}$1 column, hexane:EtOAc = 70:30, flow = 1.0 mL/min, t_R = 9.29 and 11.00.

1.11 Remodelling Capping Loop

To obtain a closed loop structure for AbmU based on the X-ray crystal structure, automated loop modelling and refinement was performed using Modeller.[24] To obtain a closed loop structure, the crystal structure was input to Modeller and loop modelling/optimisation was requested for the capping loop region, defined to be from Phe55 to Gly64. (N- and C-terminal regions - before Glu36 and after Gly174, respectively - were removed as these are not relevant for the modelling of the capping loop or the docking of the substrates in the beta-barrel.) To ensure that closed structures were obtained, a harmonic upper bound restraint was placed on the distance between the C□ atoms of Pro58 and His160, such that this distance was restrained to be less than 10 Å (with standard deviation parameter set to 0.1 Å). A dihedral restraint was also placed on the peptide bond between Pro58 and Pro59 to preserve the conformation found in the crystal structure. Modeller was then run generating 1500 loop models which were optimised using the second highest MD refinement level. The loop model with the most favourable (lowest) DOPE score was selected.

1.12 ECD

1.12.1 Experimental ECD

ECD spectra of (-)-**22** were recorded using a JASCO J-815 CD spectropolarimeter between 200-400 nm; data pitch:1 nm; 5 accumulations; 10 mm cuvette; sample concentration: 0.1 mg/mL (0.3 M) in MeCN.

1.12.1 Conformer Generation and Optimisation

An initial structure of the (10*S*,13*R*,14*R*) enantiomer of **22** was drawn in Chemdraw 22.2 and a 3D structure generated in Chem3D 22.2, following minimisation using the built in MM2 minimiser. The conformation of **22** was sampled using Spartan 24 v1.1.0 (MMFF, 10000 steps, retaining unique structures <40 kj mol^{-1}), generating 18 conformers. The conformational ensemble was optimised using Gaussian 16 at the M06-2X/6-31+g(d,p) level, incorporating frequency calculations and solvent modelling by density (SMD) using acetonitrile as the solvent. A Boltzmann distribution (298.15K) was used to calculate the relative proportion of each conformer in the ensemble.

1.12.2 ECD Calculations

ECD calculations of the ensemble were carried out using TD-DFT (Number of excited states = 10) at the same level of theory using Gaussian 16, which was also used to generate ECD spectra under default settings (Peak halfwidth at half height = 0.333 eV), and the ECD spectra were weighted according to previously calculated Boltzmann proportions. ECD spectra were calculated between 200 – 400 nm and y-axis units given as $\Delta\varepsilon$ (L mol^{-1} cm^{-1}). The contribution to the calculated ECD spectra of the top 5 conformers are shown in Figure S12 and the comparison of the experimental and calculated ECD spectra are shown in Figure S13.

5. Supplementary Figures

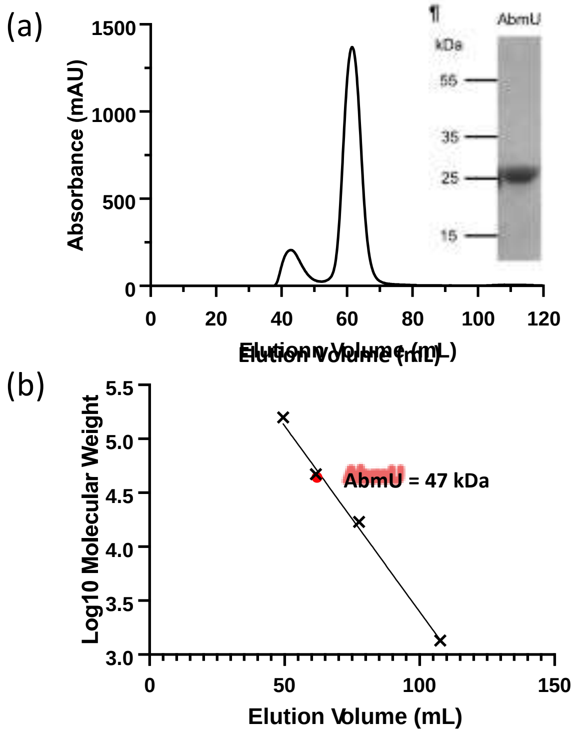

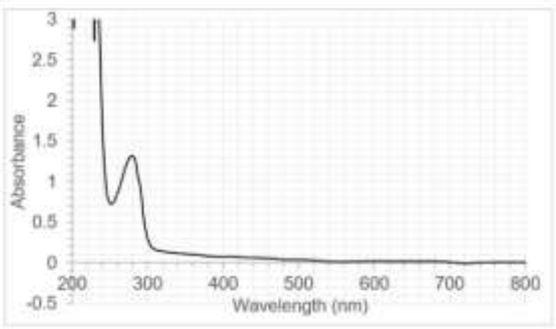

(c)

Figure S1. (a) Chromatogram showing the elution profile of AbmU from a Superdex 75 16/60 column (GE Healthcare) pre-equilibrated in 20 mM Tris-HCl, 150 mM NaCl, pH 8.0. SDS-PAGE analysis of purified recombinant AbmU is shown as an inset. **(b)** Comparative analysis of the elution volume of AbmU (red circle) with those of standards (γ-globulin, 158 kDa; ovalbumin, 44 kDa; myoglobin, 17 kDa; vitamin B_{12} 1.35 kDa). Protein standards were analysed using the same column and buffer conditions as those used for AbmU. The theoretically calculated molecular mass of an AbmU dimer, based on amino acid composition is 47 kDa, including vector encoded his-tag and linker. **(c)** UV-vis spectra of AbmU (1 mg/ml).

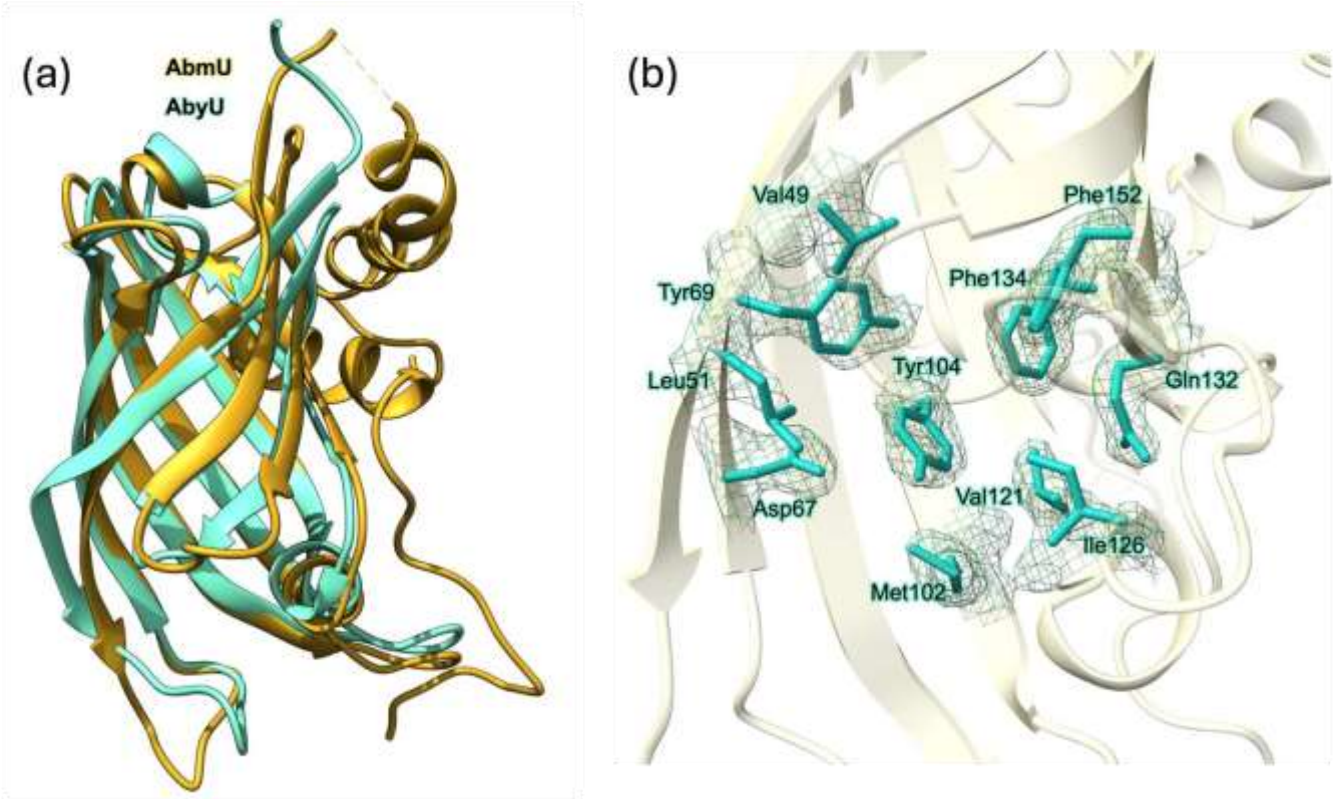

Figure S2. (a) Superimposition of the X-ray crystal structures of AbmU (6YMN; gold) and AbyU (5DYV; turquoise). **(b)** Close up of the AbmU active site with residues labelled and shown in stick format (turquoise). The refined 2FoFc electron density (contoured at 1.1□) for each residue is shown as a green mesh.

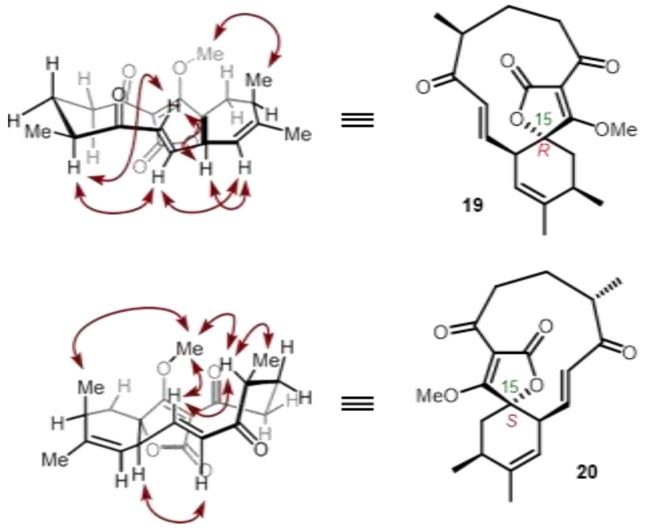

Figure S3. NOE Correlations in the major and minor products from thermal cycloaddition of **16**.

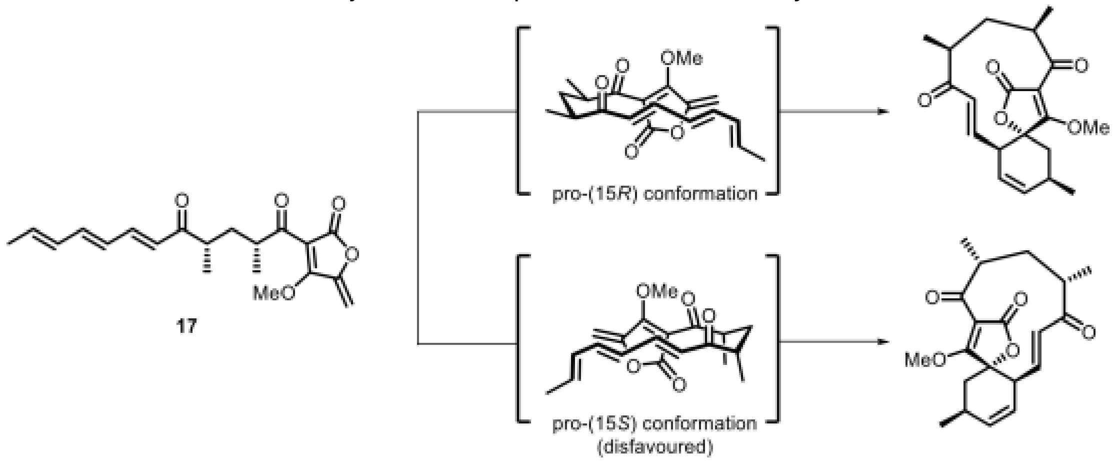

Figure S4. Reactive conformations of substrate 17 that lead to type I (15*R*) and type II (15*S*) products.

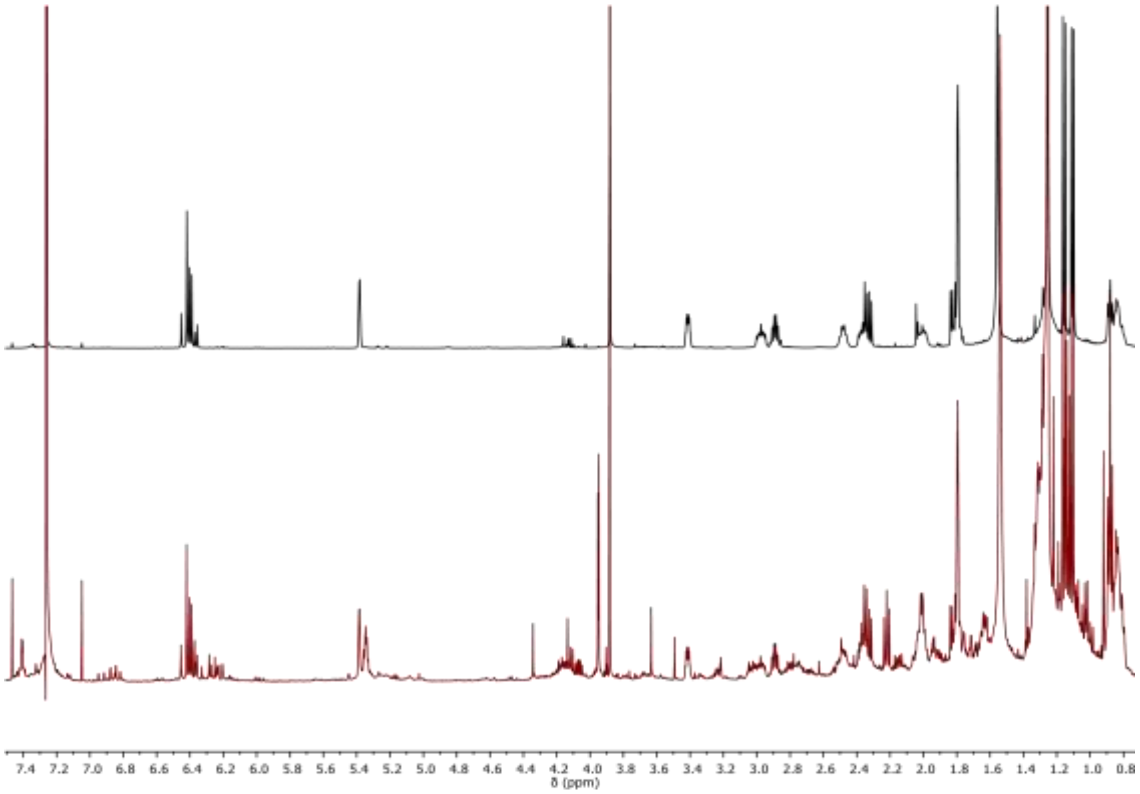

Figure S5. ^{1}H NMR comparison of major synthetic Diels-Alder adduct **19** (black) with Diels-Alder adduct from incubation of linear **16** with AbyU (maroon).

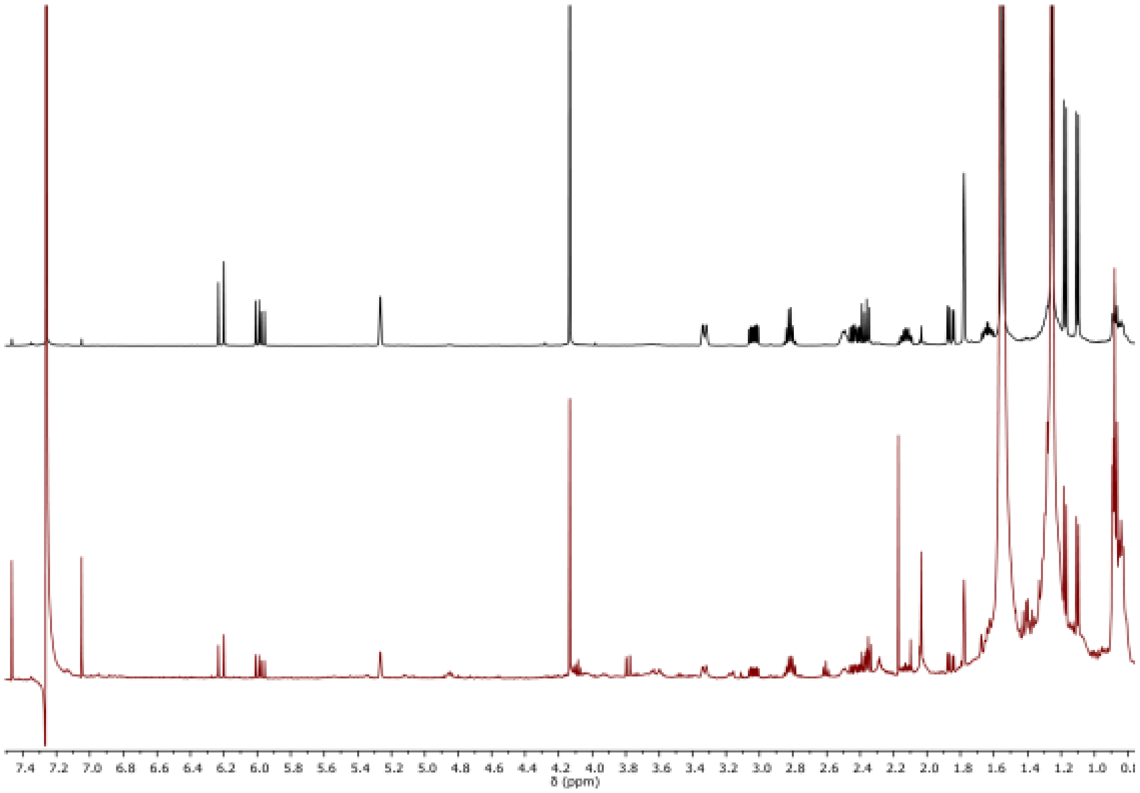

Figure S6. ^{1}H NMR comparison of minor synthetic Diels-Alder adduct **20** (black) with Diels-Alder adduct from incubation of linear **16** with AbmU (maroon).

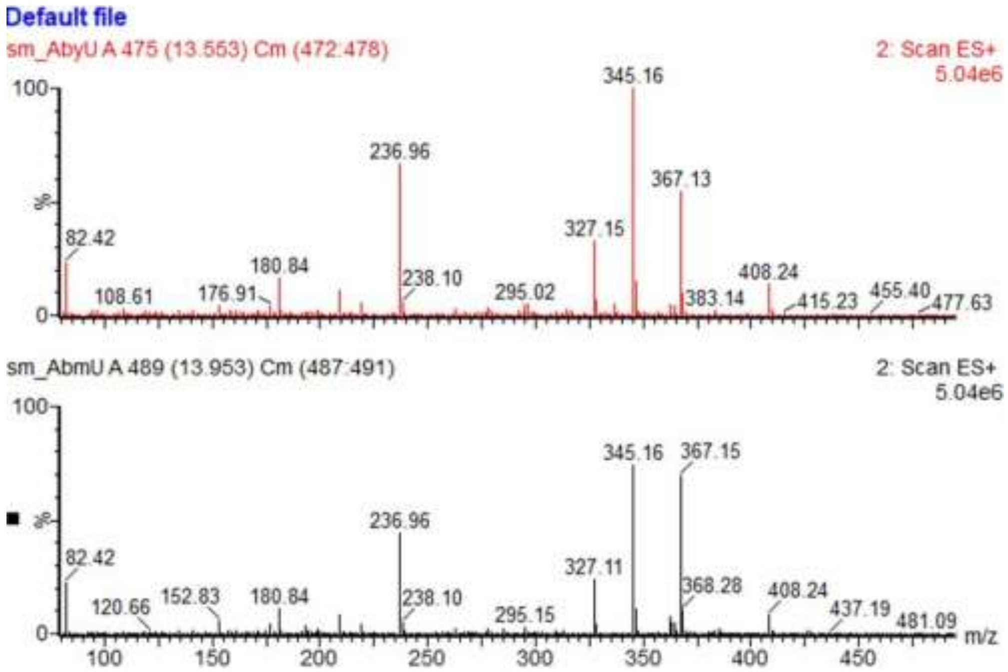

Figure S7. Mass spectra of AbmU and AbyU catalysed reaction products. Positive ion mode ESI MS of **19** (major top) recovered from AbyU catalysed [4+2] cycloaddition reaction and **20** (minor bottom) recovered from AbmU catalysed [4+2]-cycloaddition reaction. Product masses are consistent with the synthetic products.

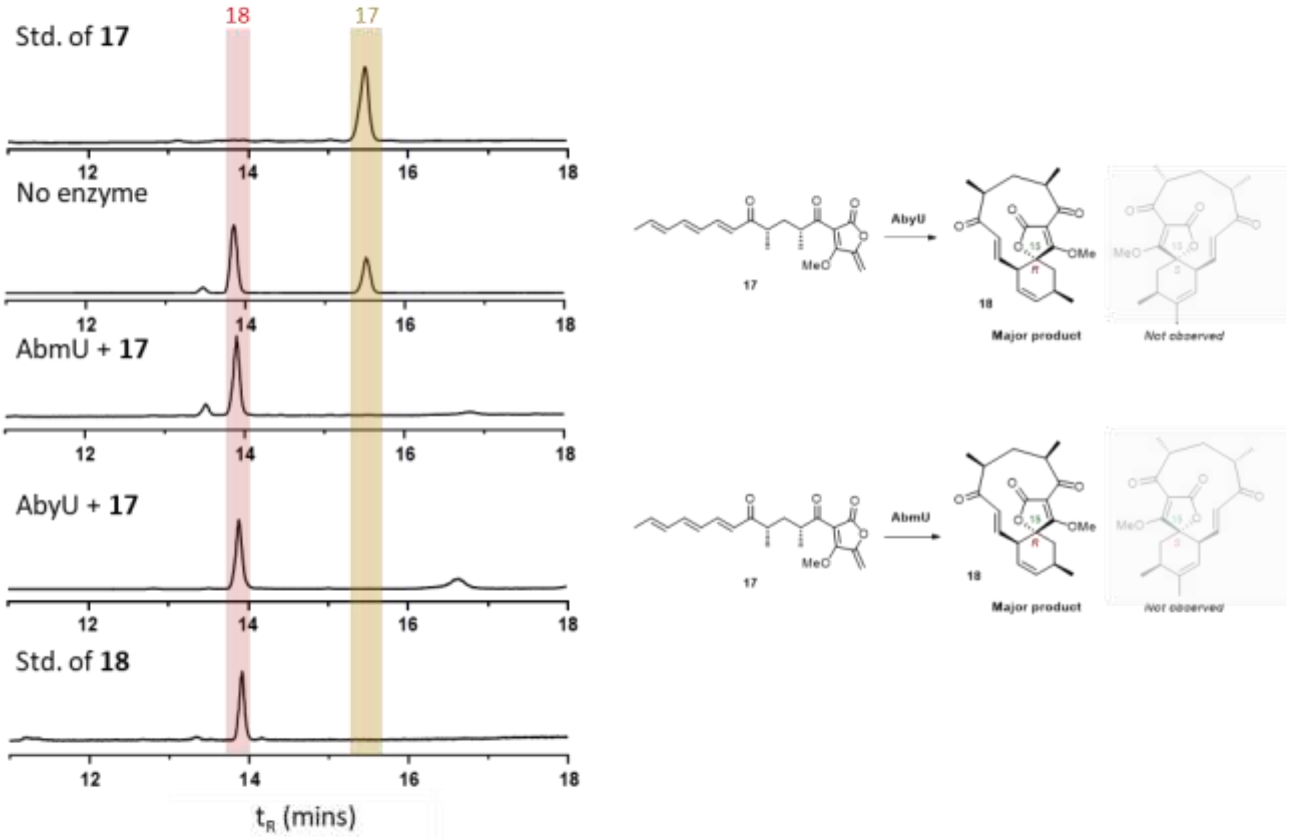

Figure S8. LC-MS ELSD chromatograms showing Diels-Alder products arising from incubation of **17** with AbmU and AbyU.

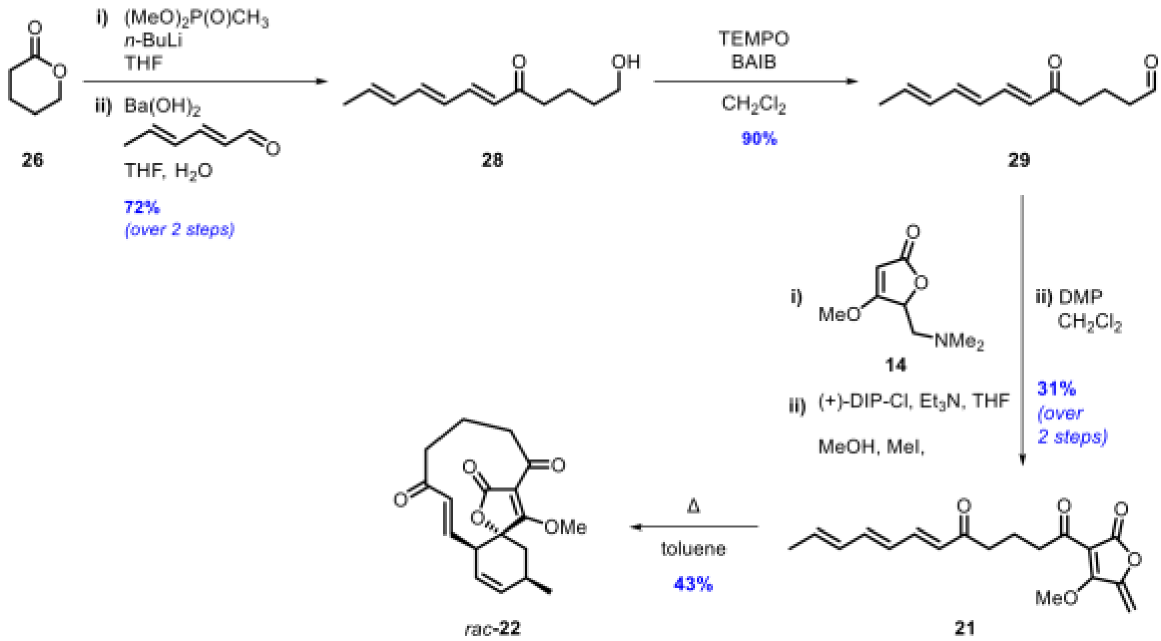

Figure S9. Synthetic route to achiral substrate analogue **21**.

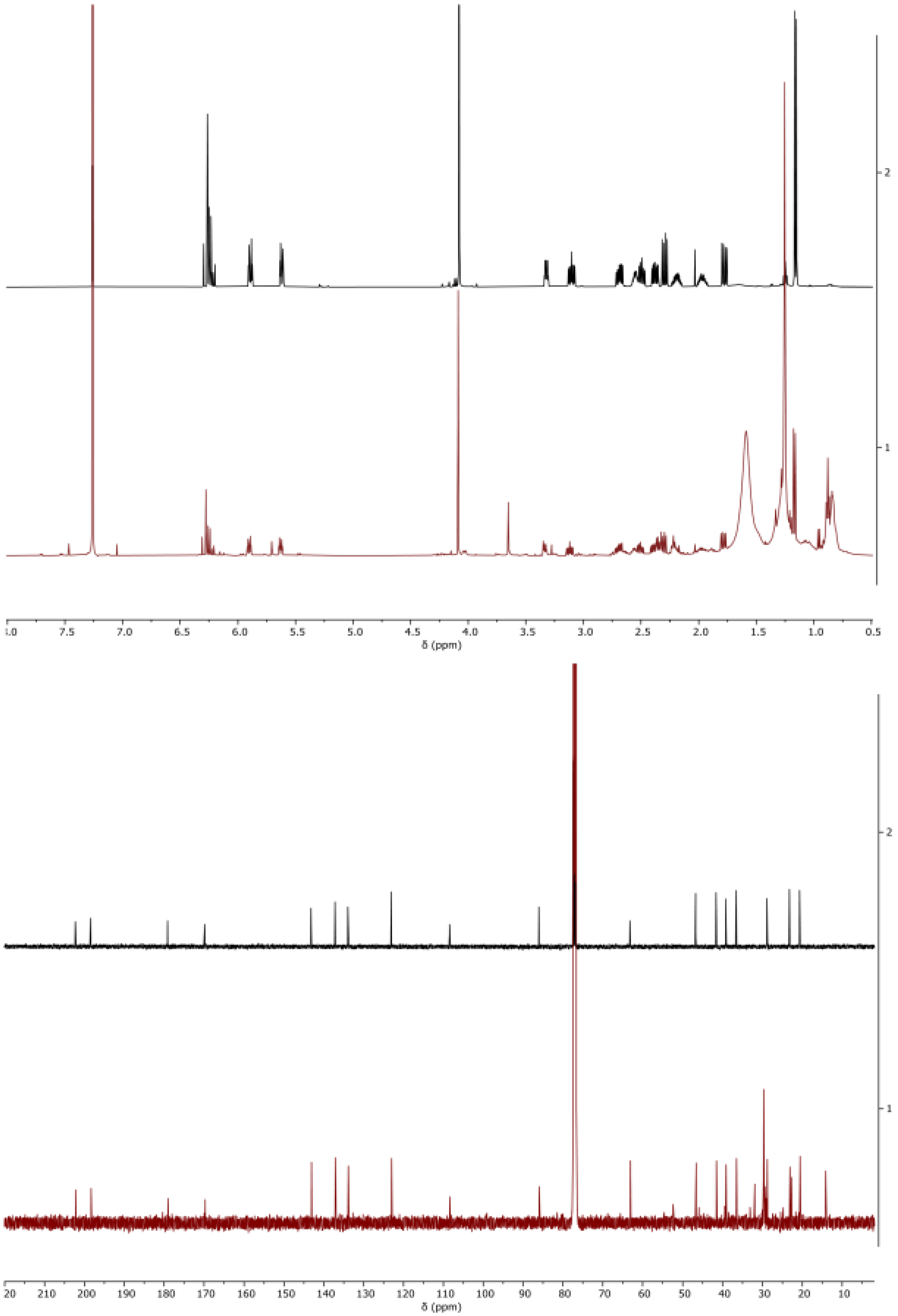

Figure S10. ^{1}H and ^{13}C NMR comparison of synthetic Diels-Alder adduct *rac*-**22** (black) with Diels-Alder adduct (-)-**22** from incubation of linear **21** with AbyU (maroon).

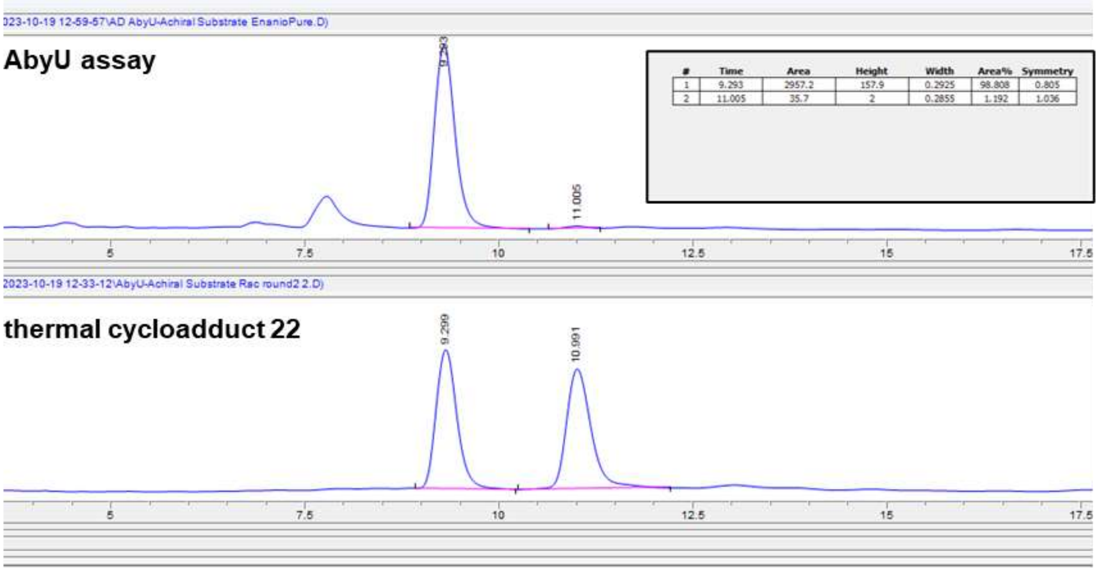

#	Time	Area	Height	Width	Area%	Symmetry
1	9.293	2957.2	157.9	0.2925	98.808	0.805
2	11.005	35.7	2	0.2855	1.192	1.036

Figure S11. Chiral HPLC chromatograms of thermal cycloadduct **22** and enantiopure **22** from AbyU enzymatic reaction.

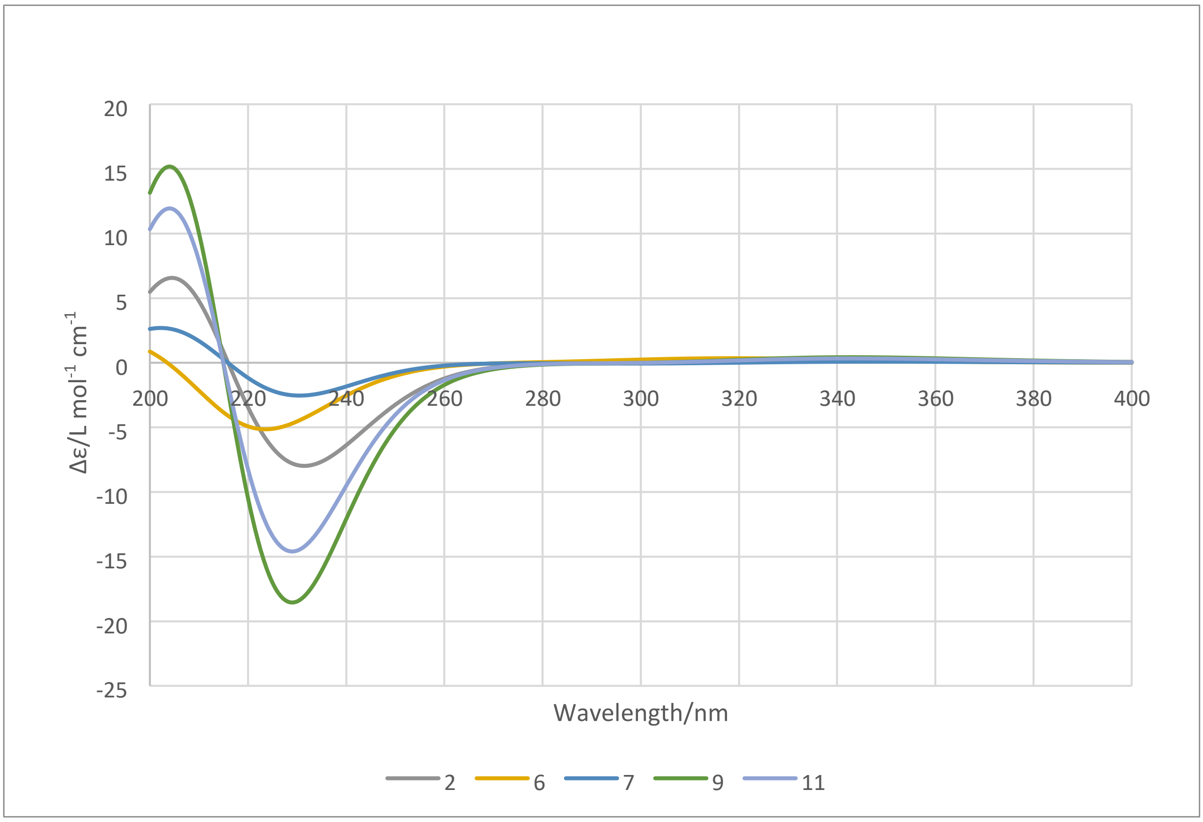

Figure S12: Contribution to ECD of top 5 conformers.

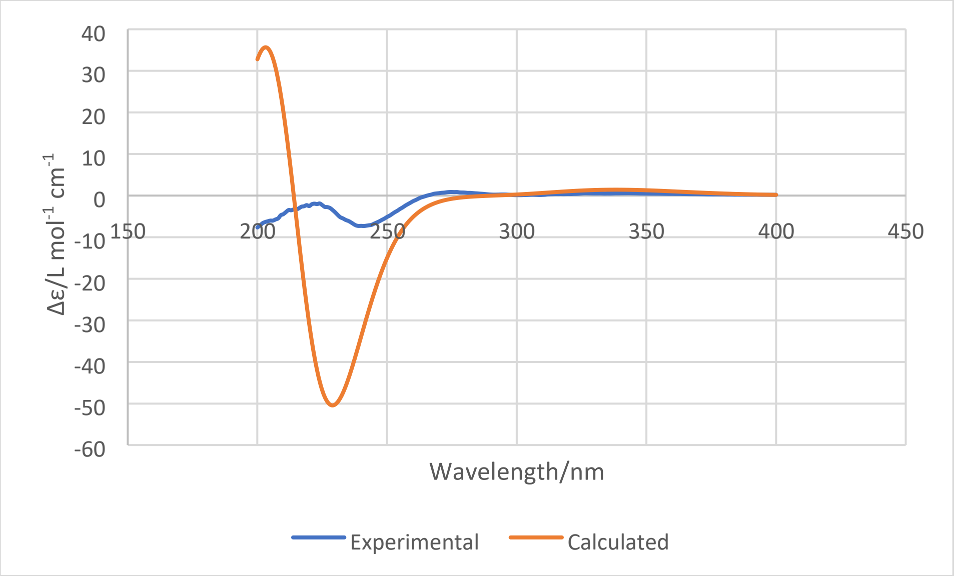

Figure S13: Calculated vs. Experimental ECD Spectra.

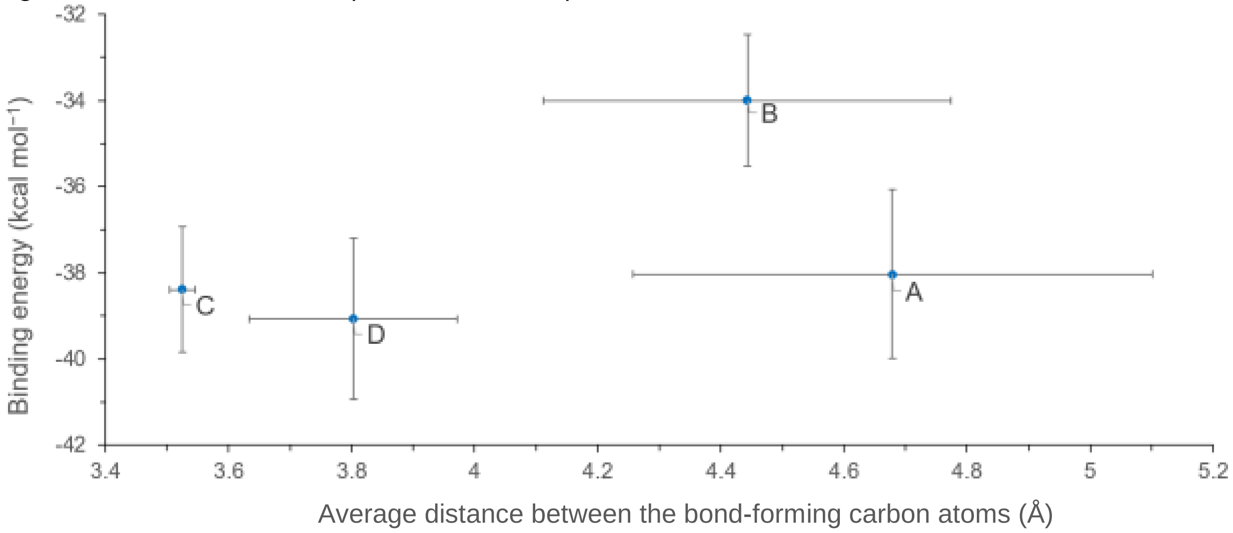

Figure S14. Binding affinity and average C-C bond distances of AbmU substrate binding poses in AbmU from MD simulation. Average binding energies (see Figure 4, bottom, in main manuscript) plotted against bond-forming carbon-carbon distances (both calculated across 10 independent MD simulations) for each representative substrate binding mode. Error bars represent standard deviations between the 10 runs.

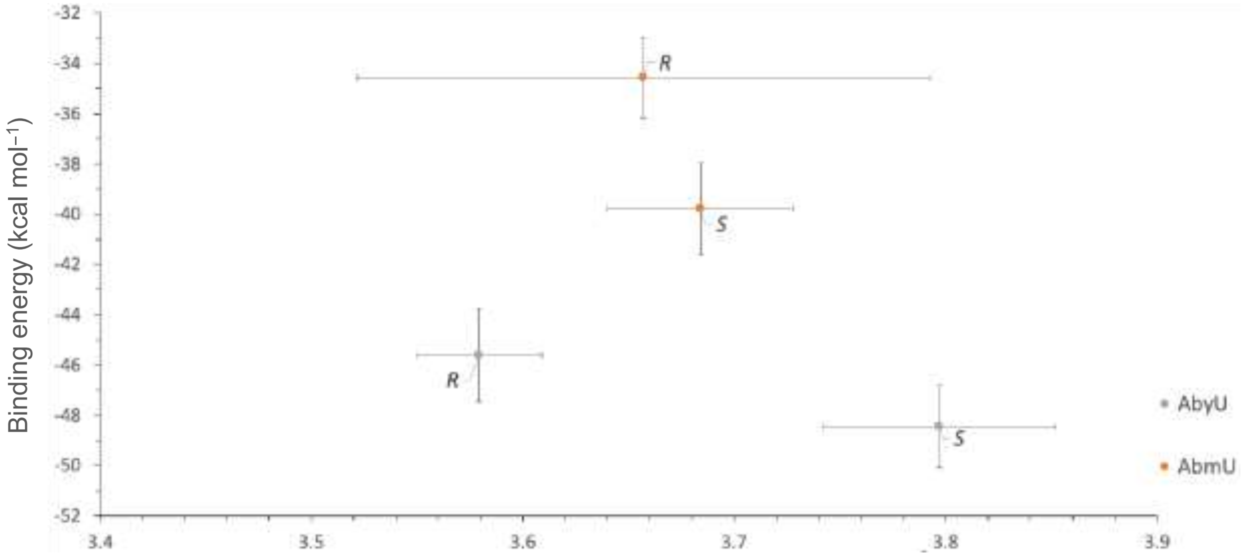

Average distance between the bond-forming carbon atoms (Å)

Figure S15. Assessment of reactivity and binding affinity of prochiral substrate **16** binding poses in Diels-Alderases AbyU and AbmU. Previously identified reactive binding modes are used as the starting point. Binding poses labelled *R* are in a C15 pro-*R* conformation, leading to product **19**. Binding poses labelled *S* are in a C15 pro-*S* conformation, leading to product **20**. Binding affinity and average C-C distances were calculated from ten independent 100 ps simulations per enzyme-substrate complex. Error bars represent standard deviations between the 10 runs.

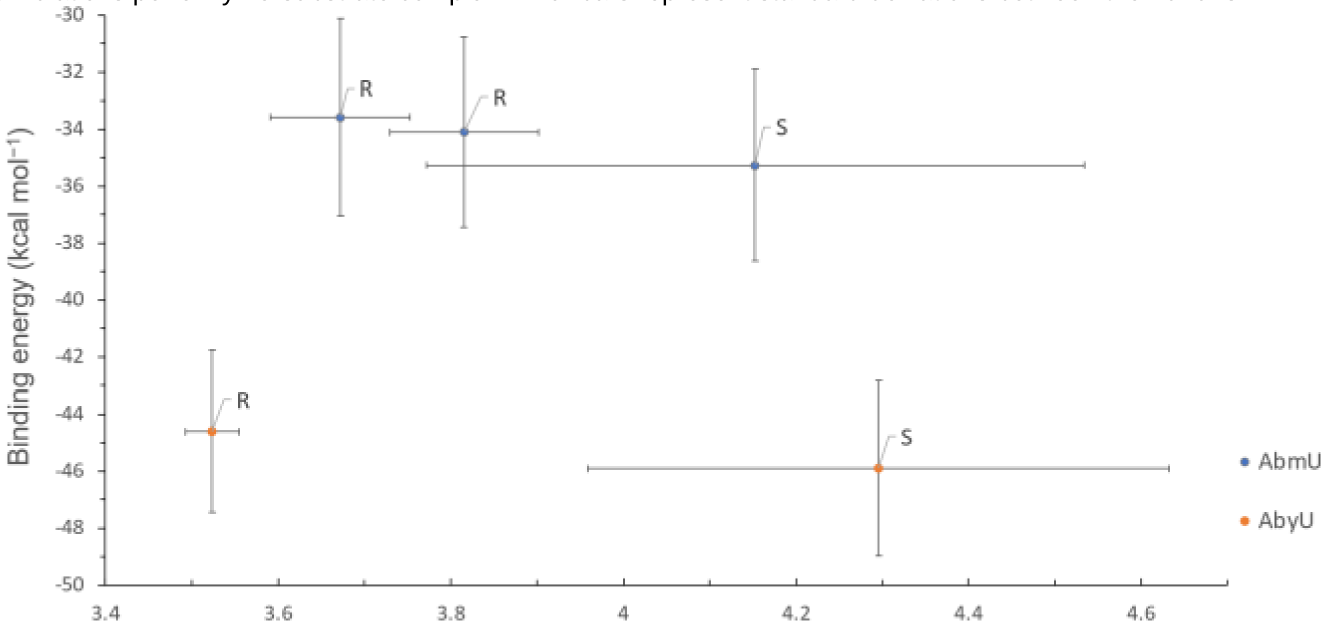

Figure S16. Assessment of reactivity and binding affinity of prochiral substrate **17** binding poses in Diels-Alderases AbyU and AbmU. Substrate binding modes are obtained by flexible docking of the substrate with AutoDock Vina[25] and selection of poses based on consistency with previously established reactive binding poses. No pro-*S* binding pose was found through automated docking for **17** in AbmU, and thus manual docking was performed here. Binding poses labelled *R* are in a C15 pro-*R* conformation, leading to product **18**. Binding poses labelled *S* are in a C15 pro-*S* conformation, leading to the enantiomer of product **18**. Binding affinity and average C-C distances were calculated from ten independent 100 ps simulations per enzyme-substrate complex. Error bars represent standard deviations between the 10 runs.

3. Supplementary Tables

Table S1. Summary of X-ray data collection and refinement statistics.

	**AbmU**
**Data collection**	
Beamline wavelength (Å)	0.91971
Space group	$P12_11$
Cell dimensions *a, b, c* (Å)	66.13, 65, 97.86
a, b, g, (°)	90.0, 93.81, 90.0
Resolution (Å)	56.44-2.05 (2.10-2.05)[a]
Rpim	4.3 (68.1)[a]
No. of reflections	978198 (25012)[a]
No. of unique reflections	52059 (3814)[a]
I/s*I*	12.25 (1.2)[a]
$CC_{1/2}$ (%)	99.8 (49.1)[a]
Completeness (%)	99.6 (97.4)[a]
Redundancy	18.8 (6.6)[a]
No. of bromide sites	7
**Refinement**	
R_{work}/R_{free}	18.9/24.3
No. of atoms	
Protein	12280
Ligand/ion	7
Water	485
B factors (Å^2)	
Protein	42.17
Ligand/ion	66.5
Water	46.4
Root mean square deviations	
Bond lengths (Å)	0.0076
Bond angles (°)	1.56
Ramachandran favoured (%)	96
Ramachandran outliers (%)	0

[a] Values in parentheses are for highest resolution shell

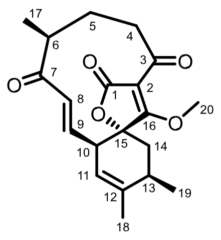

19

Table S2. Major adduct **19** NMR data (500 MHz, $CDCl_3$)

Position	δ_H (*J* in Hz)	δ_C	NOESY
1	–	169.5	–
2	–	106..8	–
3	–	198.2	–
4	2.97 (1H, ddd, *J* 13.0, 8.0, 4.0)	43.0	ND
	2.38 (1H, m)		–
5	2.02 (1H, m)	29.9	–
	1.82 (1H, m)		–
6	2.89 (1H, pd, *J* 6.5, 4.0)	45.2	8, 9, 17, 4[a]
7	–	204.6	–
8	6.44 (1H, d, *J* 16.5)	132.8	–
9	6.38 (1H, dd, *J* 16.5, 7.0)	142.1	11[a]
10	3.47 (1H, dq, *J* 7.5, 2.5)	45.8	8[a], 9, 11, 13, 14
11	5.38 (1H, m)	118.4	8, 9, 10, 18
12	–	142.2	–
13	2.48 (1H, m)	32.2	19
14	2.33 (1H, dd, *J* 14.5, 7.0)	37.8	–
	1.82 (1H, m)		–
15	–	86.0	–
16	–	179.4	–
17	1.11 (3H, d, *J* 6.5)	16.1	6
18	1.79 (3H, s)	21.0	11[a], 19[a]
19	1.16 (3H, d, *J* 7.0)	18.8	13[a], 14[a], 18[a], OCH_3[a]
20	3.88 (3H, s)	61.9	19

ND = No data obtained; [a] Indicates observed NOESY signals in C_6D_6.

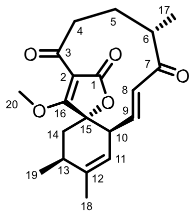

20

Table S3. Minor Adduct **20** NMR data (500 MHz, $CDCl_3$)

**Position**	**δ_H (*J* in Hz)**	**δ_C**	**NOESY**
1	–	169.8	–
2	–	108.6	–
3	–	198.8	–
4	3.03 (1H, ddd, *J* 15.5, 8.0, 4.0)	41.4	4[a]
	2.43 (1H, ddd, *J* 15.5, 10.0, 4.0)		-
5	2.13 (1H, dddd, *J* 15.5, 10.0, 6.5, 4.0)	31.0	5[a], 4[a], 6[a]
	1.64 (1H, dddd, *J* 15.5, 8.0, 6.5, 4.0)		5[a], 17[a]
6	2.82 (1H, sext., *J* 6.5)	43.5	9, 17, OCH_3
7	–	204.8	–
8	6.22 (1H, d, *J* 16.5)	133.5	10
9	5.98 (1H, dd, *J* 16.5, 10.5)	141.7	6, 11, OCH_3
10	3.33 (1H, dq, *J* 10.5, 2.0)	48.2	8, 11
11	5.27 (1H, q, *J* 2.0)	118.1	18
12	–	141.9	–
13	2.50 (1H, m)	32.5	14, 18, 19
14	2.37 (1H, dd, *J* 14.5, 7.0)	37.0	-
	1.86 (1H, dd, *J* 14.5, 5.0)		14, 19, OCH_3
15	–	85.9	–
16	–	178.7	–
17	1.10 (3H, d, *J* 6.5)	15.6	6
18	1.78 (3H, s)	21.0	11[a], 19[a]
19	1.18 (3H, d, *J* 7.5)	19.0	18[a]
20	4.13 (3H, s)	63.4	6, 9, 19

[a] Indicates observed NOESY signals in CD_3OD.

4. NMR Spectra

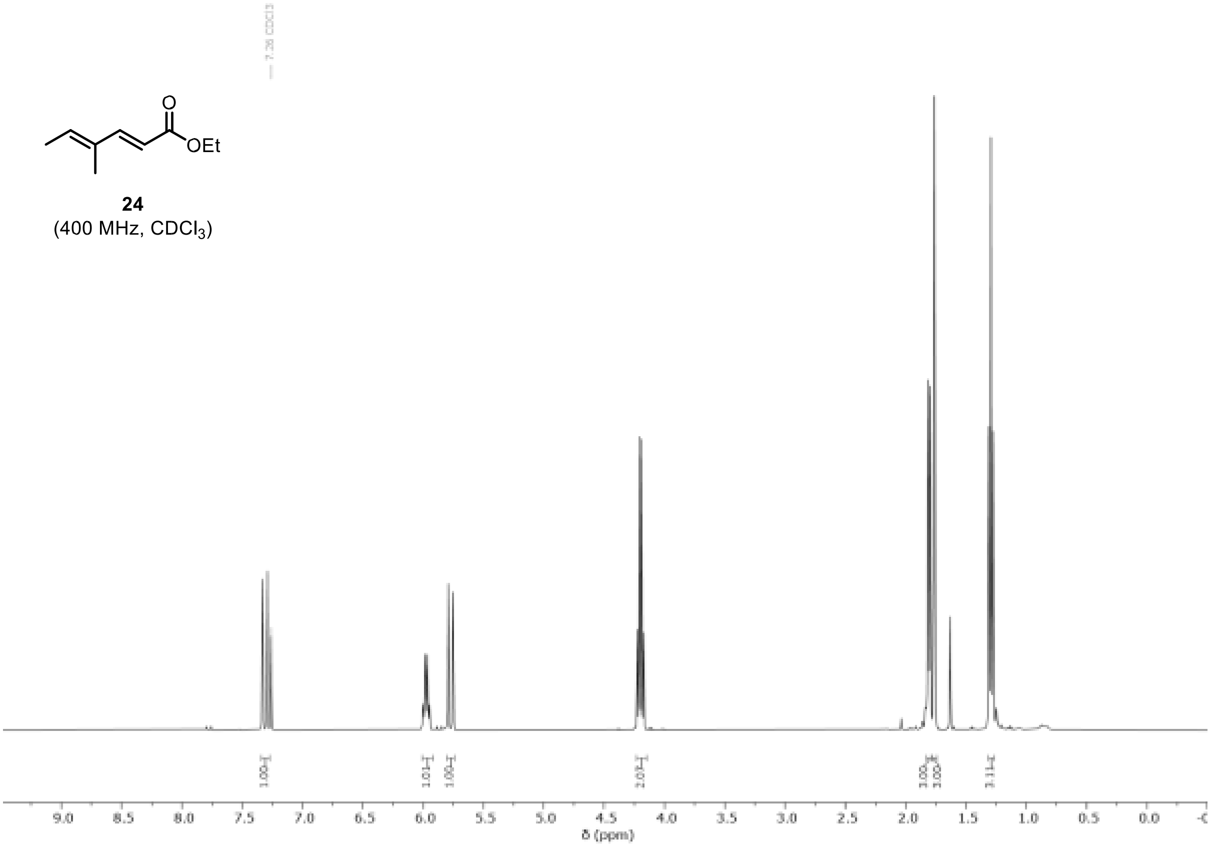

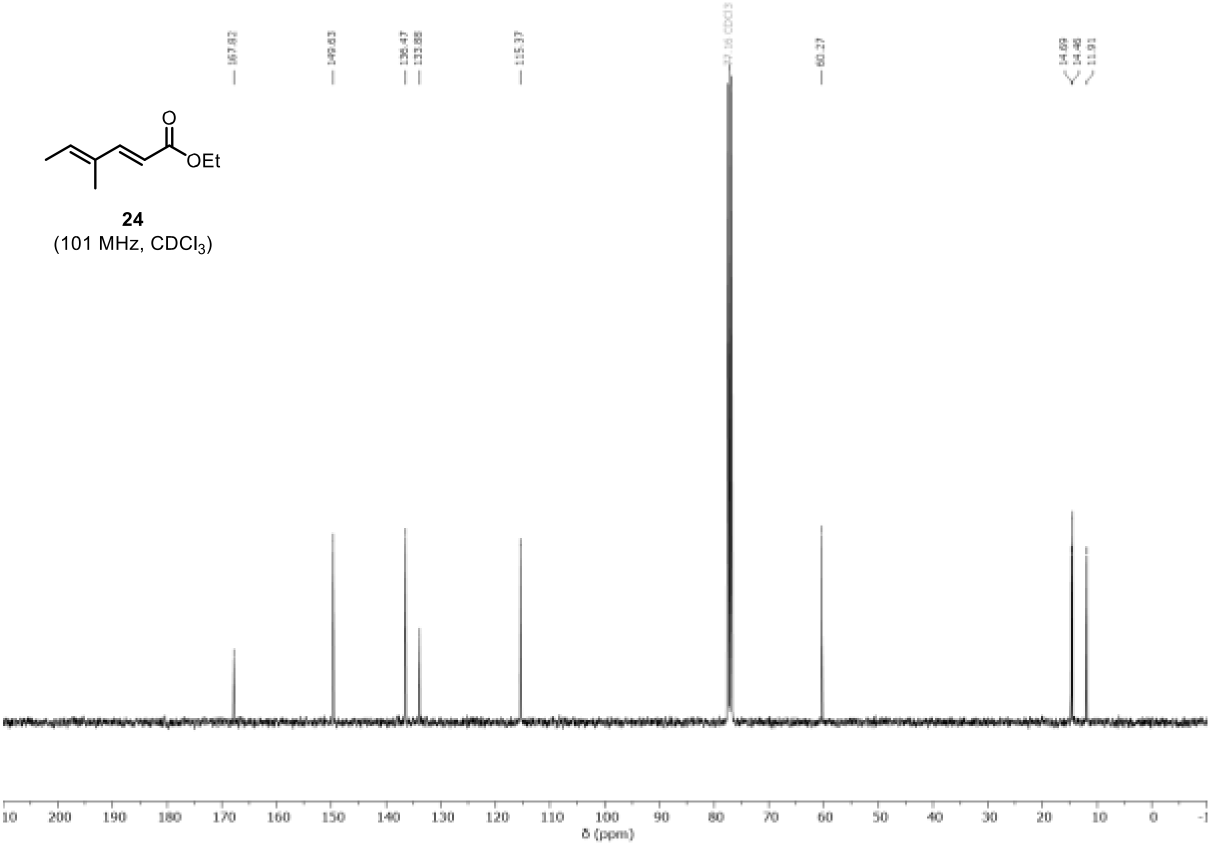

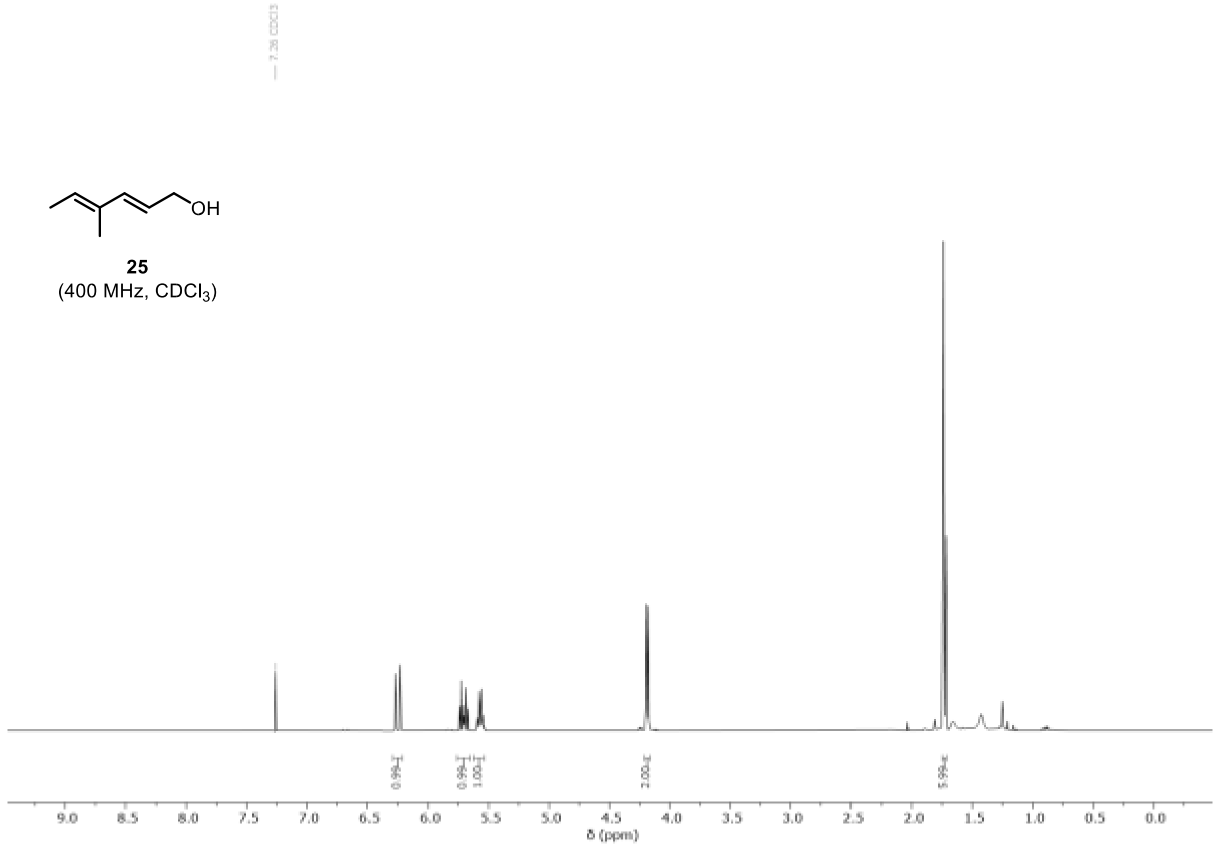

25
(400 MHz, $CDCl_3$)

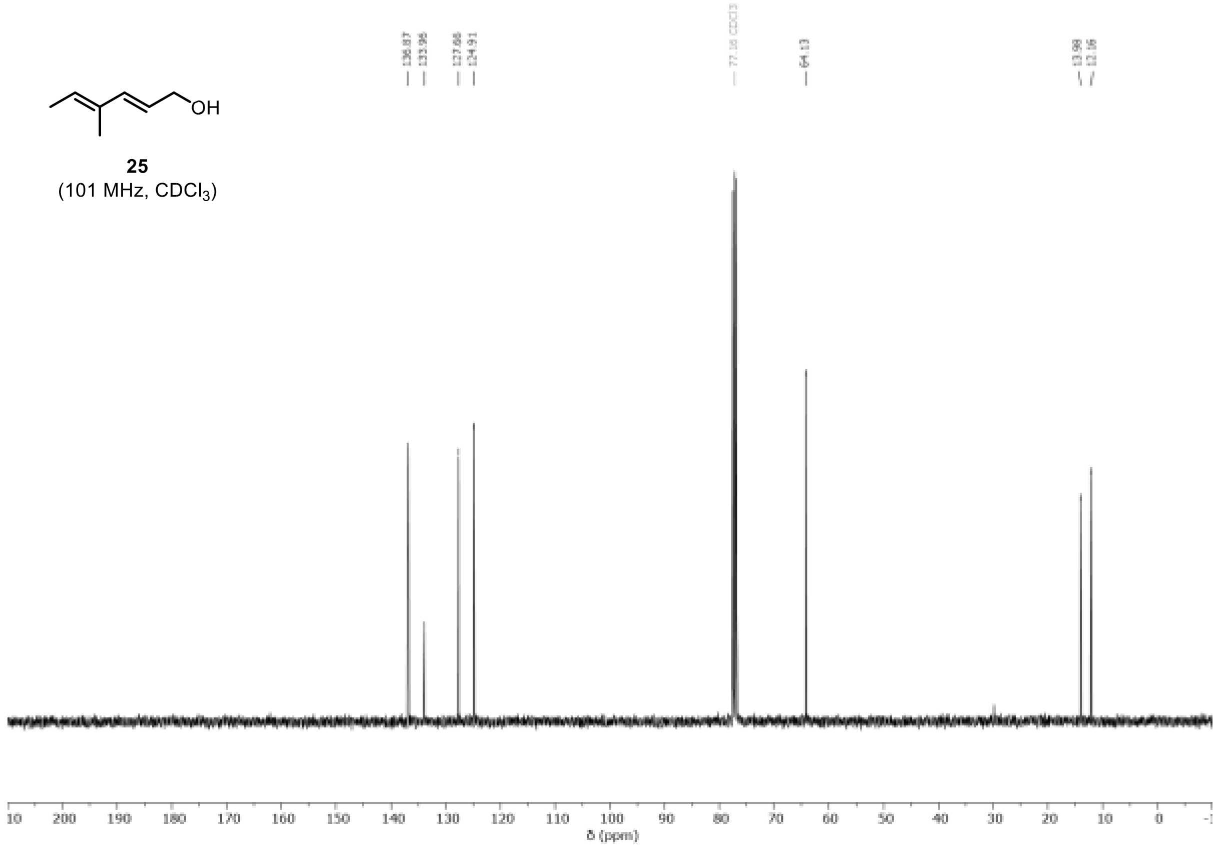

25
(101 MHz, $CDCl_3$)

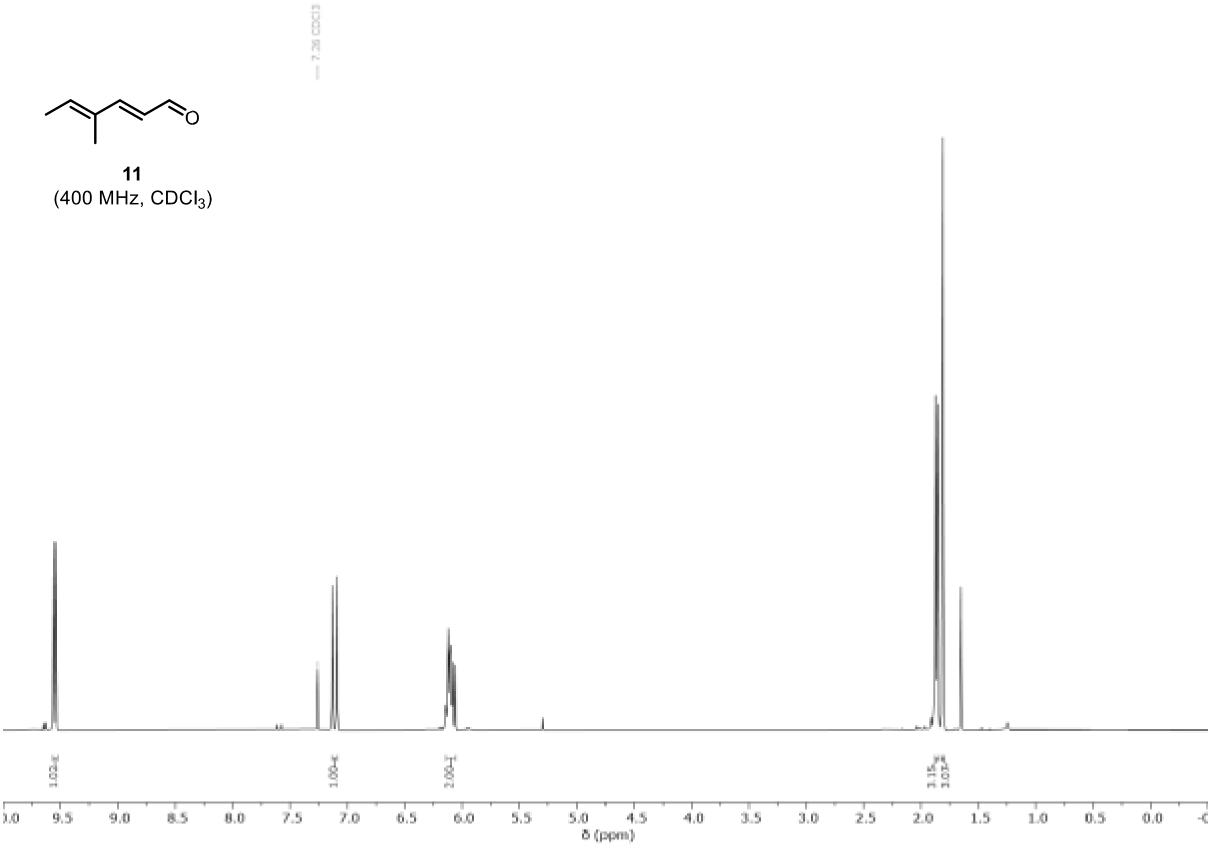
11
(400 MHz, CDCl3)
δ (ppm)

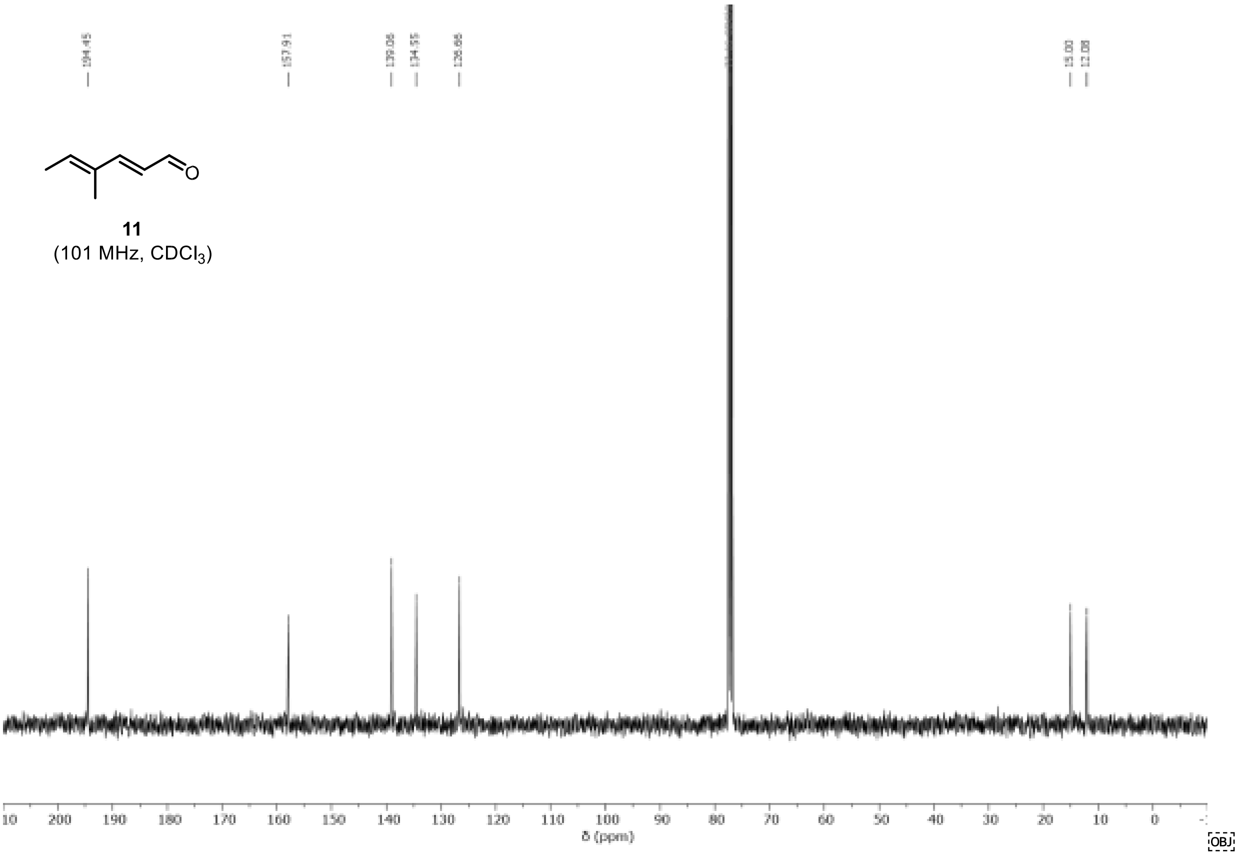
11
(101 MHz, CDCl3)
δ (ppm)

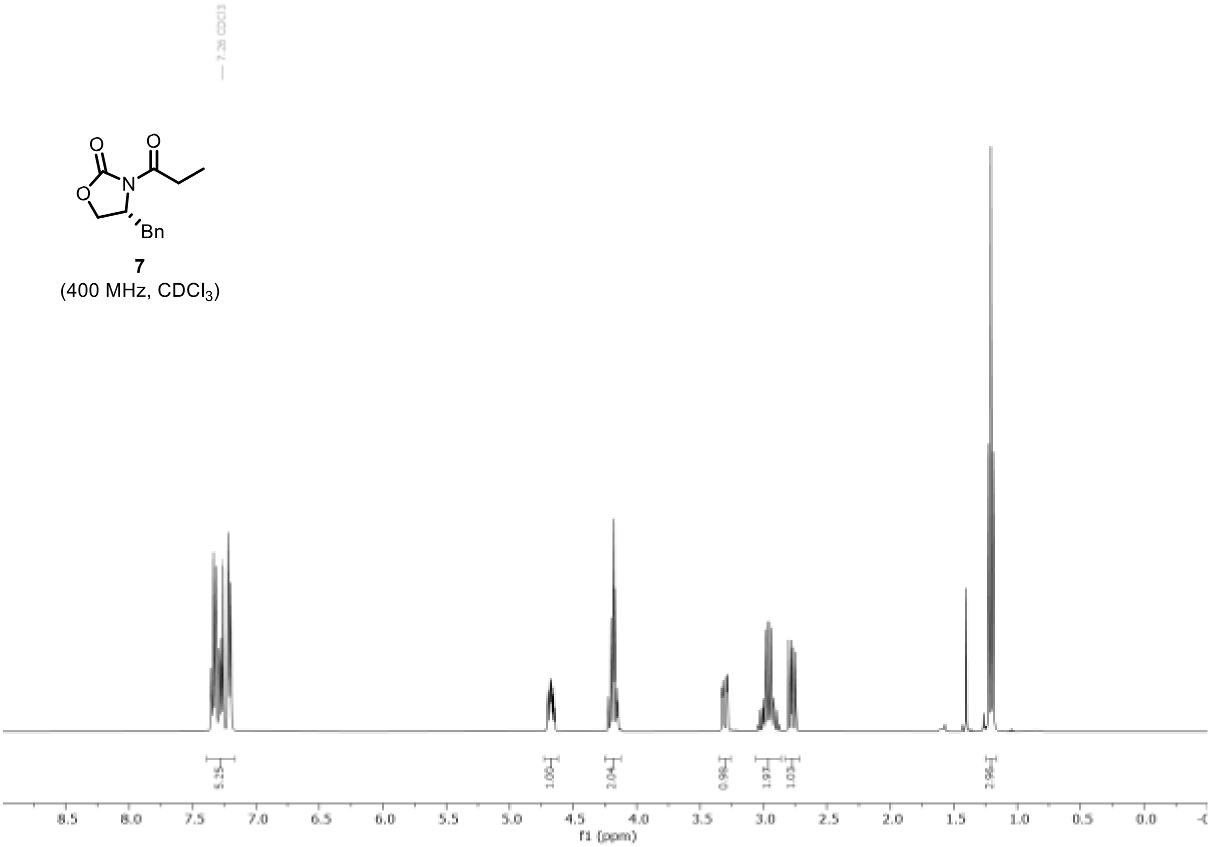
7.26 CDCl3
O
O
N
O
Bn
7
(400 MHz, CDCl3)
5.25
1.00
2.04
0.98
1.97
1.03
2.96
8.5
8.0
7.5
7.0
6.5
6.0
5.5
5.0
4.5
4.0
3.5
3.0
2.5
2.0
1.5
1.0
0.5
0.0
f1 (ppm)

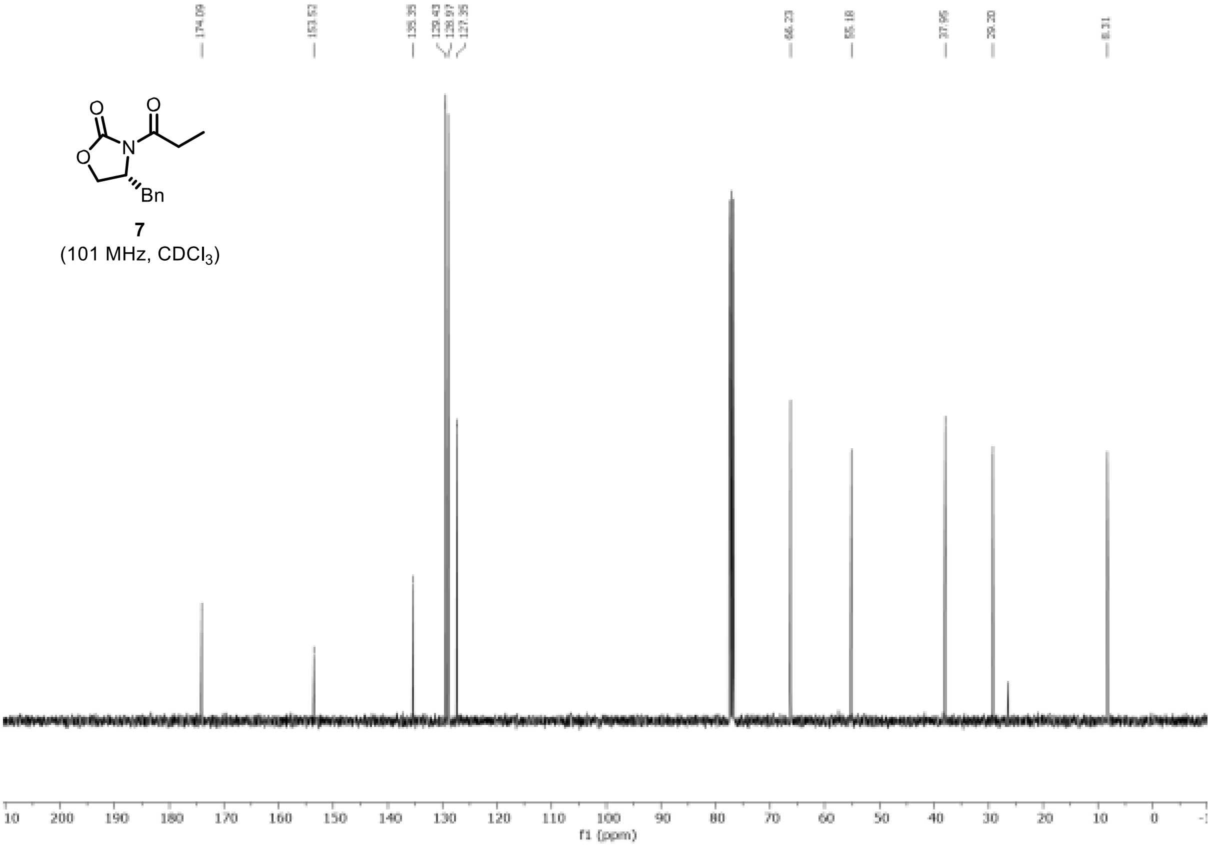
174.09
153.52
135.35
129.43
128.97
127.35
66.23
55.18
37.95
29.20
8.31
O
O
N
O
Bn
7
(101 MHz, CDCl3)
200
190
180
170
160
150
140
130
120
110
100
90
80
70
60
50
40
30
20
10
0
f1 (ppm)

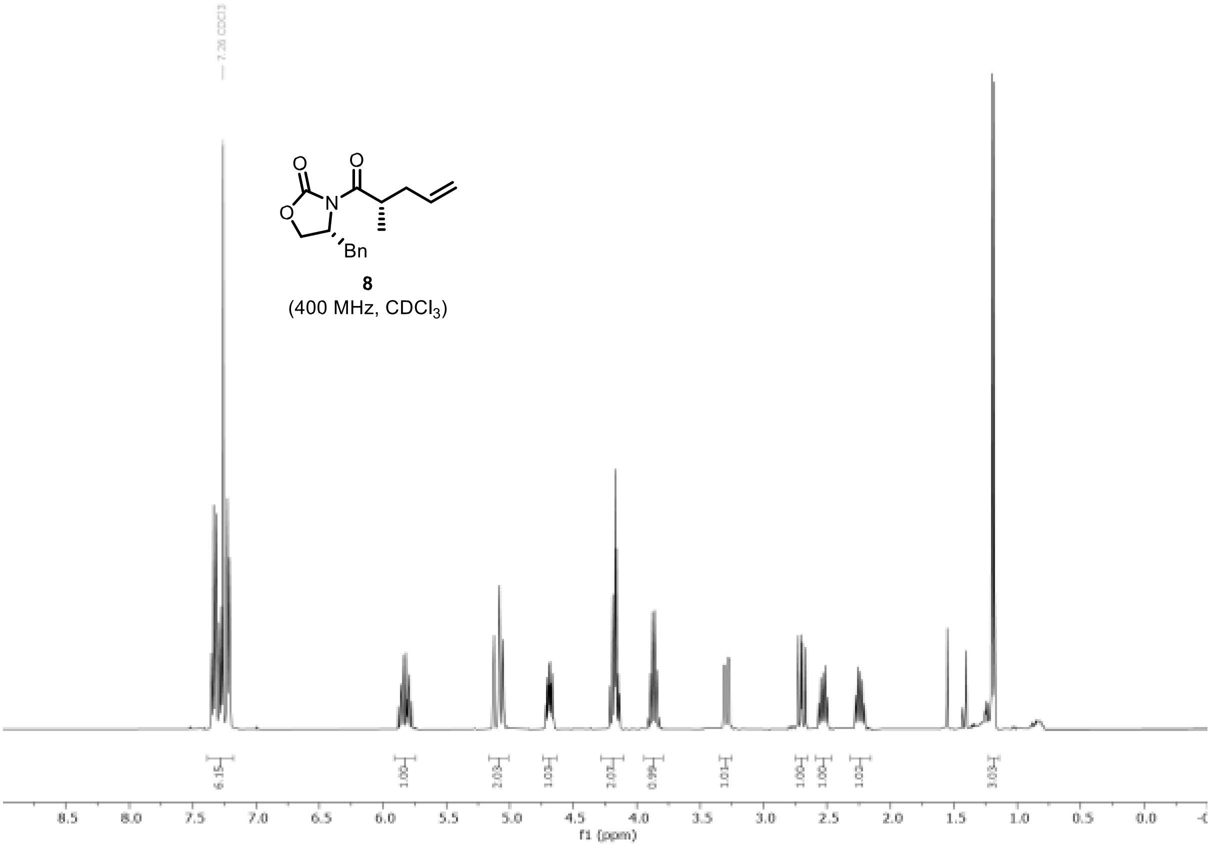
O
N
O
Bn
8
(400 MHz, CDCl$_3$)
f1 (ppm)

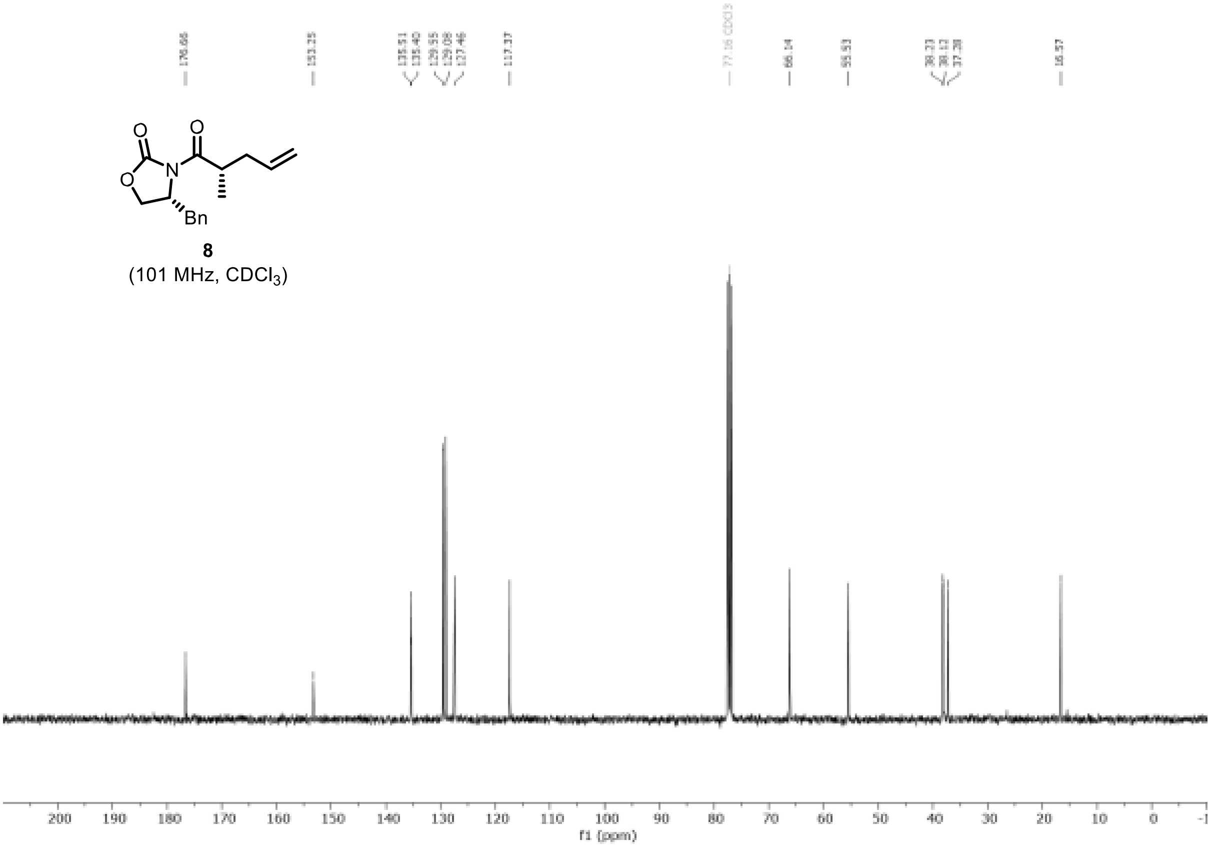
O
N
O
Bn
8
(101 MHz, CDCl$_3$)
f1 (ppm)

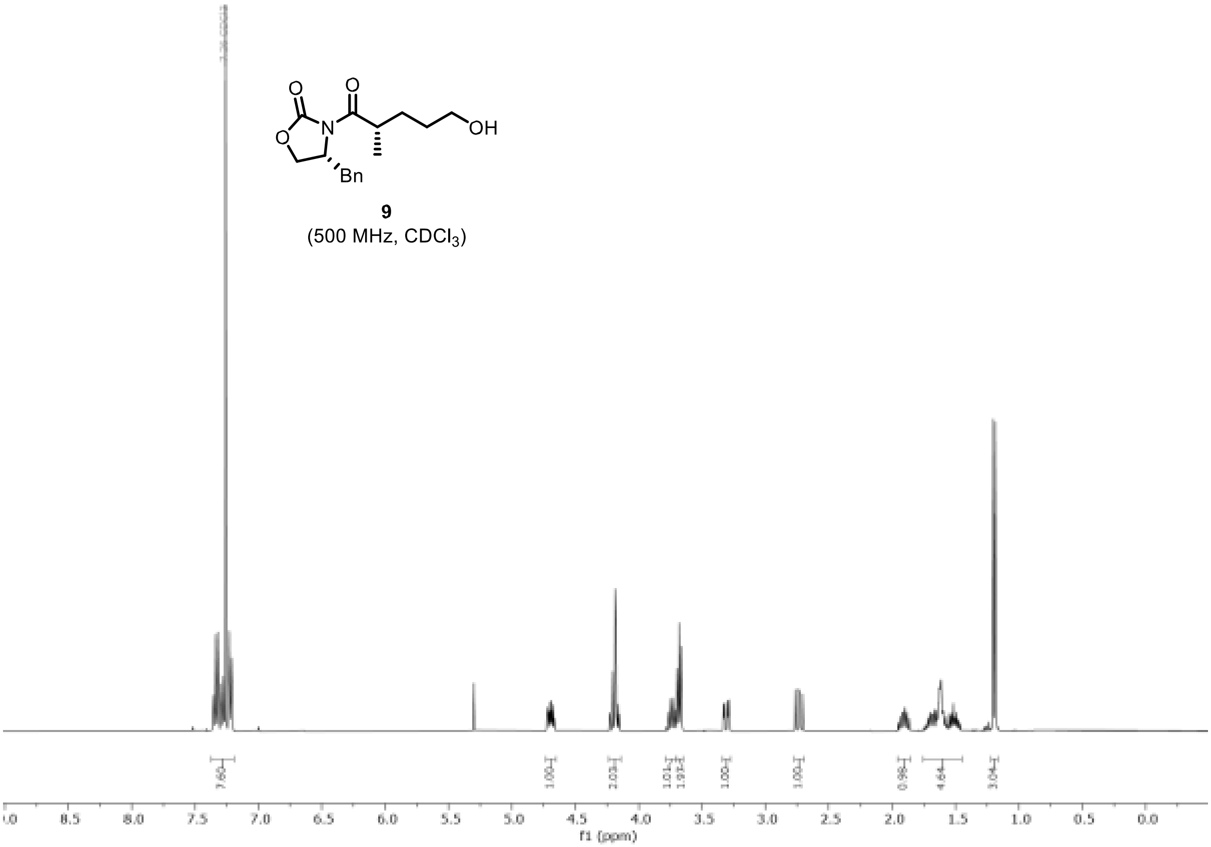
9
(500 MHz, CDCl$_3$)
OH
Bn
7.60
1.00
2.03
1.01
1.97
1.00
1.00
0.98
4.64
3.04
f1 (ppm)

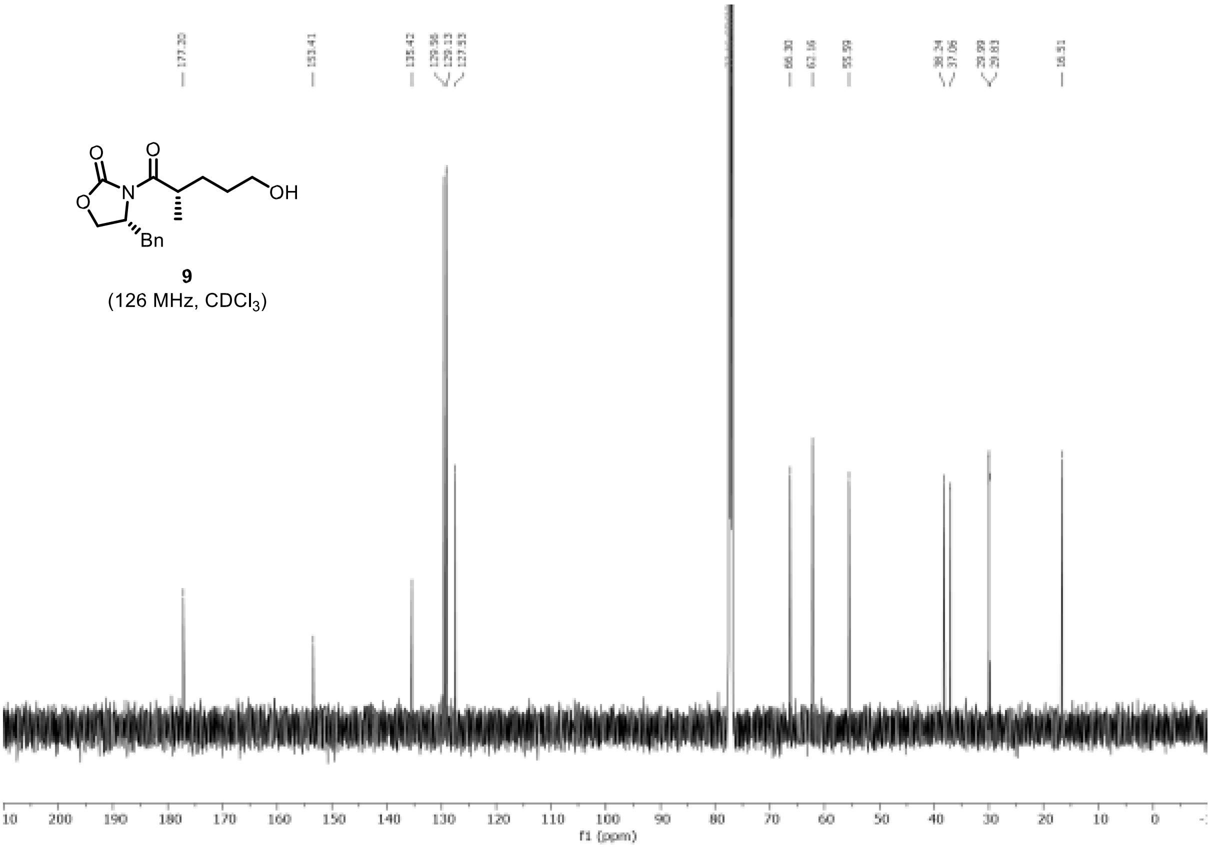
177.20
153.41
135.42
129.56
129.13
127.53
66.30
62.16
55.59
38.24
37.06
29.99
29.83
16.51
9
(126 MHz, CDCl$_3$)
OH
Bn
f1 (ppm)

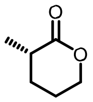

10
(400 MHz, $CDCl_3$)

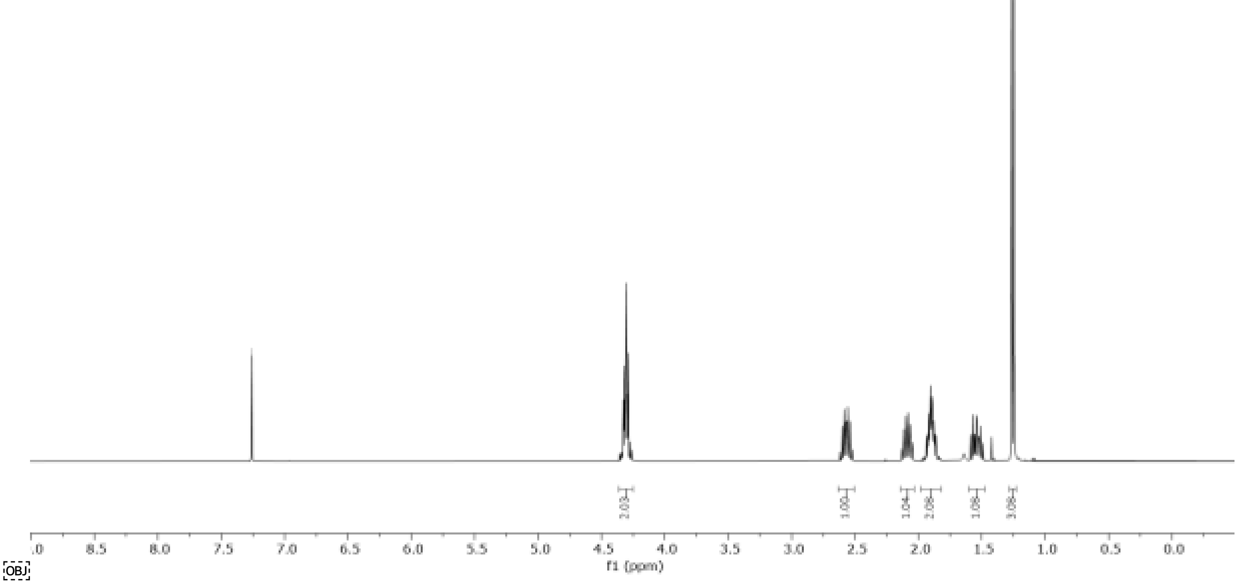

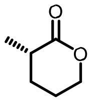

10
(101 MHz, $CDCl_3$)

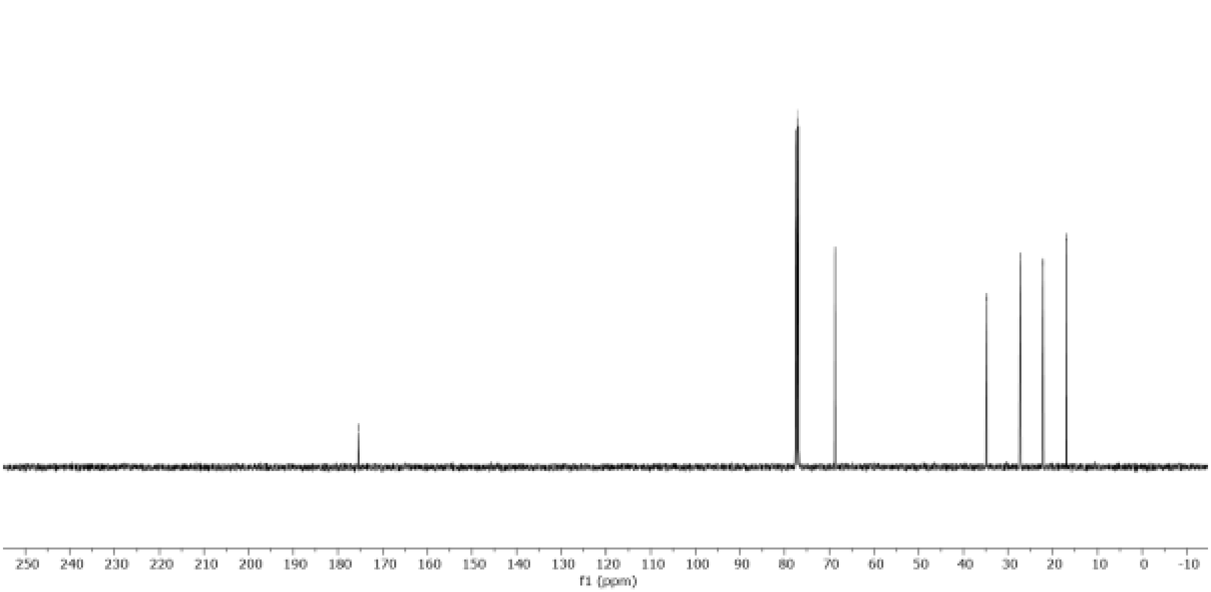

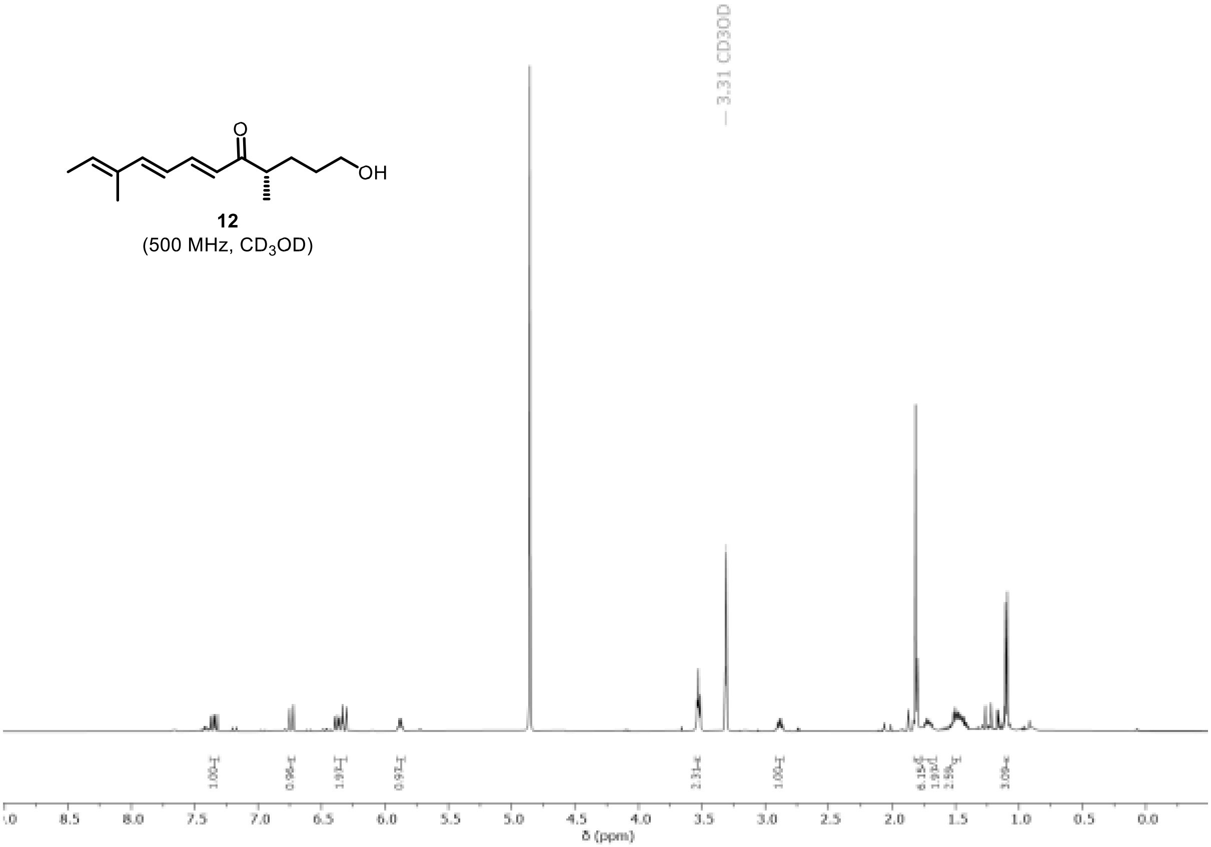

12
(500 MHz, CD_3OD)

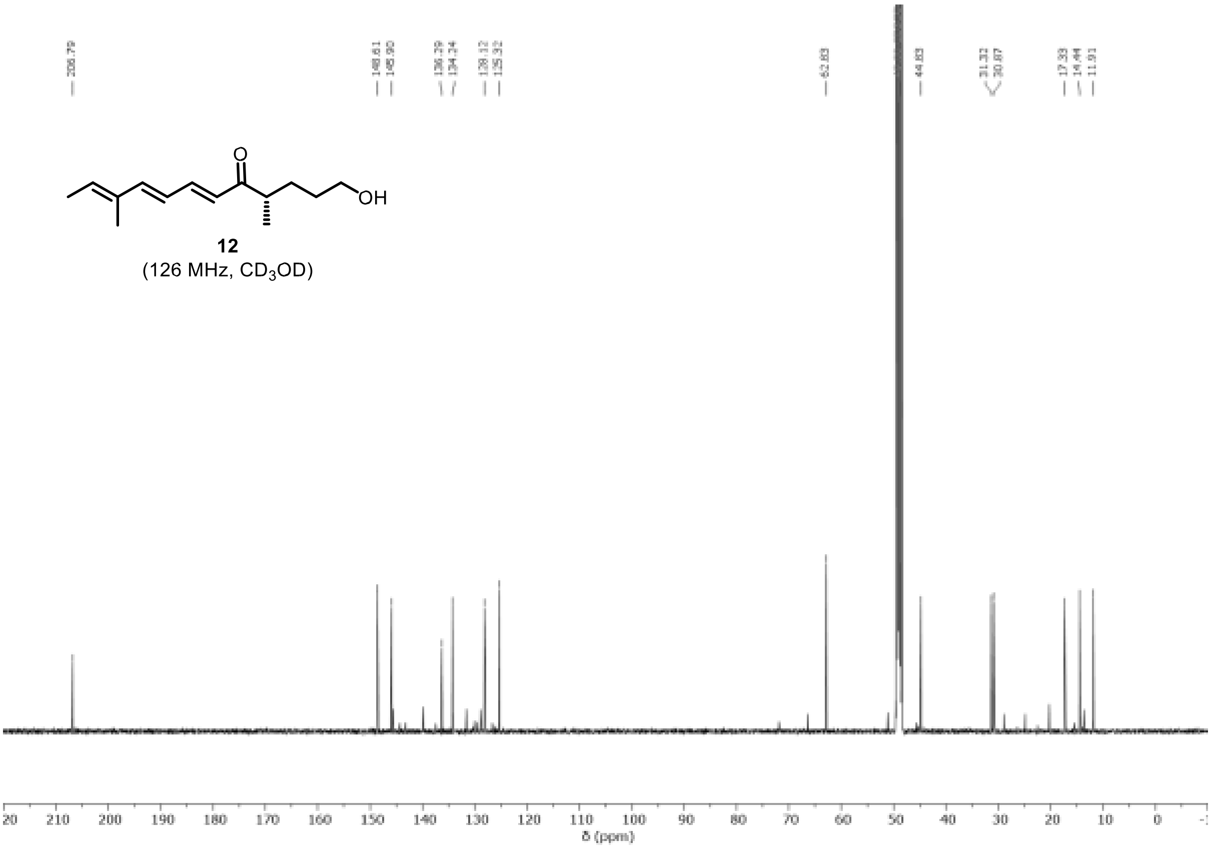

12
(126 MHz, CD_3OD)

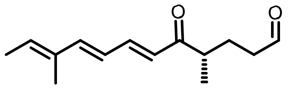

13
(500 MHz, $CDCl_3$)

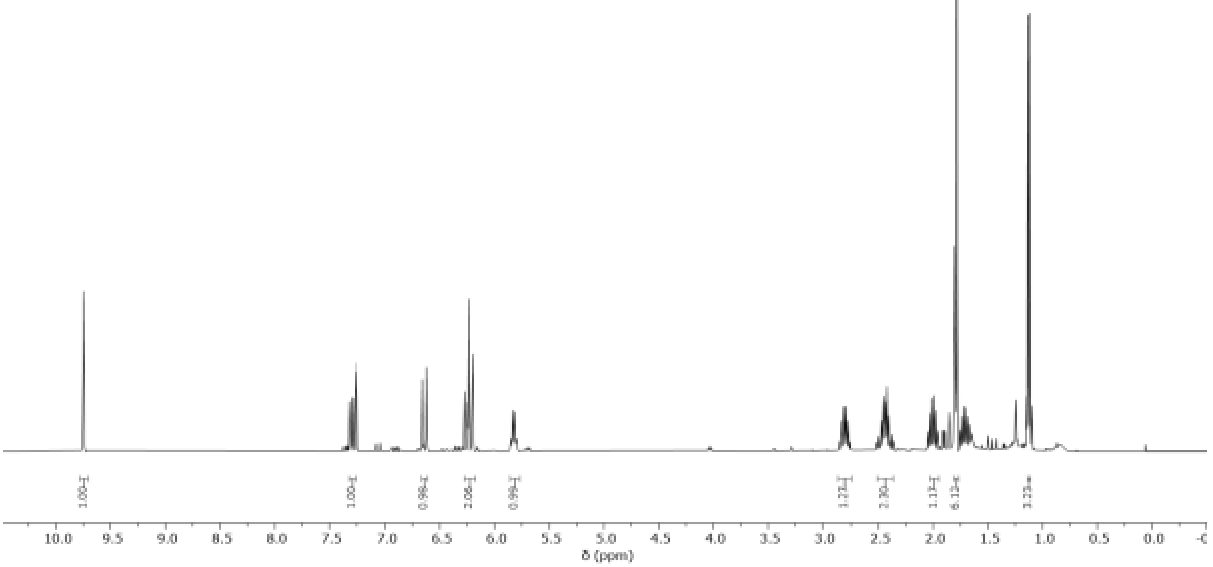

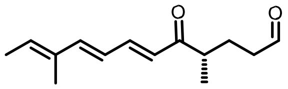

13
(126 MHz, $CDCl_3$)

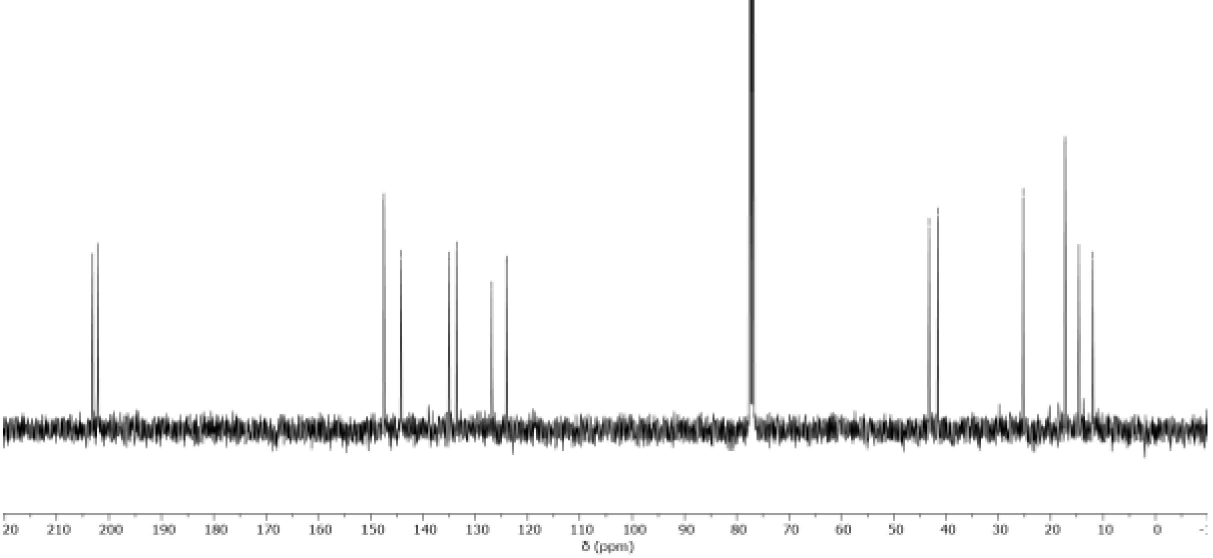

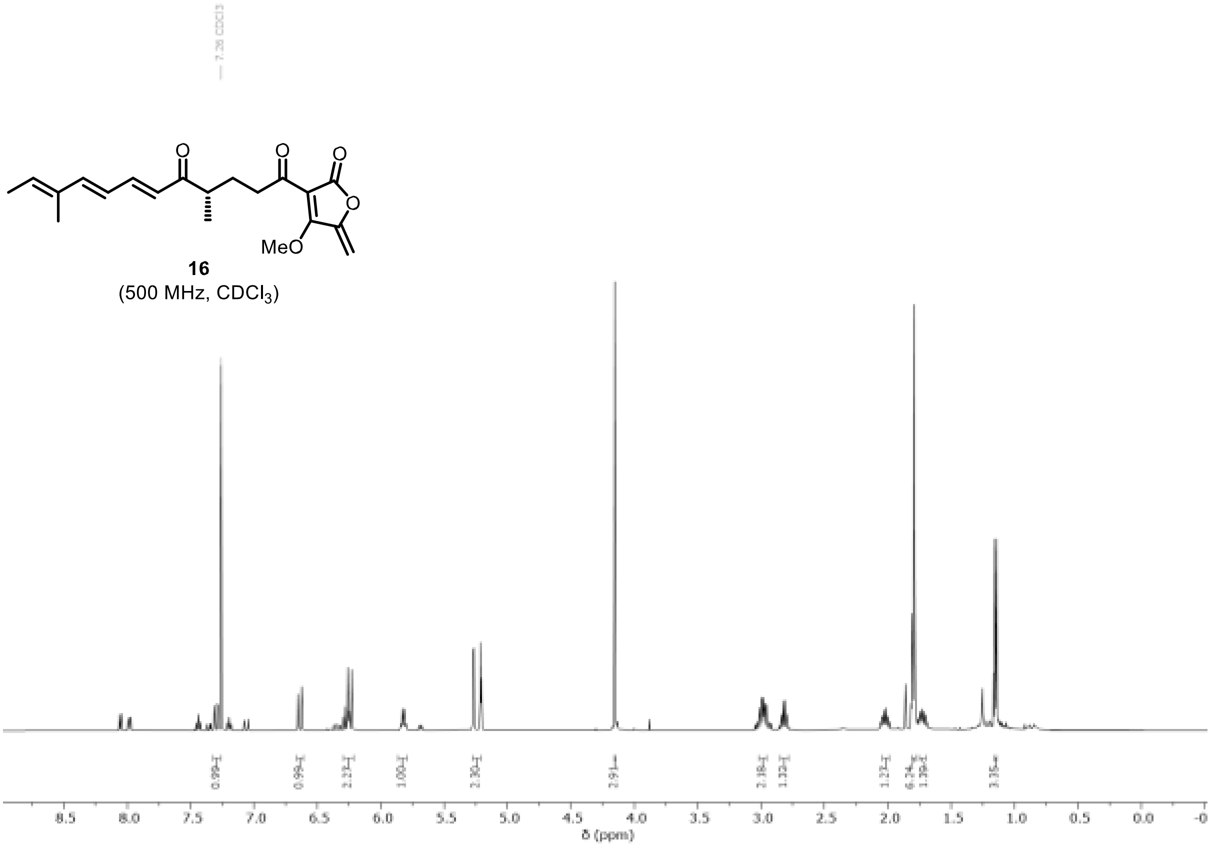

16
(500 MHz, $CDCl_3$)

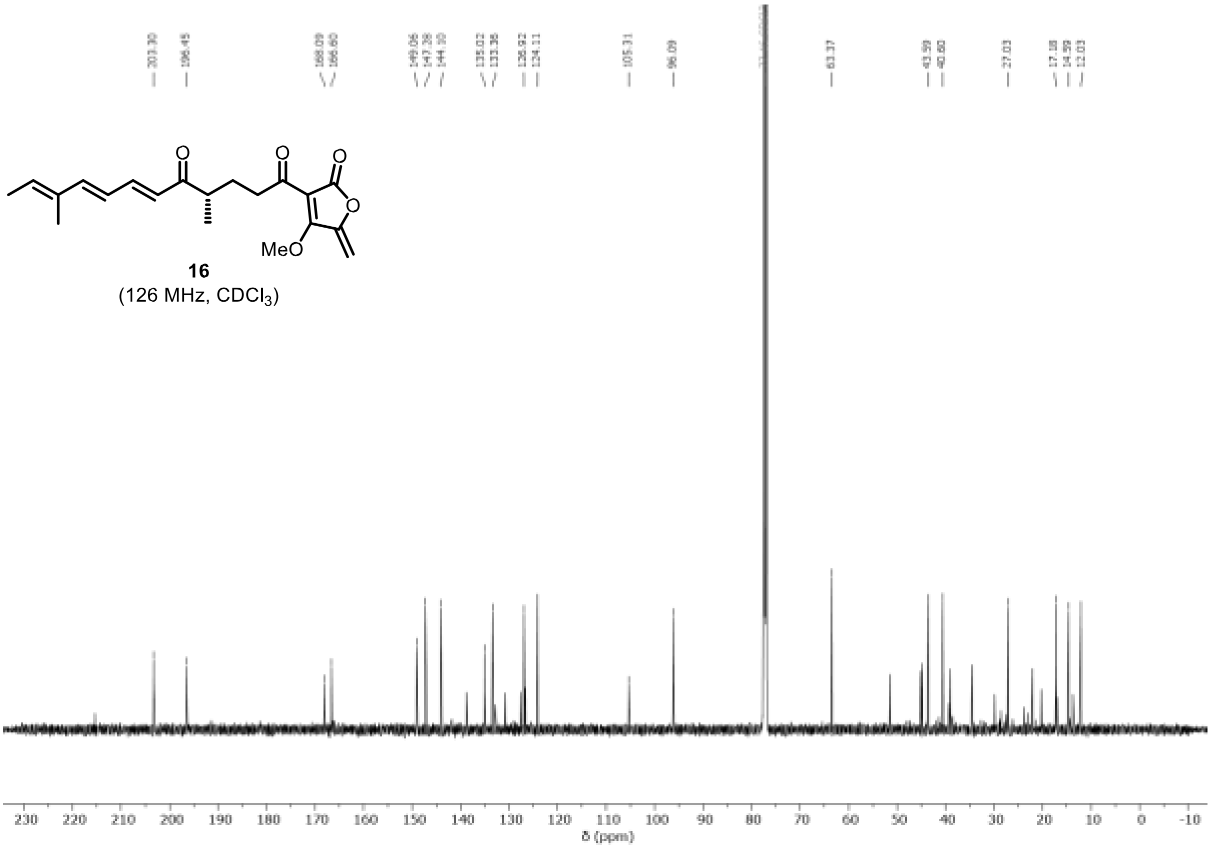

16
(126 MHz, $CDCl_3$)

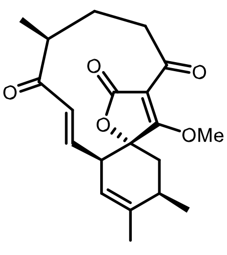

19
(500 MHz, $CDCl_3$)

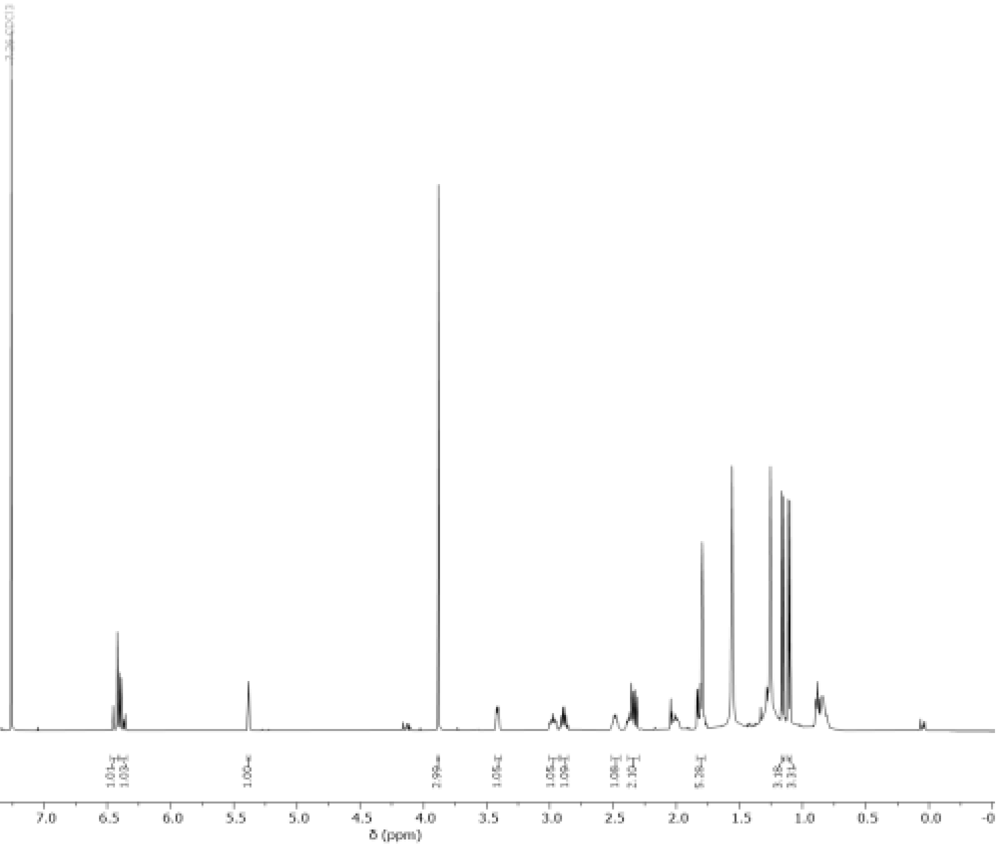

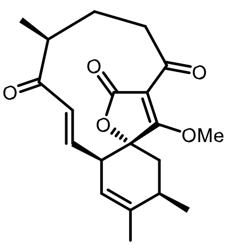

19
(126 MHz, $CDCl_3$)

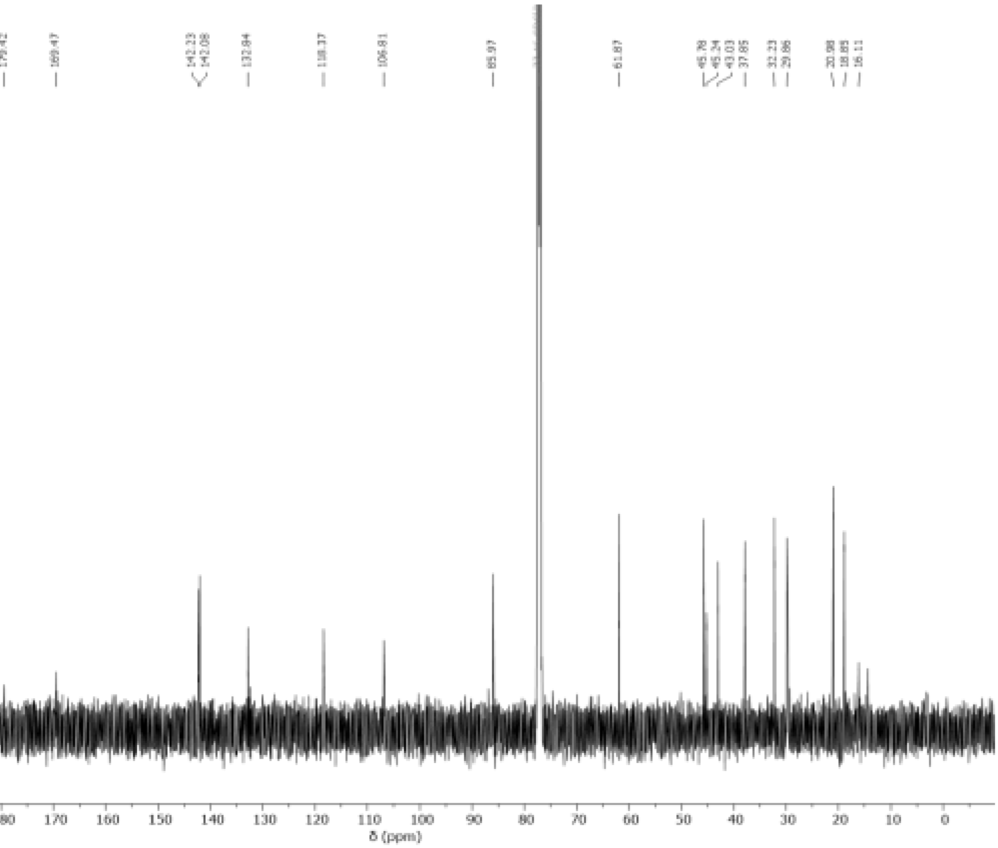

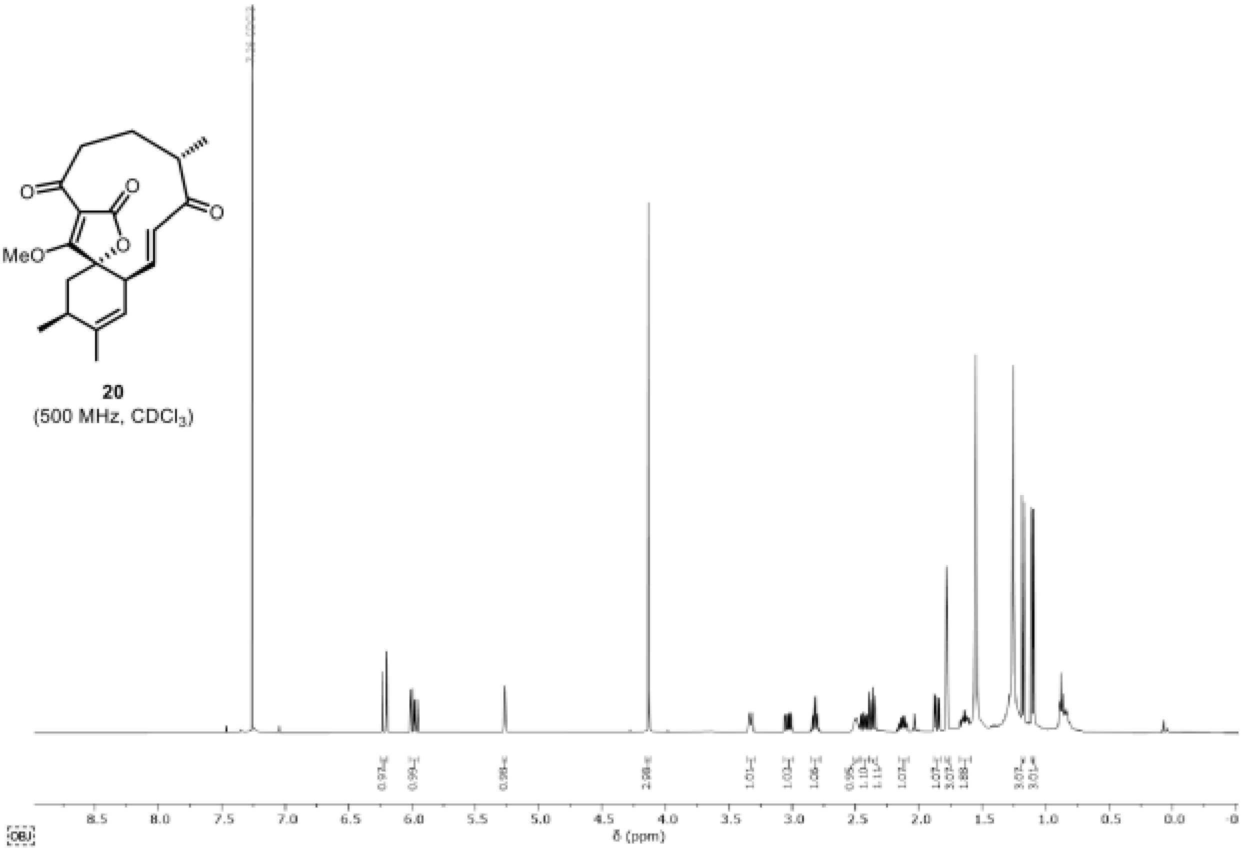

20
(500 MHz, $CDCl_3$)

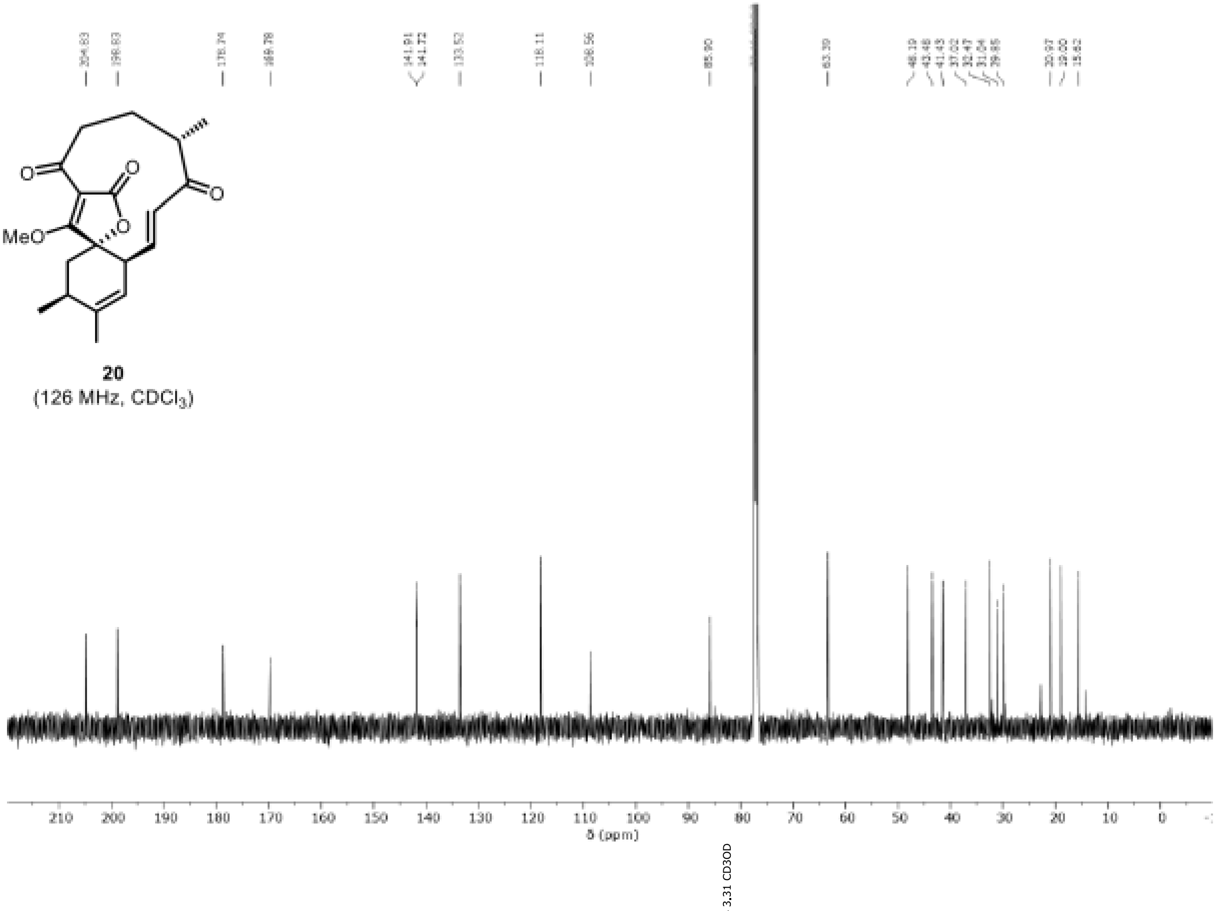

20
(126 MHz, $CDCl_3$)

— 3.31 CD3OD

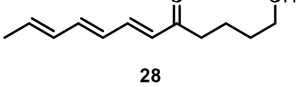

28
(400 MHz, CD_3OD)

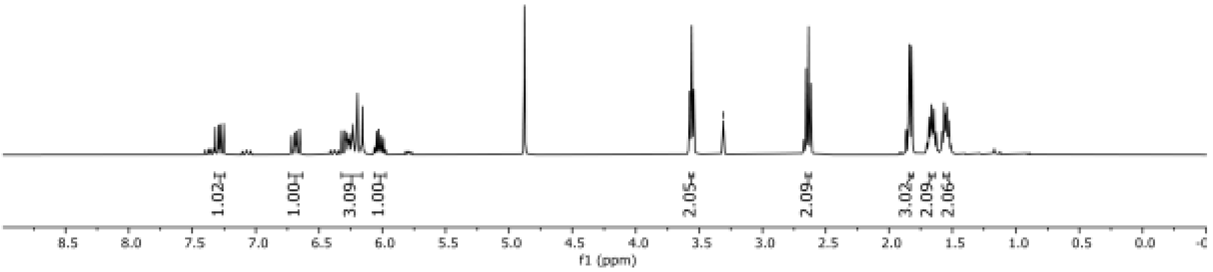

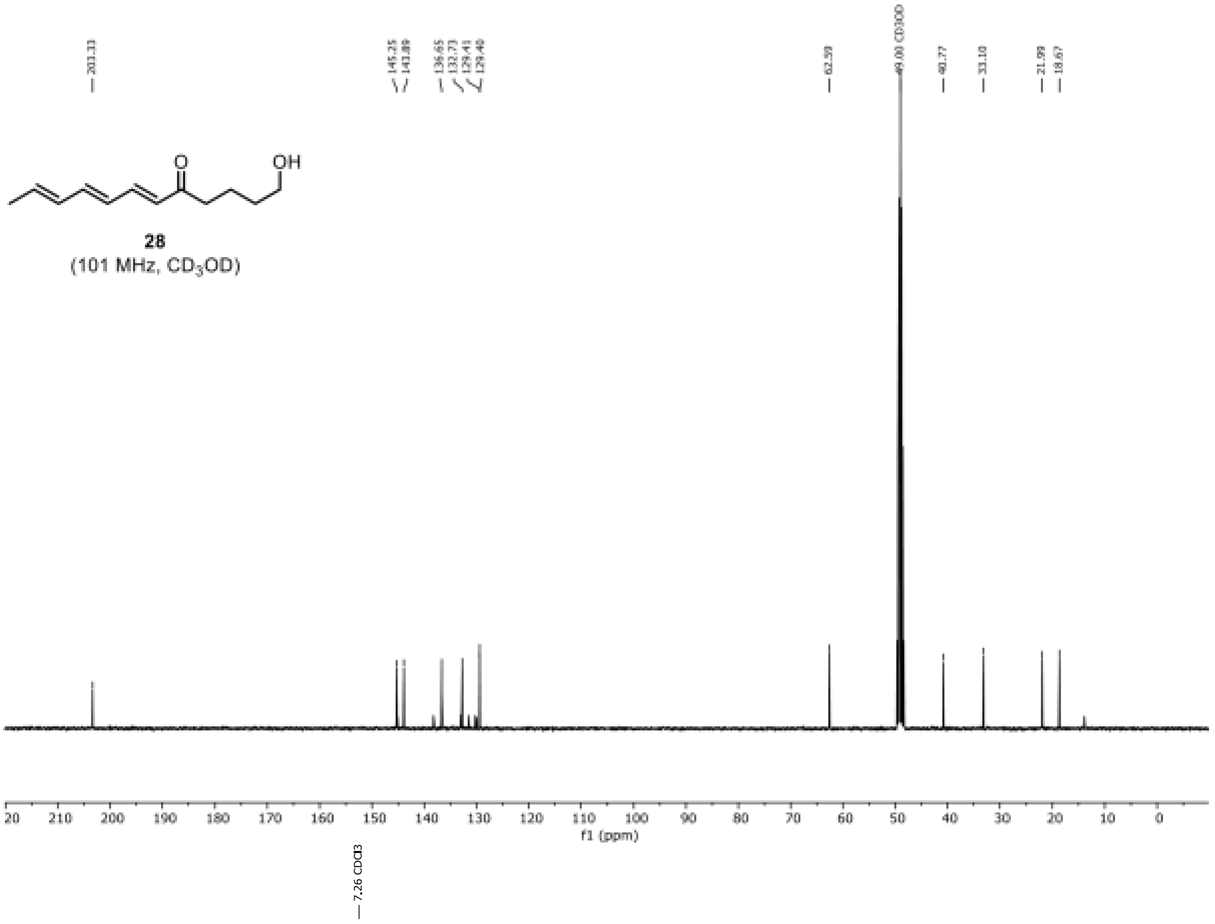

28
(101 MHz, CD_3OD)

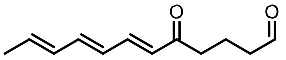

29
(400 MHz, CD_3OD)

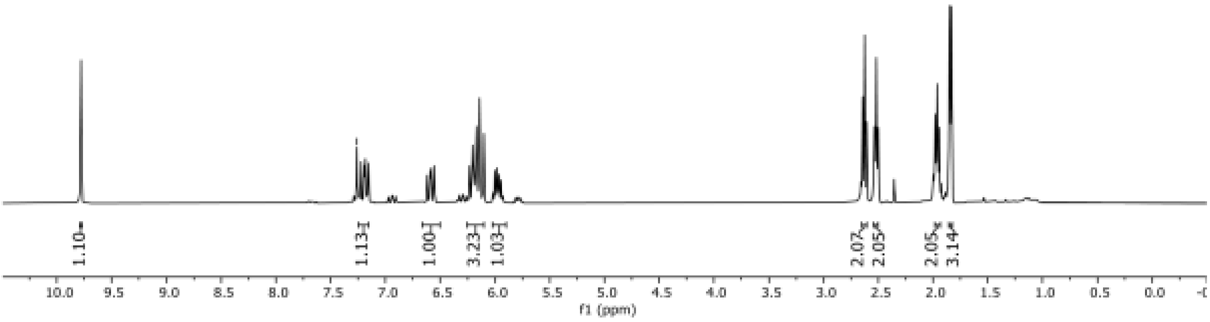

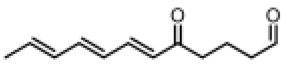

29
(101 MHz, CD_3OD)

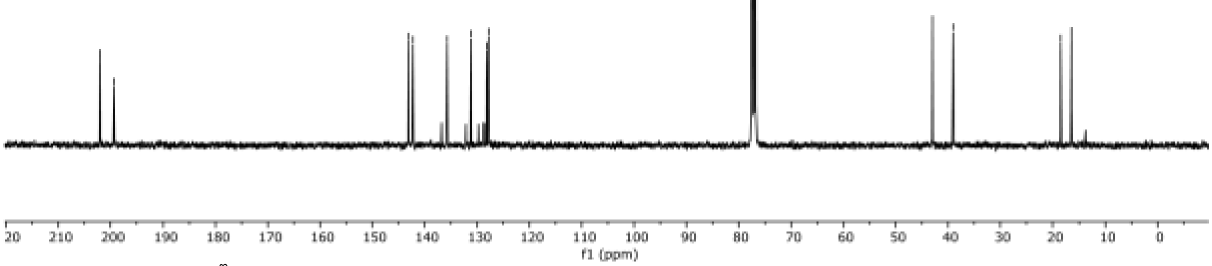

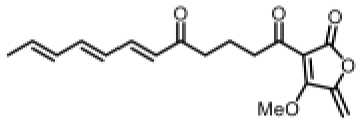

21
(400 MHz, $CDCl_3$)

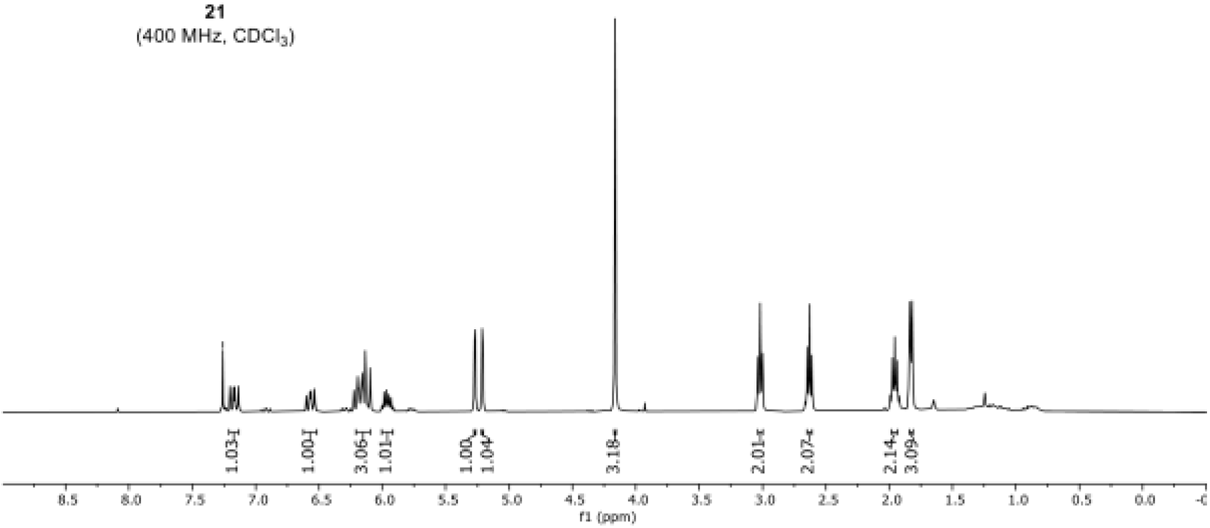

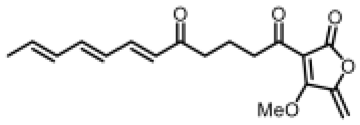

21
(101 MHz, $CDCl_3$)

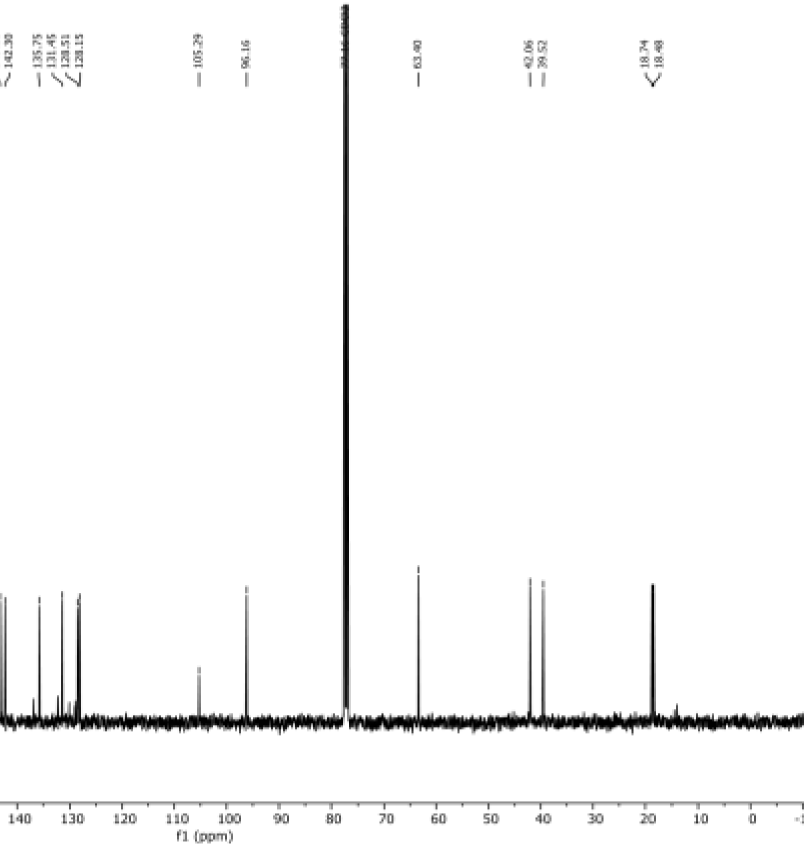

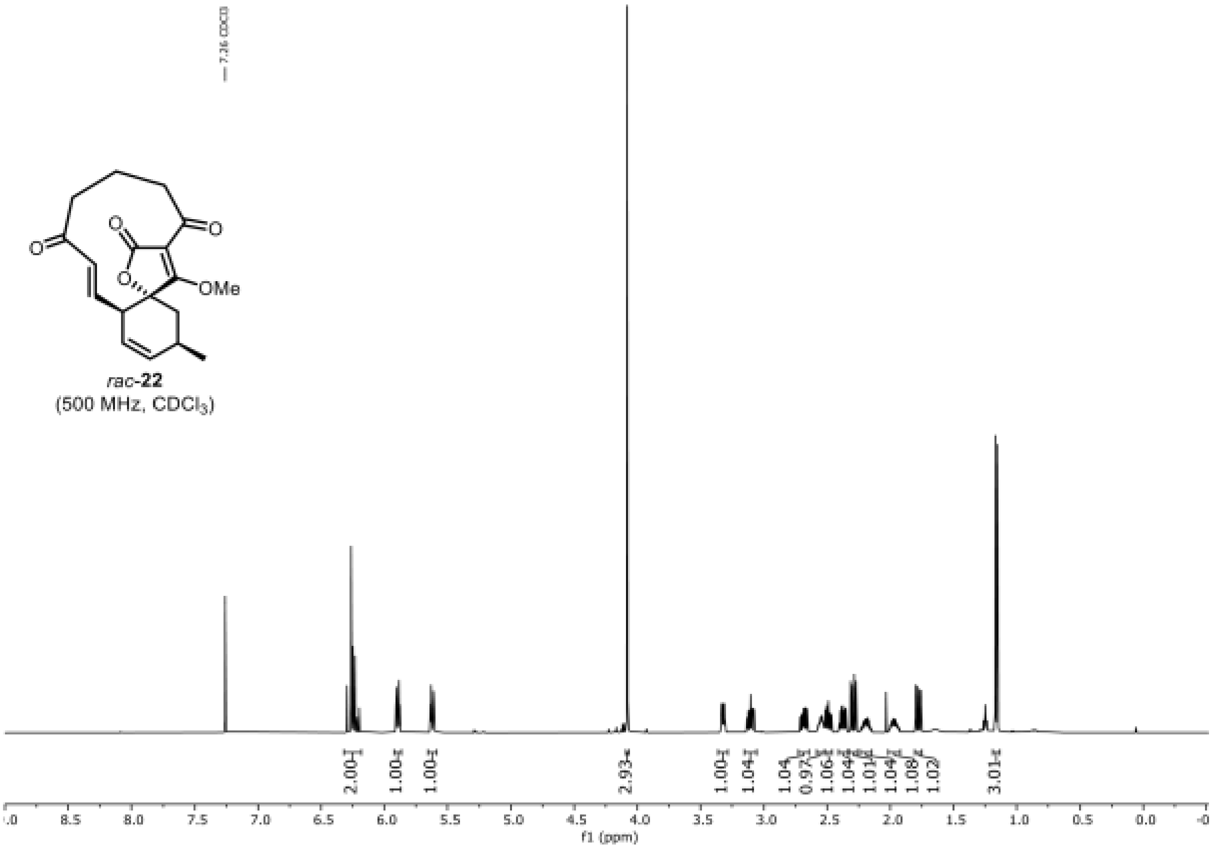

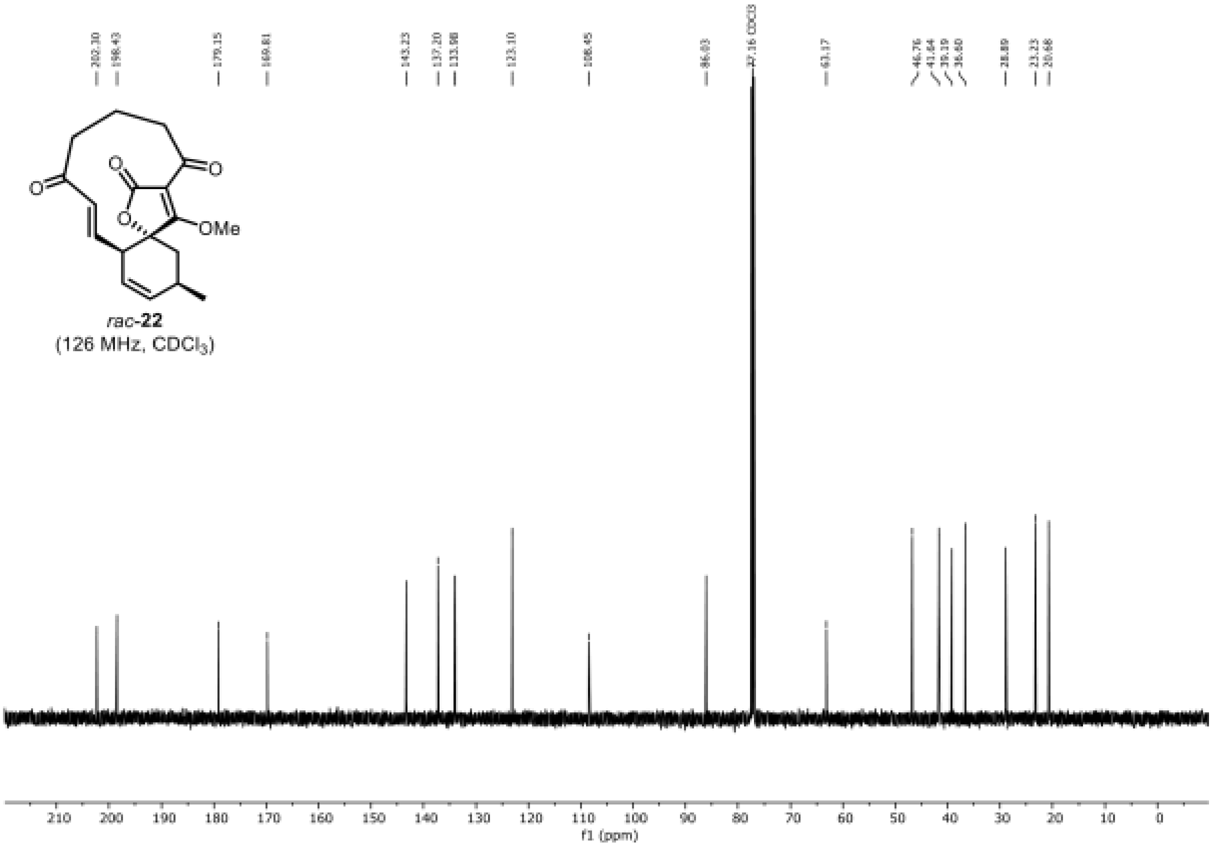

5. Supplementary References

1 N. S. Berrow, D. Alderton, S. Sainsbury, J. Nettleship, R. Assenberg, N. Rahman, D. I. Stuart and R. J. Owens, *Nucleic Acids Res.*, 2007, **35**, e45.

2 Z. Dauter and M. Dauter, *Structure*, 2001, **9**, R21–R26.

3 G. Winter, *J. Appl. Crystallogr.*, 2010, **43**, 186–190.

4 G. Winter, D. G. Waterman, J. M. Parkhurst, A. S. Brewster, R. J. Gildea, M. Gerstel, L. Fuentes-Montero, M. Vollmar, T. Michels-Clark, I. D. Young, N. K. Sauter and G. Evans, *Acta Crystallogr. Sect. D Struct. Biol.*, 2018, **74**, 85–97.

5 M. D. Winn, C. C. Ballard, K. D. Cowtan, E. J. Dodson, P. Emsley, P. R. Evans, R. M. Keegan, E. B. Krissinel, A. G. W. Leslie, A. McCoy, S. J. McNicholas, G. N. Murshudov, N. S. Pannu, E. A. Potterton, H. R. Powell, R. J. Read, A. Vagin and K. S. Wilson, *Acta Crystallogr. Sect. D Biol. Crystallogr.*, 2011, **67**, 235–242.

6 P. Skubák and N. S. Pannu, *Nat. Commun.*, 2013, **4**, 2777.

7 P. Skubák, D. Araç, M. W. Bowler, A. R. Correia, A. Hoelz, S. Larsen, G. A. Leonard, A. A. McCarthy, S. McSweeney, C. Mueller-Dieckmann, H. Otten, G. Salzman and N. S. Pannu, *IUCrJ*, 2018, **5**, 166–171.

8 P. Emsley, B. Lohkamp, W. G. Scott and K. Cowtan, *Acta Crystallogr. Sect. D Biol. Crystallogr.*, 2010, **66**, 486–501.

9 G. N. Murshudov, P. Skubák, A. A. Lebedev, N. S. Pannu, R. A. Steiner, R. A. Nicholls, M. D. Winn, F. Long and A. A. Vagin, *Acta Crystallogr. Sect. D Biol. Crystallogr.*, 2011, **67**, 355–367.

10 E. F. Pettersen, T. D. Goddard, C. C. Huang, G. S. Couch, D. M. Greenblatt, E. C. Meng and T. E. Ferrin, *J. Comput. Chem.*, 2004, **25**, 1605–1612.

11 N. A. Baker, D. Sept, S. Joseph, M. J. Holst and J. A. McCammon, *Proc. Natl. Acad. Sci.*, 2001, **98**, 10037–10041.

12 T. J. Dolinsky, P. Czodrowski, H. Li, J. E. Nielsen, J. H. Jensen, G. Klebe and N. A. Baker, *Nucleic Acids Res.*, 2007, **35**, W522–W525.

13 C. R. Søndergaard, M. H. M. Olsson, M. Rostkowski and J. H. Jensen, *J. Chem. Theory Comput.*, 2011, **7**, 2284–2295.

14 M. H. M. Olsson, C. R. SØndergaard, M. Rostkowski and J. H. Jensen, *J. Chem. Theory Comput.*, 2011, **7**, 525–537.

15 A. B. Pangborn, M. A. Giardello, R. H. Grubbs, R. K. Rosen and F. J. Timmers, *Organometallics*, 1996, **15**, 1518–1520.

16 W. C. Still, M. Kahn and A. Mitra, *J. Org. Chem.*, 1978, **43**, 2923–2925.

17 A. G. Griesbeck and A. de Kiff, *Org. Lett.*, 2013, **15**, 2073–2075.

18 J. Llaveria, Á. Beltrán, W. M. C. Sameera, A. Locati, M. M. Díaz-Requejo, M. I. Matheu, S. Castillón, F. Maseras and P. J. Pérez, *J. Am. Chem. Soc.*, 2014, **136**, 5342–5350.

19 Z. Jia, Q. Zhou, Q. Zhou, P. Chen and Y. Chen, *Angew. Chem. Int. Ed.*, 2011, **50**, 8638–8641.

20 A. D. Fotiadou and A. L. Zografos, *Org. Lett.*, 2011, **13**, 4592–4595.

21 S. Meiries, A. Bartoli, M. Decostanzi, J.-L. Parrain and L. Commeiras, *Org. Biomol. Chem.*, 2013, **11**, 4882.

22 D. A. Evans, M. D. Ennis and D. J. Mathre, *J. Am. Chem. Soc.*, 1982, **104**, 1737–1739.

23 A. J. Devine, A. E. Parnell, C. R. Back, N. R. Lees, S. T. Johns, A. Z. Zulkepli, R. Barringer, K. Zorn, J. E. M. Stach, M. P. Crump, M. A. Hayes, M. W. van der Kamp, P. R. Race and C. L. Willis, *Angew. Chem. Int. Ed.*, 2023, **62**, e202213053.

24 B. John and A. Sali, *Nucleic Acids Res.*, 2003, **31**, 3982–3992.

25 O. Trott and A. J. Olson, *J. Comput. Chem.*, 2010, **31**, 455–461.
